# Targeting Innate Receptors with MIS416 Reshapes Th Responses and Suppresses CNS Disease in a Mouse Model of Multiple Sclerosis

**DOI:** 10.1371/journal.pone.0087712

**Published:** 2014-01-31

**Authors:** Madeleine White, Gill Webster, David O’Sullivan, Sarrabeth Stone, Anne Camille La Flamme

**Affiliations:** 1 School of Biological Sciences, Victoria University of Wellington, Wellington, New Zealand; 2 Innate Immunotherapeutics, Auckland, New Zealand; 3 Malaghan Institute of Medical Research, Wellington, New Zealand; Charite Universitätsmedizin Berlin, Germany

## Abstract

Modification of the innate immune cell environment has recently been recognized as a viable treatment strategy for reducing autoimmune disease pathology. MIS416 is a microparticulate immune response modifier that targets myeloid cells, activating cytosolic receptors NOD2 and TLR9, and has completed a phase 1b/2a trial for the treatment of secondary progressive multiple sclerosis. Using a mouse model of multiple sclerosis, we are investigating the pathways by which activation of TLR9 and NOD2 may modify the innate immune environment and the subsequent T cell-mediated autoimmune responses. We have found that MIS416 has profound effects on the Th subset balance by depressing antigen-specific Th1, Th17, and Th2 development. These effects coincided with an expansion of specific myeloid subpopulations and increased levels of MIS416-stimulated IFN-γ by splenocytes. Additionally, systemic IFN-γ serum levels were enhanced and correlated strongly with disease reduction, and the protective effect of MIS416 was abrogated in IFN-γ-deficient animals. Finally, treatment of secondary progressive MS patients with MIS416 similarly elevated the levels of IFN-γ and IFN-γ–associated proteins in the serum. Together, these studies demonstrate that administration of MIS416, which targets innate cells, reshapes autoimmune T cell responses and leads to a significant reduction in CNS inflammation and disease.

## Introduction

Activation of innate pattern recognition receptors (PRR) has been implicated in both the pathogenesis as well as the regulation of multiple sclerosis (MS) and its animal model, experimental autoimmune encephalomyelitis (EAE) [1], [2], [3], [4]. Studies have shown that signaling through PRRs such as TLR2 or TLR4 is necessary for the induction of EAE as deficiency in these TLR or TLR-adaptor MyD88-mediated signaling inhibits EAE induction. The precise involvement of TLR9 in establishing disease remains controversial, as administration of TLR9 ligands exacerbates EAE disease in some studies [4], [5], [6], while another study suggests a possible protective role for TLR9 signaling in EAE [7]. In addition, recent studies suggest that NOD-RICK signaling is another important mediator of EAE [3]. Although the role that NOD2 signaling may play in the induction or pathogenesis of MS is less clear [7], the association between bacterial infections and MS as well as the detection of antigen presenting cells containing peptidoglycan, a source of NOD2 ligand, in the brains of MS patients support a possible pathogenic role for this PRR in MS [8].

In contrast to this pathogenic role, recent evidence also points to the potential therapeutic application of PRR agonists as immunomodulatory agents to treat MS. For example, multiple studies have demonstrated that administration of TLR3 and TLR7 ligands have a protective effect in EAE [9], [10], [11]. In particular, the suppression of EAE by TLR3 activation has been attributed directly to the induction of endogenous IFN-β [10] and similar studies by O’Brien *et al.* found that the protective TLR7 agonist, imiquimod, also enhanced IFN-β production during EAE [9]. In contrast to this mechanism, recent work has found that multiple low doses of a TLR7 agonist induced a tolerogenic state that was able to suppress CNS inflammation during EAE [11]. This finding suggests that repeated induction of low levels of inflammatory cytokine signaling may favor the enhancement of negative feedback immune mechanisms that effectively downregulate inflammatory T cell activity [11].

MIS416 is a novel microparticle derived from *Propionibacterium acnes* comprising a minimal cell wall skeleton rich in immunostimulatory muramyl dipeptide (MDP) along with bacterial single stranded DNA which signal through cytosolic receptors NOD2 and TLR9, respectively [12]. While originally developed as a vaccine adjuvant [12], interest has grown around the potential use of MIS416 as a stand-alone immunomodulatory agent for treatment of inflammatory disorders when administered systemically. Based on positive anecdotal findings from a compassionate use program in secondary progressive MS patients, a formal phase 1b/2a trial has been conducted to evaluate the safety, tolerability and initial impact of MIS416 in secondary progressive MS following systemic administration. To determine how MIS416-mediated NOD2 and TLR9 signaling may modulate immune responses during MS, we used the EAE mouse model of MS to determine the effect of MIS416 treatment on the development of T cell-mediated autoimmune responses.

## Materials and Methods

### Animals

Female C57BL/6 and IFN-γ-deficient mice were obtained from the Malaghan Institute of Medical Research and used between 8–12 weeks of age. SJL/J mice (originally from the Animal Resource Centre, Canning Vale, WA, Australia) were bred and housed in the animal facility at Victoria University of Wellington, New Zealand.

### Ethics Statement

All of the experiments with animals were carried out in the School of Biological Sciences Animal Facility at Victoria University of Wellington and were approved by the Victoria University of Wellington Animal Ethics Committee (protocols 2008-R12 and 2011-R21). The clinical trial was approved by the New Zealand Medicines and Medical Devices Safety Authority (protocol MIS416-201) and registered on the Clinicaltrials.gov website; the reference is http://clinicaltrials.gov/ct2/show/NCT01191996.

### EAE Induction and Treatments

Mice were immunized s.c. in the rear flanks with myelin oligodendrocyte glycoprotein (MOG)_35–55_ peptide (50 µg/mouse; Genescript, Piscataway, NJ) in Freund’s adjuvant (Sigma, St. Louis, MO) containing 500 µg/mouse *Mycobacterium tuberculosis* (Fort Richard, Auckland, New Zealand). Additionally, mice were injected i.p. with pertussis toxin (200 ng/mouse; List Biochemicals, Campbell, CA) on days 0 and 2. For the SJL/J experiments, mice were immunized s.c. in the rear flanks with PLP_139–151_ peptide (100 µg/mouse; Genscript) and heat-inactivated *M. tuberculosis* (500 µg/mouse) in incomplete Freund’s adjuvant followed by 100 ng/mouse pertussis toxin i.p. on days 0 and 2. Mice were weighed and scored daily as follows: 0, normal; 1, partial tail paralysis; 2, full tail paralysis; 3, paralysis in one hind limb; 4, paralysis in both hind limbs; and 5, moribund. MIS416 (Innate Immunotherapeutics, Auckland, New Zealand) was administered i.v. into the tail vein.

### Isolation and in vitro Culture of Cells

Single cell suspensions of splenocytes and lymph node cells were isolated as described [13], and the number of viable cells were counted using the typan blue exclusion assay. Cells were cultured (10^6^ cells/ml) in complete medium containing Dulbecco’s minimal essential medium, 10% FCS, 100 U/ml penicillin plus 100 µg/ml streptomycin, 10 mM Hepes, 2 mM L-glutamine, and 50 µM 2-mercaptoethanol (all from Invitrogen, Carlsbad, CA) and stimulated with MOG peptide (27 µg/ml) or MIS416 (20 µg/ml) for 72 or 48 hours, respectively. These doses were based upon previously published dose-response curves for MOG [14], [15] and MIS416 [12].

### Cytokine Assays

Murine cytokines in the serum were assessed by Th1/Th2/Th17 10-plex cytokine bead assay (CBA; Bender MedSystems, Vienna, Austria) and culture supernatants by the Th1/Th2/Th17/Th22 13-plex CBA kit (Bender MedSystems) according to the manufacturer’s instructions. For quantification of cytokine levels in human serum, heparin anti-coagulated peripheral blood was processed to obtain serum that was immediately frozen and stored at −80^o^C until analysis. Cytokines were assessed using a custom CBA matrix (Becton Dickinson CBA Flex Sets ™) and by ELISA (BD Biosciences) and quantification was performed according to the manufacturers instructions.

### Flow Cytometric Analysis

Single cell suspensions of splenocytes and lymph node cells were stained and run on a FACS Canto II flow cytometer (BD, Franklin Lakes, NJ) as described. Antibodies used for the phenotypic analyses include: rat anti-CD4, rat anti-CD11b, rat anti-Gr-1, and rat anti-FoxP3 from BD Biosciences (San Jose, CA) and rat anti-CD25, rat anti-CD205, rat anti-CD14, and hamster anti-CD11c from BioLegend (San Diego, CA). Rat anti-F4/80 antibody was purchased from eBioscience (San Diego, CA). For intracellular cytokine analyses, GolgiStop (1 µg/10^6^ cells; BD Biosciences), PMA (50 ng/ml; Sigma) and ionomycin (500 ng/ml; Sigma) were added to the MOG-stimulated splenocyte cultures 4 hours before antibody staining. Fcγ receptors were blocked by addition of purified anti-CD16/32 antibodies (Fc Block; 1 µg/10^6^ cells; BD Biosciences) just prior to surface marker staining. After extracellular staining, cells were fixed with 4% paraformaldehyde and permeabilized with 0.1% saponin buffer containing 0.1% bovine serum albumin. Intracellular cytokines were detected using rat anti-IFN-γ and rat anti-interleukin (IL)-17A antibodies (BD Biosciences). Specificity was confirmed using the fluorescently-labeled isotype control antibodies recommended by the manufacturer. Gating strategy for intracellular cytokine analysis is shown in Figure S1e. All flow cytometric data were analyzed by FlowJo software (Tree Star Inc., Ashland, OR). Total cell numbers were calculated by multiplying the % of the specific cell population as assessed by flow cytometric analysis with the total number of viable cells at isolation determined by trypan dye exclusion.

### Clinical Trial – participants and Protocol

**Figure 1:**
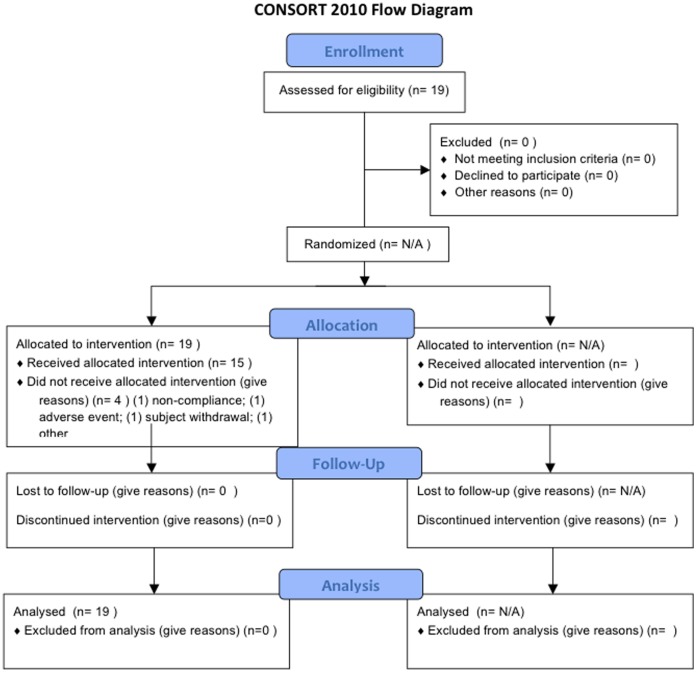
CONSORT flow chart for MIS416-201.

MIS416 was evaluated in a single center, open-label, non-randomized, dose-escalation study. For protocol details and CONSORT checklist, see Information S1 and Information S2, respectively. The date ranges for the trial form first patient enrolment to late patient completed are 24/09/10 to 29/06/12. Briefly, the primary objectives were to determine the safety and tolerability, dose-limiting toxicities, maximum tolerated dose, and recommended phase 2 dose of MIS416 administered i.v. once weekly for 4 doses in patients with progressive MS. A secondary objective was to assess the pharmacodynamic effects of MIS416 based on analysis of blood serum samples for known MIS416-inducible immune factors. The reference for this trial on http://clinicaltrials.gov is NCT01191996. MIS416 was administered i.v over 10 minutes once weekly at doses of 125 to 1000 µg/week. Main criteria for inclusion: subject at least 18 years of age, male or female and any ethnicity; to be classified as having either primary progressive MS or secondary progressive MS with worsening clinical status within the past 2 years; and an Expanded Disability Status Scale of 2.5 to 7.0 at screening. Key exclusion criteria for enrolment included: relapsing-remitting MS or progressive-relapsing MS; any immunomodulatory drug therapy or immunosuppressive therapy within the previous six months, or vaccine or systemic corticosteroids within the previous 60 days, prior to initiation of study drug; and exposure to other experimental treatments currently under investigation in MS clinical trials including alemtuzamab, rituximab, fingolimod, and cladribine. Safety assessments included characterization of dose-limiting toxicities (type, incidence, severity, timing, seriousness, and relationship to treatment of adverse events); effects on vital signs and laboratory parameters; changes in electrocardiograms (ECGs) and ophthalmologic examinations; and safety MRI assessments before, during and following completion of MIS416 therapy. For the CONSORT flow chart, see Figure 1.

### Statistical Analyses

All data were analysed using Prism GraphPad (La Jolla, CA). Time and drug effects were tested by 2-way ANOVA, and post tests were only performed when p<0.05 by 2-way ANOVA. Two group comparisons were assessed by Mann-Whitney (non-parametric) or Student’s t test (parametric) as indicated and multiple groups by one-way ANOVA. When required, the raw values were log transformed to normally distribute the data before applying the appropriate statistical test. p values ≤0.05 were considered significant.

## Results

### MIS416 Reduced EAE Disease Severity when Administered at Time of Immunization or at Disease Onset

**Figure 2:**
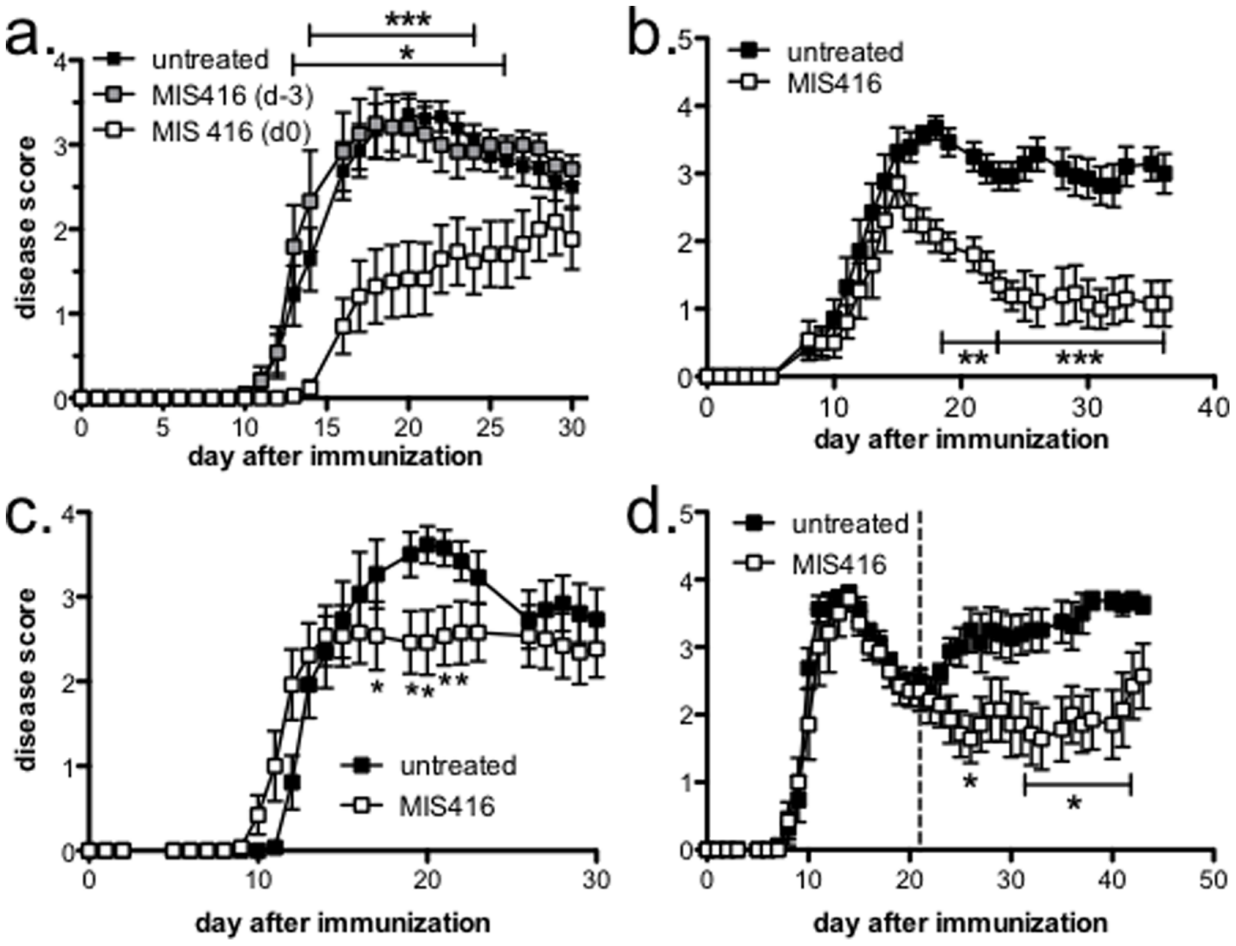
MIS416 administered at the time of immunization or after disease onset reduced the severity of EAE. A single dose of MIS416 (250 µg/mouse) reduced disease severity when administered at the time of immunization **(a**; n = 17**)** or 12 days post immunization **(c**; n = 13**)** but not 3 days before immunization **(a**; n = 12**)** compared to untreated mice (n = 21 (a) or 13 (c)). (**b**) Multiple low doses of MIS416 (100 µg/mouse; n = 13) provided a consistent reduction in disease severity compared to untreated mice (n = 14). (**d**) MIS416 was effective at reducing disease when administered weekly after the first episode (50 µg/mouse starting day 21; dashed line) in the relapsing-remitting SJL/J model. Mice were immunized to induce EAE (see Methods for C57BL/6 mice (a–c) and SJL.J mice (d)), treated by i.v. administration with a single **(a** and **c)** or multiple doses **(b** and **d)** of MIS416, and scored daily. Shown are the means and SEM of disease scores from mice from 3 (a–c) or 2 (d) independent experiments. **(a** and **b)** p<0.001; untreated compared to MIS416 d0 treatment by 2-way ANOVA. (**c**) p<0.05; untreated compared to MIS416 d12 treatment by 2-way ANOVA. (**d**) p<0.05; untreated compared to MIS416 treatment by 2-way ANOVA. * p<0.05; ** p<0.01; and ***p<0.001 by Bonferroni’s multiple comparison post-test.

To determine if MIS416, a microparticle containing TLR9 and NOD2 ligands, had any effect on the development of EAE, 250 µg of MIS416 was administered i.v. to mice either 3 days prior to, or immediately before immunization. Administration of MIS416 on the day of immunization (d0) resulted in a reduction in incidence, delay of disease onset, and a reduction in disease severity (Figure 2a; Table 1). In contrast to d0 MIS416 treatment, mice injected with MIS416 3 days prior to immunization (d-3) exhibited no difference in disease expression compared to untreated mice (Figure 2a; Table 1). Additionally, we assessed whether lower doses of MIS416 (50 and 100 µg) would similarly reduce disease but found that a single dose below 250 µg had little effect on disease progression (data not shown).

**Table 1:** Disease parameters.

Treatment group	% Incidence (n)	Mean max score (SEM)	Mean day of onset (SEM)^a^
**C57BL/6 mice** **250 µg single dose**			
untreated	100 (21/21)	3.7 (0.2)	14.3 (0.7)
MIS416 d-3	100 (12/12)	4.0 (0.1)	14.4 (1.1)
MIS416 d0	76 (13/17)***	2.7 (0.4)*	19.2 (1.3)**
**C57BL/6 mice** **100 µg weekly dose**			
untreated	100 (14/14)	4.1 (0.1)	11.1 (0.7)
MIS416 d0	100 (13/13)	3.3 (0.2)**	11.9 (0.8)
**SJL/J mice** **100 µg weekly dose**			
untreated	100 (8/8)	3.9 (0.2)	NA
MIS416 d21	100 (9/9)	2.8 (0.4)*	NA

a sick mice only.

* p<0.05 compared to untreated; by 1 way ANOVA with Bonferroni’s post test (single dose) or by Mann-Whitney test (SJL/J).

** p<0.01 compared to untreated; by 1 way ANOVA with Bonferroni’s post test (single dose) or by Mann-Whitney test (weekly).

*** p<0.0001 compared to untreated; Mantel-Cox (log-rank test).

Although a single dose of MIS416 had a significant effect on EAE development when administered at the time of immunization, over time the effect of disease suppression appeared to diminish (Figure 2a). Thus, we investigated whether a weekly administration of MIS416 could provide a more sustained effect on disease reduction. Initially, mice were treated with weekly high doses of MIS416 (250 µg) starting from the day of immunization but while disease was completely inhibited, this dosing regimen caused substantial mortality (80%) most likely due to over production of inflammatory cytokines. In contrast, a weekly lower dose of MIS416 (100 µg) caused little mortality or morbidity and provided a consistent reduction in disease compared to untreated, immunized mice (Figure 2b; Table 1). Together, these findings indicate that MIS416 is effective at modifying EAE and that regular doses are required to maintain consistent protection.

Because any effective MS therapy must be able to modify pre-existing disease pathways, we assessed the ability of MIS416 to alter disease progression after disease onset in the C57BL/6 mouse model and the SJL/J model of relapsing-remitting MS. Using this approach, we found that a single MIS416 dose of 250 µg had an immediate effect on disease progression in C57BL/6 mice when administered 12 days post immunization (Figure 2c). Moreover, weekly low doses administered to SJL/J mice after the first disease episode (day 21) significantly suppressed disease relapses (Figure 2d). Overall, these results suggest that MIS416 is also effective at reducing EAE when administered after disease onset and can reduce subsequent relapses in SJL/J mice.

### MIS416 Treatment Modified the Systemic but not Local Antigen-specific Immune Response

**Figure 3:**
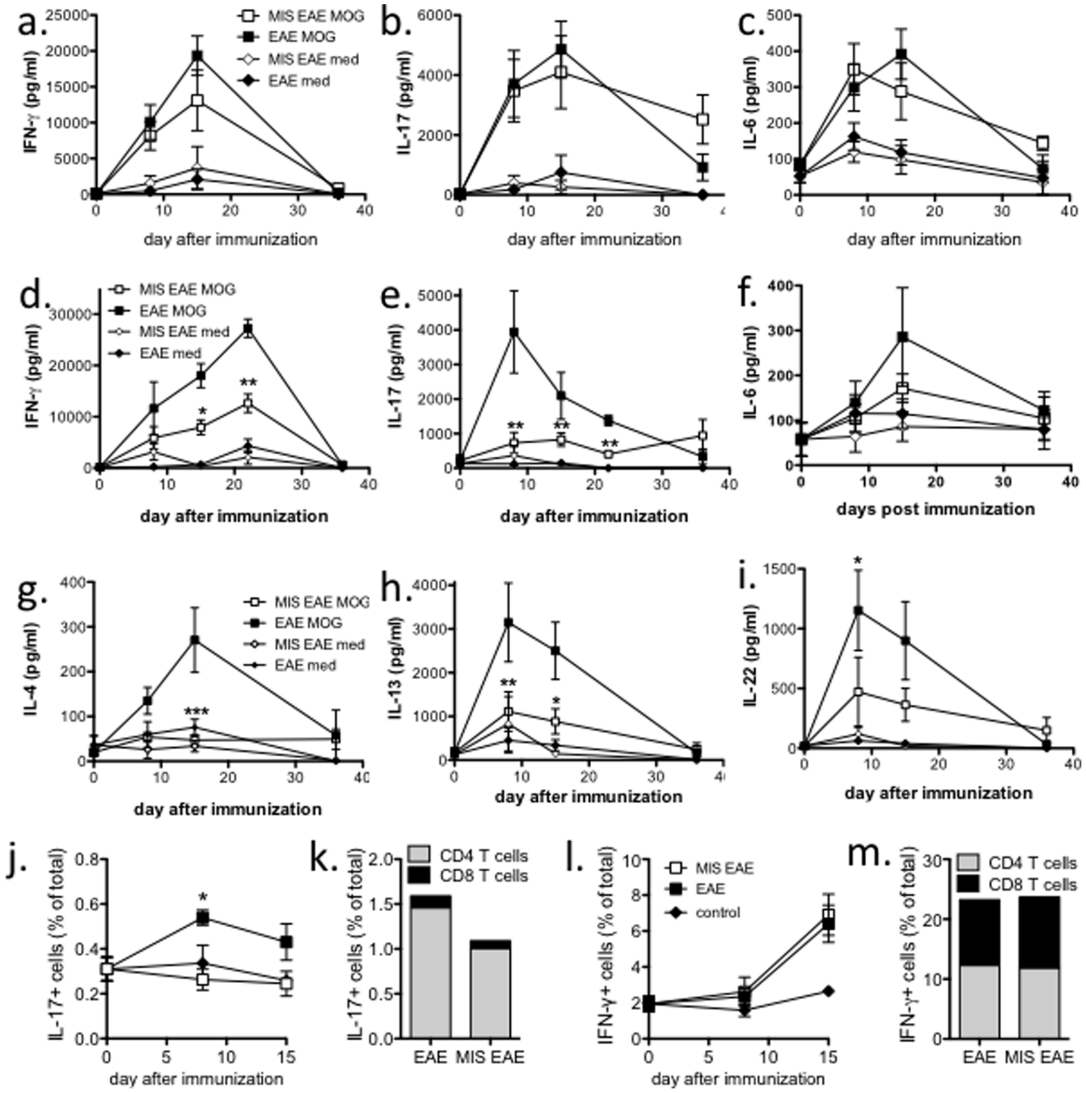
MIS416 treatment suppressed systemic but not local antigen-specific responses. C57BL/6 mice were immunized to induce EAE (see Methods) and treated weekly by i.v. administration of 100 µg/mouse MIS416 starting on the day of immunization. Lymph node cells (**a–c**) or splenocytes (**e–m**) were isolated from MIS416-treated or untreated, immunized mice and cultured (10^6^ cells/well) with MOG peptide (27 µg/ml) or medium (med) for 72 hours. (**a–i**) Cytokines in the culture supernatants were assayed by CBA and ELISA. (**j–m**) At 72 hours post-stimulation, intracellular IL-17A (**j**) and IFN-γ (**l**) levels were assessed by flow cytometry, and the percentage of cytokine positive cells expressing CD4 or CD8 in cultures from MIS416-treated and untreated, immunized animals at day 15 were evaluated and compared to untreated, unimmunized animals (control; **k** and **m**). Shown are the means and SEM of values from individual mice (n = 3-15/time point) from 2 (LN) or 5 (spleen) experiments. * p<0.05, ** p<0.01, and *** p<0.001 MOG-stimulated EAE compared to MOG-stimulated MIS EAE by 2-way ANOVA with Bonferroni’s multiple comparison post-test.

Given that immunization-induced T cells are central to establishing CNS inflammation in the EAE model, one of our aims with this study was to assess how MIS416 treatment affected antigen-specific cytokine response development. To accomplish this, we compared the MOG-specific responses of lymph node cells and splenocytes at various time points post immunization: day 8 (before onset), day 15 (peak disease), day 22 (established disease), and day 36 (chronic disease). These time points all coincided with one-day post MIS416 treatment. Mice were treated with weekly low doses (100 µg) of MIS416 starting on the day of immunization, and cytokine production after *in vitro* stimulation with MOG or with medium alone was assessed in culture supernatants. All cytokines appeared to peak between 8–22 days post immunization, and only low levels of antigen-specific cytokines were detected by day 36 indicating a contraction in the antigen-specific response. No difference in antigen-specific IFN-γ, IL-17, and IL-6 production by lymph node cells was found at any time point post-immunization (Figure 3a–c) and no antigen-specific cytokines were detected in culture supernatants from untreated (day 0 in Figures 3a–c) and MIS416-treated, unimmunized mice (data not shown). Similar levels of MOG-specific IL-22 were also found (data not shown). Additionally, no antigen-specific IL-4, IL-5, IL-10, IL-1, IL-21, IL-27 or IL-2, and only low levels of MOG-specific TNF-α were detected (data not shown). These findings indicate that strong, local antigen-specific Th1 and Th17 cytokine responses develop normally in the draining lymph nodes of all immunized mice regardless of MIS416 administration.

Although no difference was found in the recall responses of lymph node cells, a profound reduction in antigen-specific Th17-associated and Th2-associated cytokines was observed in splenocyte cultures from MIS416-treated, immunized mice compared to untreated, immunized mice (Figure 3 e–i). In particular, IL-17 and IL-22 (Th17) as well as IL-4 and IL-13 (Th2) were significantly reduced in MIS416-treated mice. In contrast, only low levels of MOG-specific IL-6 were detected (Figure 3f), and there was no difference in MOG-specific TNF-α production (data not shown). While MOG-specific IFN-γ production was not altered by MIS416 treatment 8 days post immunization, the treatment lead to a significant reduction by days 15 and 22 (Figure 3d). Overall, the kinetics of cytokine production by splenocytes was similar to lymph node cultures in untreated, immunized mice. Splenocytes from unimmunized mice did not produce antigen-specific cytokines (day 0 in Figures 3d-i for untreated, unimmunized cytokine production) nor did MIS416-treated, unimmunized mice (data not shown). No antigen-specific IL-2, IL-10, IL-21, IL-27, or IL-1 was detected in immunized cultures. These results suggest that MIS416 treatment alters systemic immune responses, as seen in the spleen, by completely suppressing Th17- and Th2-associated cytokines as well as Th1-associated responses.

To determine if MIS416 treatment reduced the percentage of IL-17 or IFN-γ-producing cells, we compared the number of IL-17-positive and IFN-γ-positive cells in the spleens of MIS416-treated and untreated, immunized mice to untreated, unimmunized mice (i.e. control) at day 8 and day 15 time points. As anticipated, MIS416 treatment led to a reduction in the percentage of IL-17-producing splenocytes compared to untreated immunized mice (Figure 3j). However, MIS416 did not alter the percentage of splenocytes producing IFN-γ in response to MOG stimulation at either 8 or 15 days post immunization (Figure 3l) despite having a significant effect on IFN-γ production at day 15. Subset analysis at day 15 revealed that the IL-17-producing cells were primarily CD4 T cells while an equal percentage of CD4 and CD8 T cells produced IFN-γ (Figure 3k and m). Taken together, these findings indicate that MIS416 significantly suppressed the systemic but not local Th17 and Th1 responses.

### MIS416 Induced a Sustained Expansion of CD4 T Cells in Immunized as Well as Unimmunized Mice

**Figure 4:**
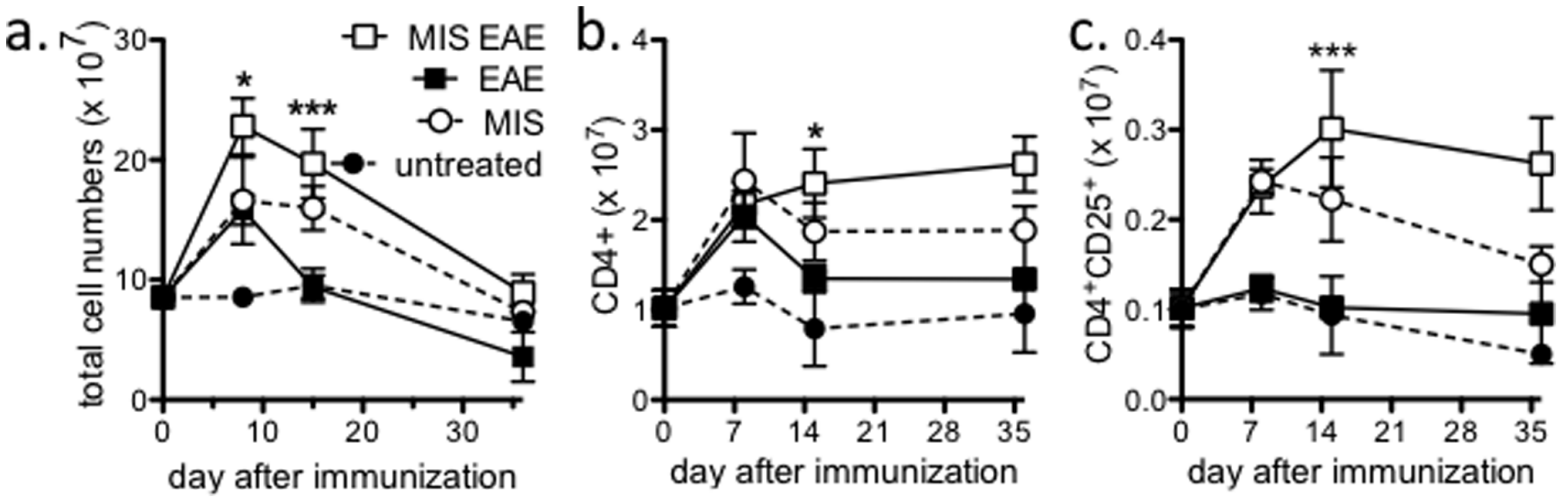
MIS416 treatment induced an expansion in the total number of splenic CD4+ T cells. C57BL/6 mice were immunized to induce EAE (see Methods) and treated weekly by i.v. administration of 100 µg/mouse MIS416 starting on the day of immunization. Splenocytes were isolated at 8, 15, and 36 days post-immunization and assessed by flow cytometric analysis. Shown are the total splenocyte numbers (**a**), total CD4^+^ cells (**b**), and total CD4^+^CD25^+^ cells (**c**) from 2 experiments (n = 3-10/timepoint). * p<0.05 and *** p<0.001 EAE compared to MIS EAE by 2-way ANOVA with Bonferroni’s multiple comparison post-test.

Since MIS416 had such targeted effects on systemic immune responses, we determined whether MIS416 treatment affected the splenic CD4 compartment of immunized and unimmunized animals. While immunization alone induced a transient increase in total splenocyte numbers, MIS416 administration caused a more sustained increase in splenocyte numbers in both immunized and unimmunized mice (Figure 4a). However, by 35 days post immunization the total splenocyte numbers from all groups returned to normal levels (Figure 4a). In contrast to the splenocytes, there was no significant difference in the total number of lymph node cells between any of the groups (Figure S1a). Flow cytometric analysis did not reveal any significant alteration in the percentage of splenic CD4 T cells (Figure S1b) or lymph node CD4 T cells (data not shown) in any of the groups. However, the increase in total splenic cell numbers induced by MIS416 treatment resulted in a significant increase in the total number of CD4 T cells in MIS416-treated, immunized and unimmunized animals (Figure 4b). In addition to the increase in CD4 T cells, the number and percentage of splenic CD25^+^CD4^+^ T cells was also significantly increased in MIS416-treated immunized and unimmunized mice (Figure 4c and Figure S1c). These CD25^+^CD4^+^ T cells were FoxP3+ (Figure S1d) suggesting that MIS416 treatment may also drive a sustained expansion in splenic CD25^+^CD4^+^ regulatory T cells (Tregs) in addition to CD4 T cells (Figure 4b and c). Overall, given the similarity in the percentage of splenic CD4 T cells between all groups, this finding suggests that the differences in antigen-specific cytokine responses observed in the splenocyte cultures were not attributable to a reduced number of CD4 T cells.

### Administration of MIS416 Altered the Splenic Myeloid Compartment

**Figure 5:**
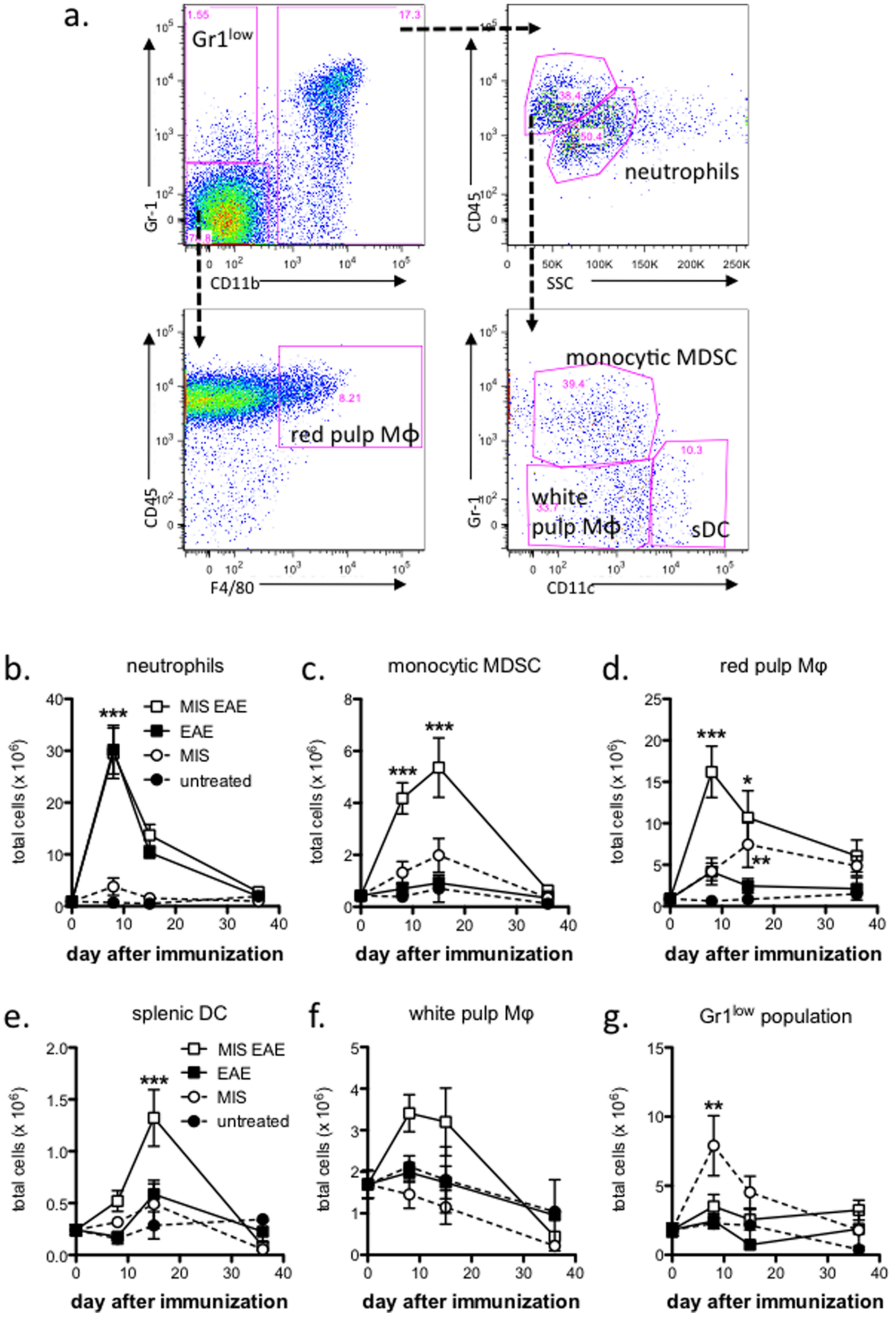
The splenic myeloid compartment was significantly altered by MIS416 administration and EAE immunization. C57BL/6 mice were immunized to induce EAE (see Methods) and treated weekly by i.v. administration of 100 µg/mouse MIS416 starting on the day of immunization, and splenocytes were isolated at 8, 15, and 36 days post-immunization and assessed by flow cytometric analysis. (**a**) Myeloid population gating strategy using CD11b, Gr-1, CD45, side scatter (SSC), CD11c, and F4/80 expression. Shown are representative plots of live, single cells from an MIS416-treated, immunized mouse. (**b–g**) MIS416 treatment led to an expansion in the MDSC (**c**) and red pulp macrophage (MΦ) (**d**) populations in both immunized and unimmunized mice (p<0.001 by 2-way ANOVA; MIS EAE compared to EAE and p<0.05; MIS compared to untreated) while MIS416-treated, immunized mice had significantly expanded splenic DC (**e**) but not white pulp MΦ (**f**) (p<0.05 by 2-way ANOVA; MIS EAE compared to EAE). In contrast, no significant differences were found between EAE treatment groups in neutrophil numbers (**b**). The Gr1^low^ population (**g**) was significantly enhanced in the MIS416-treated, unimmunized animals compared to the untreated animals (p<0.01 by 2-way ANOVA; MIS compared to untreated), and modestly altered in MIS416-treated immunized animals (p<0.05 by 2-way ANOVA; MIS EAE compared to EAE). Shown are the means and SEM of values from individual mice (n = 6-10 per group) from 2 experiments. * p<0.05, ** p<0.01, and *** p<0.001 by Bonferroni’s multiple comparison post-test; MIS EAE compared to EAE or MIS compared to untreated.

Since MIS416 specifically targets myeloid cells [12] and myeloid-derived cytokines are known regulators of Th subset differentiation and maintenance, we investigated how MIS416 treatment altered the splenic myeloid subpopulations. A recent study by Greifenberg *et al*. [16] characterized 6 myeloid subpopulations based upon their CD11b and Gr-1 expression. Using a similar strategy, we identified 6 distinct splenic subpopulations with characteristics similar to those of Greifenberg *et al*. (Figure 5a and Table 2), and these subpopulations were identified as neutrophils, white pulp or marginal zone macrophages (i.e. CD11b^+^Gr-1^−^F4/80^−^), red pulp macrophages (i.e. CD11b^−^Gr-1^−^F4/80^+^), splenic dendritic cells (DC), monocytic myeloid derived suppressor cells (MDSC), and a Gr1^low^ population similar to the mouse type I IFN-producing cells (mIPC) described by Asselin-Paturel *et al.* [17] (Table 2).

**Table 2:** Myeloid subset surface marker expression.

Cell type (ref 16)	Gr-1	CD11b	F4/80	CD14	CD11c	Ly6G	Ly6C
MonocyticMDSC	+*^a^*	++	+	+	–	−	++
Splenic DC	−	+	+/−	+/−	++	−	−
White pulp/marginalzone MΦ	−	+	+/−	+/−	−	−	−
Neutrophils	++	++	−	−	−	++	−
Gr1^low^ (mIPC)(ref 17)	+	−	−	−	−	−	++
Red pulp MΦ	−	−	+	+/−	+/−	−	−

*a*: ++, high expression level; +, medium expression level; +/−, low expression level; −, no expression detected.

From this analysis, we observed that immunization induced an increase in the total number of neutrophils independently of treatment (Figure 5b). MIS416 treatment induced a transient expansion of the monocytic MDSC, red pulp macrophages, and splenic DC, which was particularly evident in immunized animals (Figure 5c–e), while no significant differences in white pulp macrophages were observed between any of the groups (Figures 5f). Interestingly in MIS416-treated, unimmunized animals, a Gr1^low^ population was transiently induced and was found to be CD11b^−^, CD11c^−^, F4/80^−^, and Ly6C^+^, which is similar to the type 1 interferon-producing cells (plasmacytoid DC-like) described previously [17] (Figure 5g). When these myeloid subsets were expressed as the proportion of total cells, as opposed to total cell number, only the expansion of MDSC and red pulp macrophages as well as the transient expansion of the Gr1^low^ subpopulation by MIS416 treatment alone remained significant (Figure S2). Thus, overall we conclude that the major MIS416-mediated effects on splenic myeloid populations are in the expansion of monocytic MDSC and red pulp macrophages.

**Figure 6:**
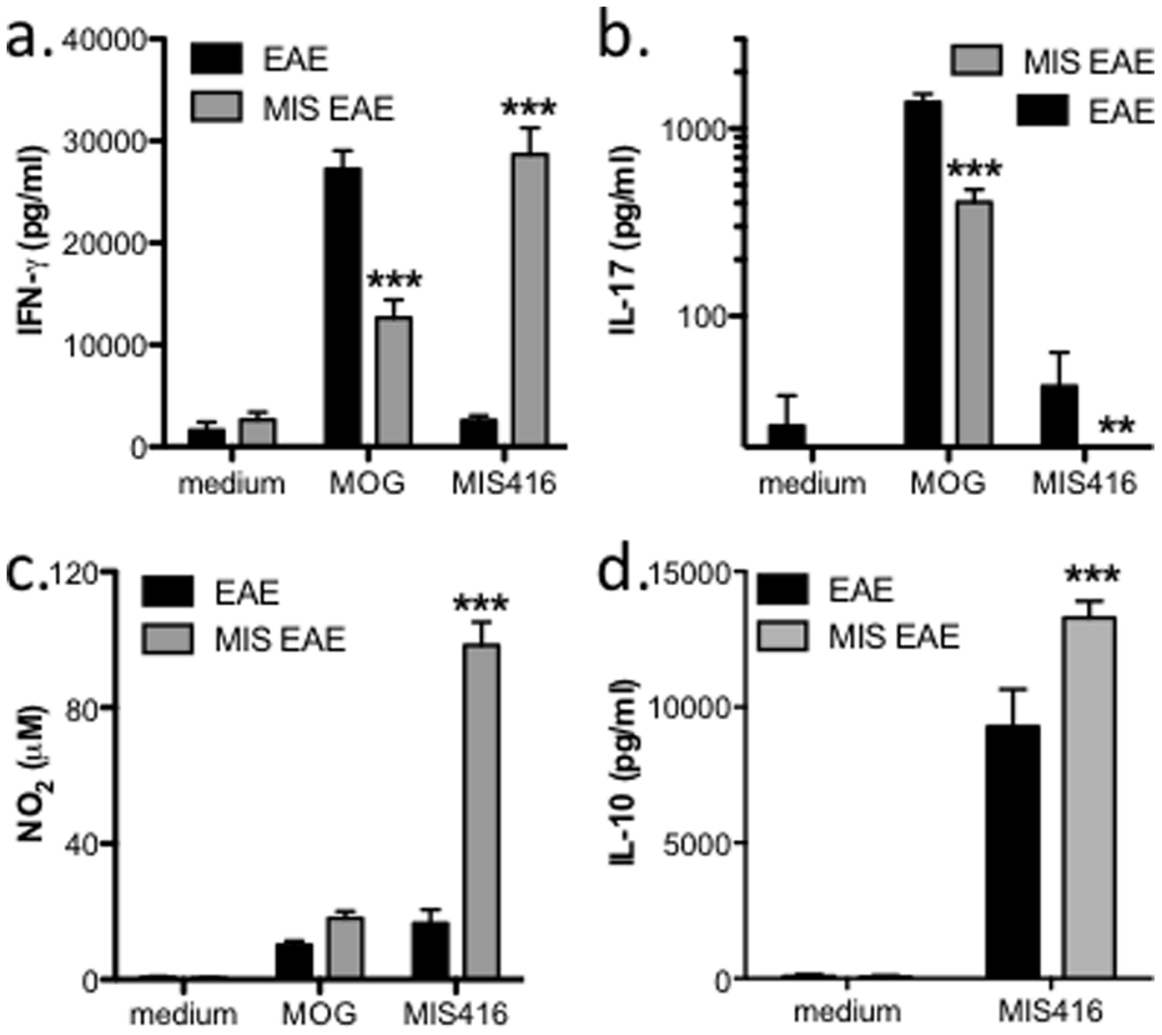
Administration of MIS416 enhanced MIS416-stimulated production of IFN-γ, NO, and IL-10 but not IL-17A by splenocytes from immunized mice. C57BL/6 mice were immunized to induce EAE (see Methods) and treated weekly by i.v. administration of 100 µg/mouse MIS416 starting on the day of immunization. On day 22, splenocytes were isolated from MIS416-treated or untreated, immunized mice and cultured (10^6^ cells/well) with medium alone, MOG peptide (27 µg/ml), or MIS416 (20 µg/ml) for 72 hours. Cytokines in the culture supernatants were assayed by ELISA. Shown are the means and SEM of values from individual mice from 3 **(**n = 15/group; **a–c)** or 2 **(**n = 10/group; **d)** experiments. ** p<0.01 and *** p<0.001 by 2-way ANOVA with Bonferroni’s multiple comparison post-test.

To assess whether MIS416 administration affected the splenic innate MIS416 responses *in vitro*, MIS416-induced cytokine production from splenocytes from MIS416-treated and untreated, immunized mice was compared. In contrast to MOG re-stimulation, MIS416 induced a significantly higher production of IFN-γ and NO by splenocytes from MIS416-treated animals (Figure 6a and c). Furthermore, detectable levels of IL-17 were found in culture supernatants from MIS416-stimulated splenocytes from untreated but not MIS416-treated mice (Figure 6b). Additionally, while only very low levels of MOG-specific IL-10 were detected in culture supernatants from either group, elevated IL-10 levels were produced by MIS416-stimulated splenocytes from MIS416-treated animals (Figure 6d). Similar results are found in MIS416-treated unimmunized mice (data not shown). Given the expansion of the myeloid populations in the spleens of MIS416-treated mice (Figure 5), the increased expression of IFN-γ, NO, and IL-10 is likely to reflect the increase in cell numbers responding to MIS416 *in vitro* as opposed to an increased response per cell. Since MIS416 is administered weekly and forms a depot in the liver as well as spleen for several days post injection (Figure S3), the expansion in cells responding to *in vivo* MIS416 likely establishes an altered immune environment, leading to the altered MOG-specific Th responses.

### Serum IFN-γ Levels were Elevated following MIS416 Treatment and Deficiency in IFN-γ Abolished MIS416-mediated Protection

**Figure 7:**
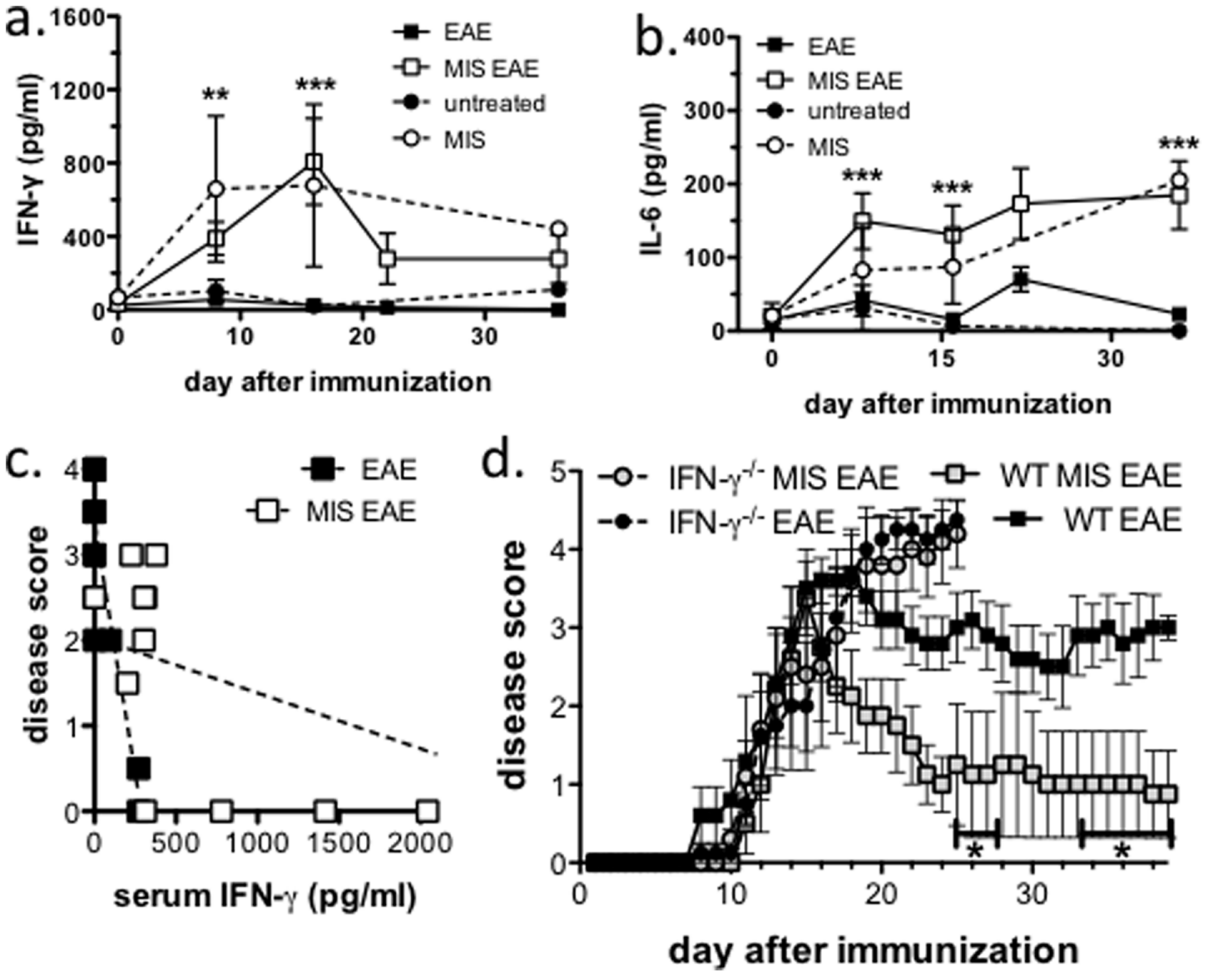
MIS416 treatment elevated serum IFN-γ and IL-6, and deficiency in IFN-γ abrogated the MIS416-mediated reduction in EAE. Wild type (WT; **a–d)** or IFN-γ-deficient (**d**) C57BL/6 mice were immunized to induce EAE (see Methods) and treated with a weekly dose of MIS416 (100 µg/mouse). Elevated levels of serum IFN-γ (**a**) and IL-6 (**b**) were detected after MIS416 treatment in immunized and unimmunized mice. Serum was collected and assayed by CBA, and shown are the means and SEM of values from individual mice (n = 3-26 per group) from 5 experiments. (**a** and **b**) p<0.0001 MIS EAE vs EAE by 2-way ANOVA; (**a**) p<0.05 MIS weekly vs untreated by 2-way ANOVA; and (**b**) p<0.01 MIS weekly vs untreated by 2-way ANOVA. (**c**) Serum IFN-γ levels (n = 12-14 per group) inversely correlated to disease score at day 15. r^2^ = 0.22 with p = NS (MIS EAE) and r^2^ = 0.78 with p<0.0001 (EAE). (**d**) MIS416 treatment did not protect IFN-γ−/− mice from EAE. Mice were weighed and scored daily, and shown are the means and SEM of disease scores. * p<0.05, ** p<0.01, and *** p<0.001 by Bonferroni’s multiple comparison post-test; MIS EAE compared to EAE.

From our studies it is clear that MIS416 treatment causes immediate and systemic effects in both immunized and unimmunized mice. To better understand the systemic effects on immune responses, the levels of serum cytokines were assessed in MIS416-treated and untreated, immunized and unimmunized mice. Only very low levels of IL-1, IL-17A, IL-4, IL-5, IL-10, TNF-α, IL-13, IL-22, IL-21, and IL-27 were detected in the serum; however, both IFN-γ and IL-6 were found at measurable and robust levels (Figure 7a and b). IFN-γ levels were elevated and appeared to peak during the acute phase of EAE, whereas serum IL-6 levels were sustained over the whole time course in mice treated with a weekly low dose of MIS416 (Figure 7b). Importantly, the effects on serum IFN-γ and IL-6 were similar in all MIS416 treated mice, immunized and unimmunized (Figure 7a and b), suggesting that these cytokines are induced solely by MIS416 and not augmented by immunization. Since there was no significant elevation of these cytokines found in untreated, immunized mice when compared to unimmunized mice, this finding provides further evidence that MIS416 induces altered systemic immune responses.

Given the relatively high levels of IFN-γ that were detected in the serum of MIS416-treated mice, we explored whether this cytokine could be involved in disease modulation. Because the serum IFN-γ levels peaked at day 15-post immunization (Figure 7a), we compared serum cytokine levels at this time point and found that mice with the highest serum IFN-γ had the lowest disease score (Figure 7c). Although the linear correlation did not reach significance with the MIS416-treated, immunized mice, it was very significant in untreated, immunized mice although the levels of IFN-γ were substantially lower (Figure 7c; r^2^ = 0.78; p<0.0001). Given the correlation between serum IFN-γ and reduction in disease, we tested whether IFN-γ was involved in disease modulation by comparing the efficacy of MIS416 in wild type C57BL/6 and IFN-γ-deficient mice. As shown in Figure 7d, whereas wild type mice receiving a weekly MIS416 treatment were significantly protected from disease, MIS416 was ineffective at modifying disease in the absence of IFN-γ suggesting that MIS416-mediated protection involves IFN-γ.

**Figure 8:**
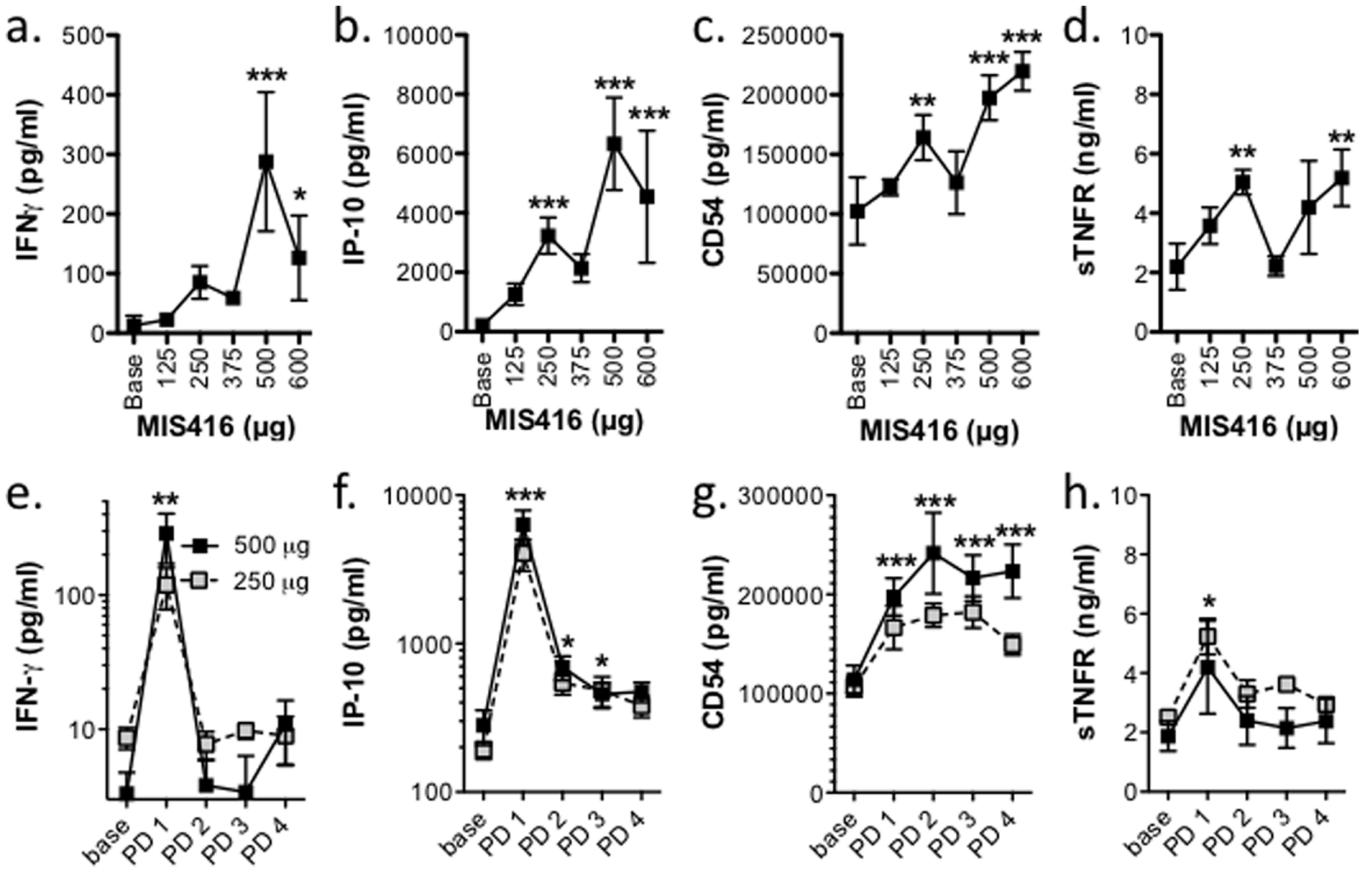
Secondary progressive MS patients treated with MIS416 treatment had elevated serum IFN-γ and IFN-γ-associated proteins. (**a–d**) Patients were treated with 125-600 µg/week MIS416 (n = 3-6 per group) and serum collected at baseline and 24 hours after the first MIS416 treatment. (**e–h**) Patients were treated with 250 or 500 µg/week MIS416 (n = 3–4 per group) for 4 weeks, and serum collected at baseline and 24 hours post each MIS416 dose (PD1, 2, 3, and 4). Cytokines were assessed by CBA, and shown are the means and SEM. * p<0.05, ** p<0.01, and *** p<0.001 by 1-way ANOVA with Dunnett’s post test compared to baseline, and for **e–h** the level of significance is valid for either 250 or 500 µg doses compared to baseline.

Ultimately, these studies were designed to elucidate how MIS416 modifies progressive MS in humans. Thus, to determine whether weekly MIS416 administration caused a similar consistent elevation in serum IFN-γ levels as we observed in mice, we assessed IFN-γ levels in the serum of secondary progressive MS patients enrolled for a phase 1b/2a study designed to confirm the recommended dose of MIS416 and establish its safety profile. Subjects received weekly doses over a 4-week period. Participants were treated with 125–600 µg of MIS416/week, and serum was collected pre-dose 1 (i.e. baseline) and 24 hours post-dose 1, 2, 3, and 4 for pharmacodynamic analysis of MIS416-inducible cytokines and chemokines. MIS416 was found to be well tolerated with the most common adverse events (pyrexia, headache, fatigue, myalgia and muscle stiffness) being transient and expected, due to the immunostimulatory nature of MIS416. There was no worsening of clinical status, and MRI analysis showed no new lesions were induced, nor were any pre-existing lesions enhanced. In agreement with the murine studies, pharmacodynamic analysis of patient serum IFN-γ levels showed that they were increased significantly at higher doses in patients 24 hours post-dose 1 (Figure 8a). In a similar manner, the level of other proteins known to be upregulated by IFN-γ were dose-dependently increased (IP-10 and CD54; Figure 8b and c), and this dose-response relationship was linear (p<0.05; r^2^ = 0.75 for IP-10 and r^2^ = 0.79 for CD54). In contrast, proteins, which are not known to be induced by IFN-γ, were not affected to the same extent as IP-10 or CD54 (sTNFR; Figure 8d) and did not show this linear dose-response relationship (p = 0.25; r^2^ = 0.31). Finally, while the increase in serum IFN-γ was transient, the elevations in IP-10 and CD54 but not sTNFR were maintained for longer (Figure 8e–h). Taken together, these findings demonstrate that MIS416 activates the type II interferon axis and induces an elevation in serum IFN-γ and IFN-γ-inducible proteins in mice and humans, which may be central to the sustained reduction in disease and the alteration in the systemic Th response profile.

## Discussion

One strategy for altering T cell responses is to modify the immune environment through innate cell activation. This strategy is particularly attractive for the treatment of T cell-mediated diseases such as MS. MIS416 is a microparticulate, myeloid-directed, immune response modifier that targets the innate receptors TLR9 and NOD2 and is known to have immunomodulatory properties [12]. We investigated whether this microparticle was able to modify autoimmune responses and thus alter the progression of disease in the mouse model of MS, EAE. Our studies clearly indicate that even a single dose of MIS416 administered either on the day of immunization or after disease onset is effective at reducing disease severity and furthermore, that this effect is augmented by regular administration. MIS416 suppressed systemic antigen-specific Th17, Th1, and Th2-associated cytokines and led to an expansion in specific splenic subpopulations including CD4^+^CD25^+^FoxP3^+^ Tregs, MDSC, and red pulp macrophages. Finally, we showed that MIS416 administration causes an elevation in serum IFN-γ in mice and humans and that this cytokine is involved in the reduction in EAE.

This study is the first to show that a novel microparticle, which simultaneously ligates TLR9 and NOD2 receptors [12], is effective at modifying the course of EAE. The MIS416 microparticle contains MDP repeats, which are naturally occurring NOD2 ligands and are the minimal immune stimulatory component of the bacterial cell wall [12]. Only one other study has investigated the use of monomeric MDP in Freund’s adjuvant to suppress disease in a guinea pig model of EAE [18]. This study simply reported that repeated intraperitoneal administration of MDP in adjuvant suppressed disease expression in 6 of the 8 animals treated. Our work shows significant protection in the mouse model when administered intravenously both on the day of immunization and after disease onset and highlights possible mechanism(s) of action. In contrast to our and Root-Berstein’s studies, Shaw *et al.* found that NOD2 signaling potentiates EAE as mice deficient in NOD2 or RIP2, an essential adapter protein for NOD2 signaling, have significantly reduced disease [3]. Furthermore, a recent study by Satoh *et al*. revealed that RIP2 mRNA is more highly expressed in non-T cell peripheral blood mononuclear cells (PBMC) from MS patients compared to healthy controls [19] suggesting that the NOD2 and NOD1 pathways are inherently more active in MS patients. While the NOD2 mutations, which have been shown to confer susceptibility to Crohn’s disease, were not found to confer susceptibility to MS [20], MS patients heterozygous for a G to A substitution in the rs5743291 allele had enhanced myelin basic protein-specific IL-17 and IL-5 responses suggesting that NOD2 polymorphism may alter Th biasing [21]. Interestingly, we found that administration of MIS416 and thus ligation of NOD2 and TLR9 simultaneously suppressed Th17, Th1, and Th2 responses.

The use of single TLR9 agonists in EAE has been investigated previously but with conflicting results [6], [22], [23]. TLR9 binds bacterially derived DNA but more commonly synthetic CpG sequences are used as agonists. Previous studies have shown that administration of bacterial DNA promotes EAE [6] while several studies show that both actively induced and adoptively transferred EAE is reduced in mice deficient in TLR9 suggesting that signalling through this receptor is involved in the disease process [4], [5]. In contrast, other studies have demonstrated that CpG administration suppresses disease [22], [23] and the disease course is exacerbated in TLR9 deficient animals [7]. Recent evidence also suggests that in MS patients, IFN-β can inhibit TLR9 processing, suggesting an interesting pathway by which IFN-β treatment may be immunomodulatory [24]. Taken together these studies indicate that TLR9 may play both a protective and detrimental role in EAE and MS, which is very dependent upon the context, location, and magnitude of TLR9 ligation.

In our studies we have used MIS416, which contains bacterially derived DNA along with MDP, bound together in the form of a 2 µm microparticle [12] and therefore it is distinct to the soluble MDP, CpG, or bacterial DNA used in previous studies. As a direct result of the simultaneous activation of both TLR9 and NOD2, MIS416 is likely to induce different signalling pathways compared to either agonist alone. Furthermore, the restricted uptake of MIS416 by phagocytic cells results in targeted modulation of the innate wing of the immune system, which contrasts with soluble agonists, which can directly activate a broader range of innate and adaptive immune subsets. Additionally, pharmacokinetic studies have shown that systemically administered MIS416 forms a main depot in the liver with smaller amounts trafficking to the spleen and lymph nodes (Figure S3b–e). This depot is cleared gradually over a period of days (Figure S3a, c–f), which is in contrast to the shorter systemic half-lives and poorer tissue accumulation with soluble, CpG and MDP [25], [26]. The distinct tissue localisation pattern and prolonged half-life of MIS416 compared to soluble agonist counterparts is also likely to influence the dominant outcome from combined and more prolonged NOD2 and TLR9 signaling.

Regarding the effects of concomitant activation of NOD2 and TLR9, Castellaneta *et al.* have reported that NOD2 activation can inhibit TLR9-inducd IFN-α, IL-6, and TNF-α production by plasmacytoid DC (pDC) suggesting that co-activation may induce a more tolerogenic immune state [27]. Our results also suggest that a selective tolerogenic state was achieved by combined TLR9/NOD2 activation as we found reduced disease expression as well as antigen-specific Th17 and Th2 responses. Considering the role of the liver in immune regulation and tolerance induction [28], we believe that the delivery of MIS416 to the liver is key to its immunomodulatory effects. A previous study by Castellaneta *et al.* showed that *in vivo* MDP administration altered the response of liver pDC to CpG suggesting that pDC may be key players in tolerance induction by NOD2/TLR9 activation. It is interesting to note that splenic pDC responses were not similarly affected indicating that different immune pathways may be induced depending upon deposition of these agonists in the spleen compared to the liver. These studies are therefore essential in aligning the contradictory results from previous studies using PRR agonists in the EAE model since one explanation for the contradictory findings may be in the method of delivery and localization of the effects. Thus, our results combined with the findings of Castellaneta *et al.* suggest that targeting the liver may provide the best immunomodulatory effects to allow a systemic toleragenic environment that reduces the CNS inflammation, which characterizes EAE and MS [27], [28].

Our results show that MIS416 has a clear immunomodulatory effect during EAE by suppressing Th17, Th2, and Th1 responses as well as inducing increased serum IFN-γ, which is required for MIS416-mediated protection. IFN-γ has been demonstrated to have a paradoxical role in immune regulation, despite its classical pro-inflammatory actions [29]. Although IFN-γ producing Th1 cells have typically been associated with EAE disease process, a regulatory role for IFN-γ in EAE is also evident. It is well documented EAE is exacerbated in either IFN-γ-deficient and IFN-γ receptor-deficient mice or when IFN-γ is depleted [30], [31], [32]. IFN-γ is also protective in EAE when exogenous IFN-γ is administered or if endogenous IFN-γ production is enhanced [33], [34], [35]. Additionally, it has been demonstrated that certain myelin antigens, which are poorly encephalogenic, inhibit disease expression due to the induction of high levels of IFN-γ [31], [36]. Collectively these studies highlight the potential regulatory role IFN-γ can have on EAE disease expression.

There are many potential ways IFN-γ could induce regulatory processes or protective effects in EAE. IFN-γ can induce macrophages and microglia to secrete T cell anti-proliferative or pro-apoptotic factors, for example through up regulation of nitric oxide synthase-2 or indoleamine dioxygenase production [37], [38]. Sustained levels of IFN-γ can also directly reduce T cell reactivity though altering receptor expression [39]. IFN-γ production may also counter-regulate Th17 responses resulting in a biasing of encephalogenic responses towards Th1 during the earlier time points (i.e. day 8) [40], [41]. Attenuation of tissue destruction can also be induced though IFN-γ mediated down regulation of MMP production [42], [43]. It is therefore likely that that multiple IFN-γ induced regulatory mechanisms may be associated with MIS416 disease modifying effects in EAE.

The clinical utility of exogenous IFN-γ to treat MS was explored in a pilot study conducted in the late 1980’s in relapse-remitting MS (RRMS) patients, and it was found that in patients receiving intravenous IFN-γ, the disease appeared to be exacerbated [44]. However as Sriram *et al* suggest, it is plausible that the apparent worsening of disease was due to pseudo-relapses as a consequence of IFN-γ enhancement of pre-existing MS-associated inflammatory activity in these RRMS patients [45]. This possibility is underscored by the fact that there was no new clinical activity detected during the 6–12 months follow-up period as measured by neurological disability scores [44]. In contrast to RRMS, systemic exogenous IFN-γ may be more effective for the progressive forms of disease, where the disease-promoting axis is no longer T cell dependent but has shifted towards an innate myeloid/microglial mediated inflammation [46].

The failure of exogenous IFN-γ therapy for RRMS may also be directly related to the general limitations of systemic cytokine monotherapy such as lack of cell targeting and induction of “off target” effects as well as the inability to establish the appropriate cellular crosstalk that is likely to play an important role in reshaping immune responses. The ability to induce endogenous innate cell-derived IFN-γ may therefore be central to achieving clinical benefit. The fact that MIS416 induces endogenous IFN-γ in mice and humans in conjunction with overall innate immune cell activation represents a clear point of difference compared to exogenous IFN-γ monotherapy. Preliminary findings indicate that innate NK/NKT cells are the primary source of IFN-γ following indirect MIS416 stimulation as opposed to T cells/monocytes (data not shown). Given that MIS416 forms a depot in the liver, a rich source of these cells, it is plausible that liver-resident NK/NKT are the source of IFN-γ when MIS416 is administered systemically and further work is directed at confirming this hypothesis.

As well as modulating cytokine production, MIS416 also had significant effects on immune cell populations in the spleen including splenic myeloid subsets as well as Tregs. Tregs have a significant role in driving disease resolution in EAE, and depletion of Tregs exacerbates EAE while transfer of Tregs can suppress disease [47]. Furthermore, decreased numbers of Tregs have been reported in MS patients and treatment with the MS drug, glatiramer acetate, has been shown to correlate to an increase in Treg numbers and function [48]. Thus, the significant expansion of splenic CD4^+^CD25^+^FoxP3^+^ Tregs by MIS416 administration in both unimmunized and immunized mice indicates that this expansion, if in concert with enhanced Treg function, may in part explain the immunomodulatory effects of MIS416 and this possibility merits further investigation.

The MIS416-mediated changes to the splenic myeloid subsets suggest that these populations, like the Treg, may contribute to the immunomodulatory effects of MIS416. In particular, we found clear changes induced by MIS416 or immunization alone although we have not yet explored the functional consequences of these changes. Arora *et al.* have described the expansion and suppressive activity of a regulatory myeloid population (CD11b^+^Gr-1^int^F4/80^+^) in the lungs after intratracheal exposure to LPS [49] suggesting that PRR agonists can induce suppressive activity. This regulatory myeloid population remained in the local tissue and was found to be able to suppress ovalbumin-induced allergic inflammation [49]. A similar population of myeloid-derived suppressor cells (CD11b^+^Ly6G^−^F4/80^+^) has also been described in the blood, and this monocyte population was shown to suppress T cell proliferation and EAE when transferred into immunized mice [50]. Thus, an expansion of these cells in the spleen could provide another pathway by which MIS416 may reduce peripheral, autoreactive immune responses and suppress CNS inflammation in EAE.

## Conclusions

This study is the first to show that a microparticle containing TLR9 and NOD2 agonists induces a sustained immunomodulatory effect when administered at weekly intervals and these effects on the immune system are sufficient to suppress the CNS inflammation and paralysis that characterizes EAE. We provide evidence that IFN-γ is involved in this protection but suggest that an expansion in splenic Tregs and regulatory myeloid cells may also contribute to the MIS416-mediated effects. The recent phase 1b/2a clinical trial in secondary progressive MS patients has confirmed safety and tolerability on a repeat dosing schedule and provides evidence that the effects we observe in MIS416-treated mice occur in humans as well. While previous compassionate use in secondary progressive patients has provided strong anecdotal evidence for its efficacy, our studies conclusively show that MIS416 is protective in our experimental model and begin to dissect the mechanism by which MIS416 works. While the use of PRR agonists to treat several inflammatory diseases such as allergy, inflammatory bowel disease, and systemic lupus erythematosus is under development, this study is the first to assess the use of a dual PRR agonist for MS [51].

## Supporting Information

Figure S1 **MIS416 treatment did not alter the total number of LN cells (a) nor the % of splenic CD4 T cells (b) but increased the % of splenic CD25^+^CD4^+^ cells (c).** (d) The CD4^+^CD25^+^ cells, which were expanded by MIS416 treatment, were FoxP3^+^. (e). Flow cytometric analysis of intracellular IFN-γ and IL-17 production.
(DOC)

Figure S2 **The splenic myeloid compartment (% live cells) was significantly altered by MIS416 administration and EAE immunization.**
(DOC)

Figure S3 **Calculated half-life of MIS416-FITC in the blood, liver and spleen following i.v. delivery of 250 µg MIS416-FITC and Supporting Materials and Methods.**
(DOC)

Information S1 **Trial Protocol (MIS416-201).**
(PDF)

Information S2 **CONSORT checklist.**
(PDF)
