# Supplementary material for: Targeting Innate Receptors with MIS416 Reshapes Th Responses and Suppresses CNS Disease in a Mouse Model of Multiple Sclerosis
Source: PLoS One. 2014 Jan 31;9(1):e87712. doi: 10.1371/journal.pone.0087712 (PMC3909208; doi:10.1371/journal.pone.0087712)
Supplement: Figure S1 — MIS416 treatment did not alter the total number of LN cells (a) nor the % of splenic CD4 T cells (b) but increased the % of splenic CD25+CD4+ cells (c). (d) The CD4+CD25+ cells, which were expanded by MIS416 treatment, were FoxP3+. (e). Flow cytometric analysis of intracellular IFN-γ and IL-17 production. (DOC) [file pone.0087712.s001.doc]

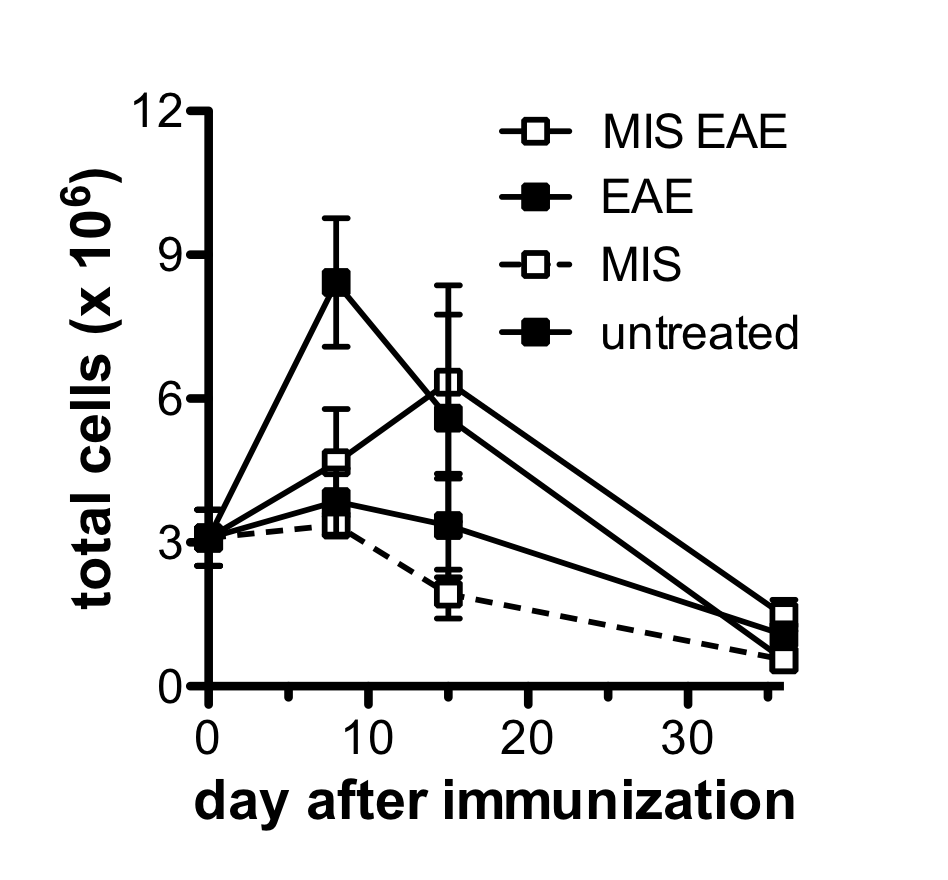

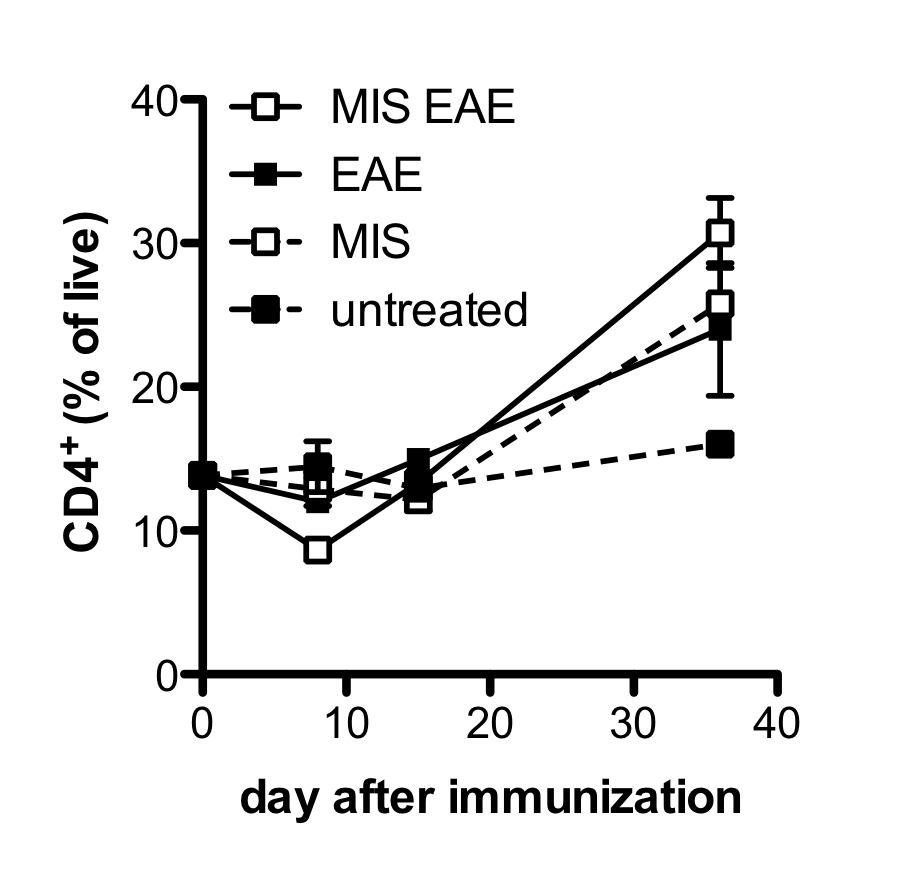

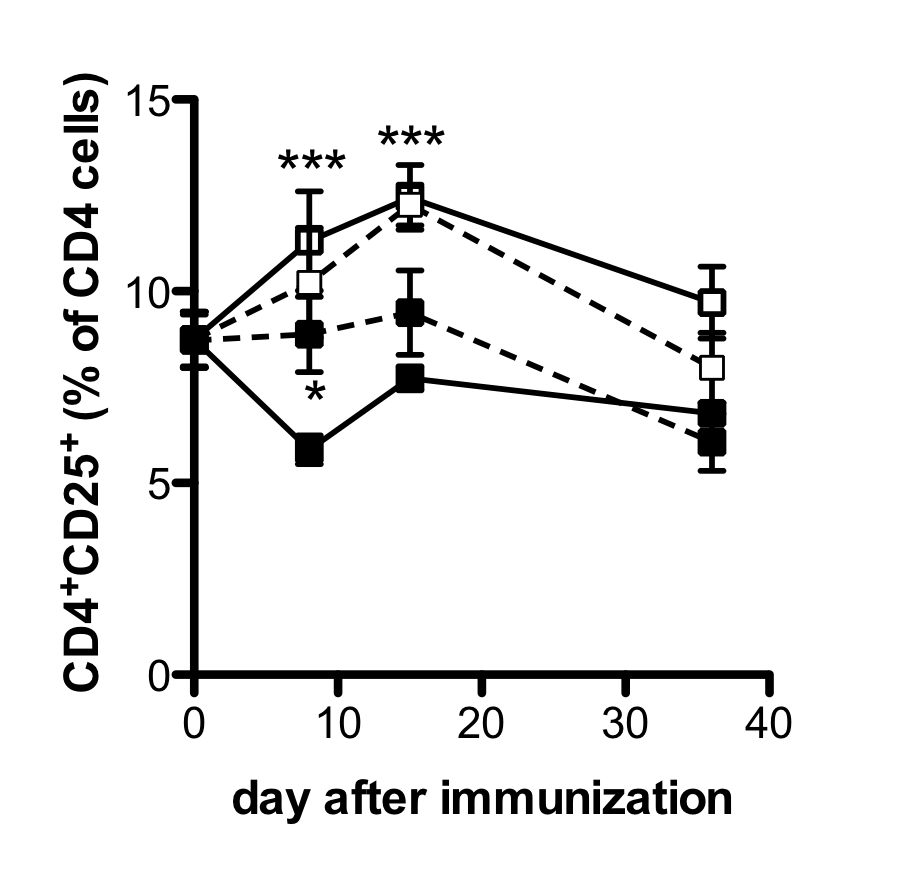

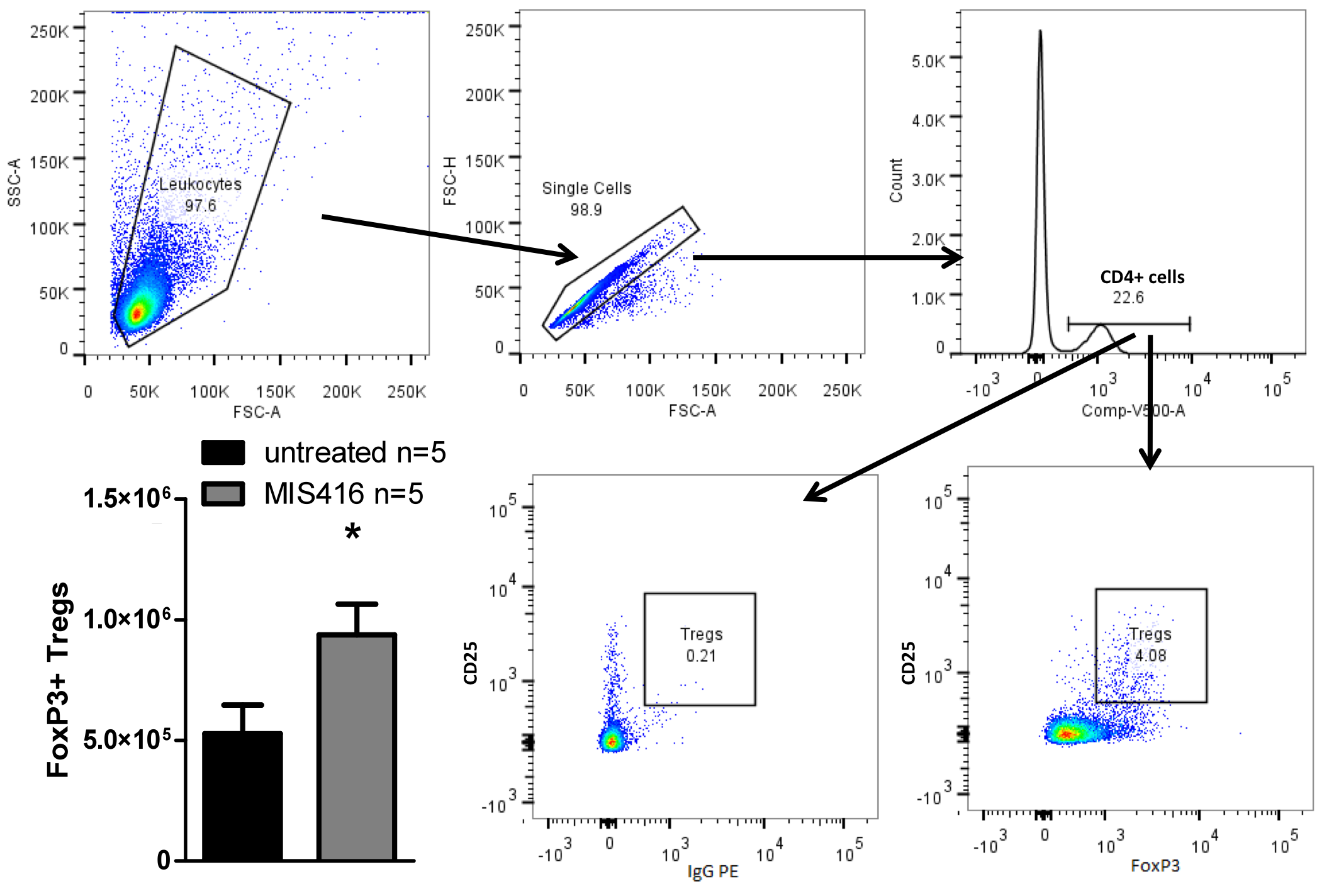

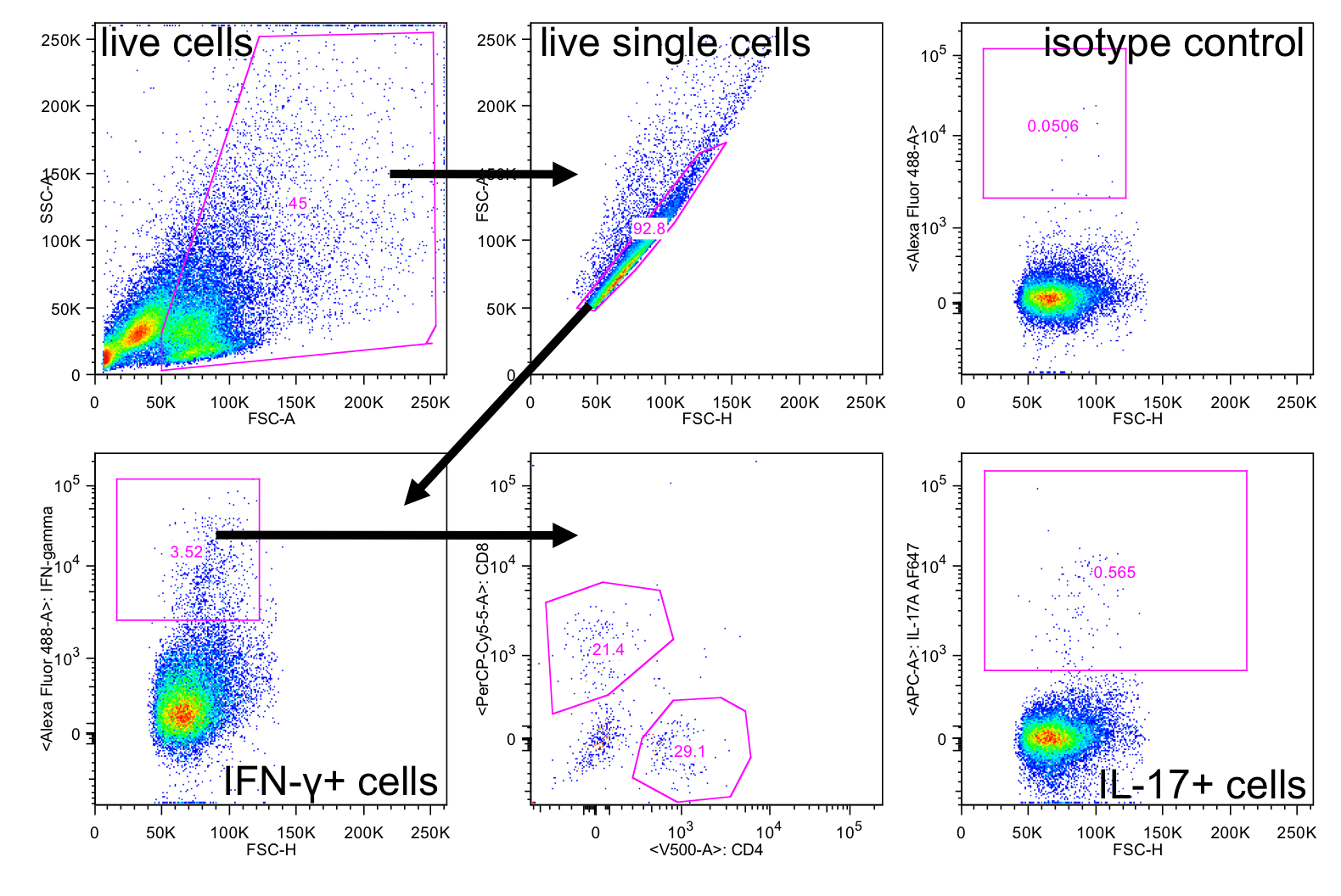
Supplementary Figure 1: MIS416 treatment did not alter the total number of LN cells (a) nor the % of splenic CD4 T cells (b) but increased the % of splenic CD25^+^CD4^+^ cells (c). (d) The CD4^+^CD25^+^ cells, which were expanded by MIS416 treatment, were FoxP3^+^. (e). Flow cytometric analysis of intracellular IFN-γ and IL-17 production. Mice were immunized to induce EAE (see Methods) and treated weekly by i.v. administration of 100 μg/mouse MIS416 starting on the day of immunization and LN cells and splenocytes were isolated at 8, 15, and 36 days post immunization and assessed by flow cyotmeric analysis. Shown are the means and SEM of the total number of LN cells (a), CD4^+^ cells as % of live cells **(b)** and CD4^+^CD25^+^ cells as % of CD4 cells **(c)** from individual mice (n = 6-10 per group) from 2 experiments. (d) Shown are the gating strategy confirming FoxP3 expression in CD4^+^CD25^+^ cells and the means and SEM of the total number of FoxP3^+^CD4^+^CD25^+^ cells in MIS or untreated, unimmunized mice (n = 5 per group) at day 15. (c) ***p < 0.001 by 2-way ANOVA with Bonferroni’s multiple comparison post test; MIS EAE compared to EAE and (d) *p < 0.05 by Student’s t test; MIS compared to untreated. (e) Shown is the gating strategy quantifying splenocyte IFN-γ and IL-17 production in response to MOG and identification of cytokine^+^CD4^+^ or CD8^+^ cells.

**a.**

**b.**

**c.**

**d.**

isotype control

FoxP3

**e.**
