# Supplementary material for: Targeting Innate Receptors with MIS416 Reshapes Th Responses and Suppresses CNS Disease in a Mouse Model of Multiple Sclerosis
Source: PLoS One. 2014 Jan 31;9(1):e87712. doi: 10.1371/journal.pone.0087712 (PMC3909208; doi:10.1371/journal.pone.0087712)
Supplement: Figure S2 — The splenic myeloid compartment (% live cells) was significantly altered by MIS416 administration and EAE immunization. (DOC) [file pone.0087712.s002.doc]

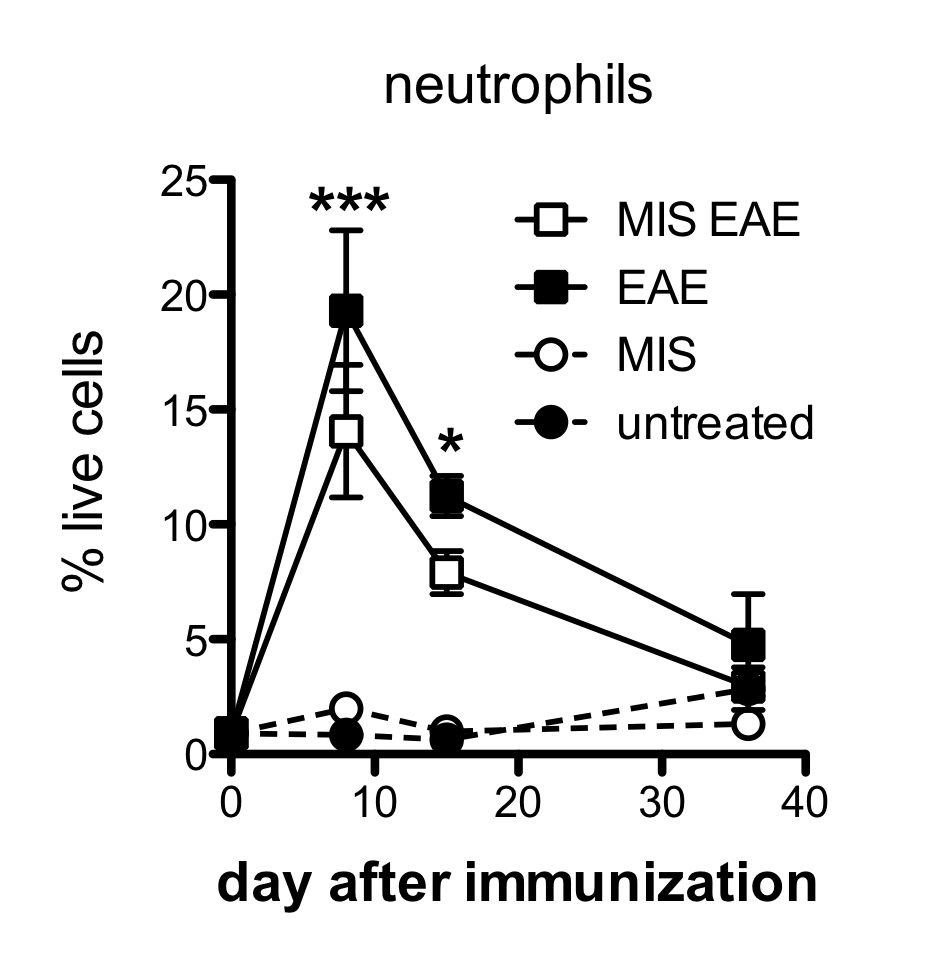

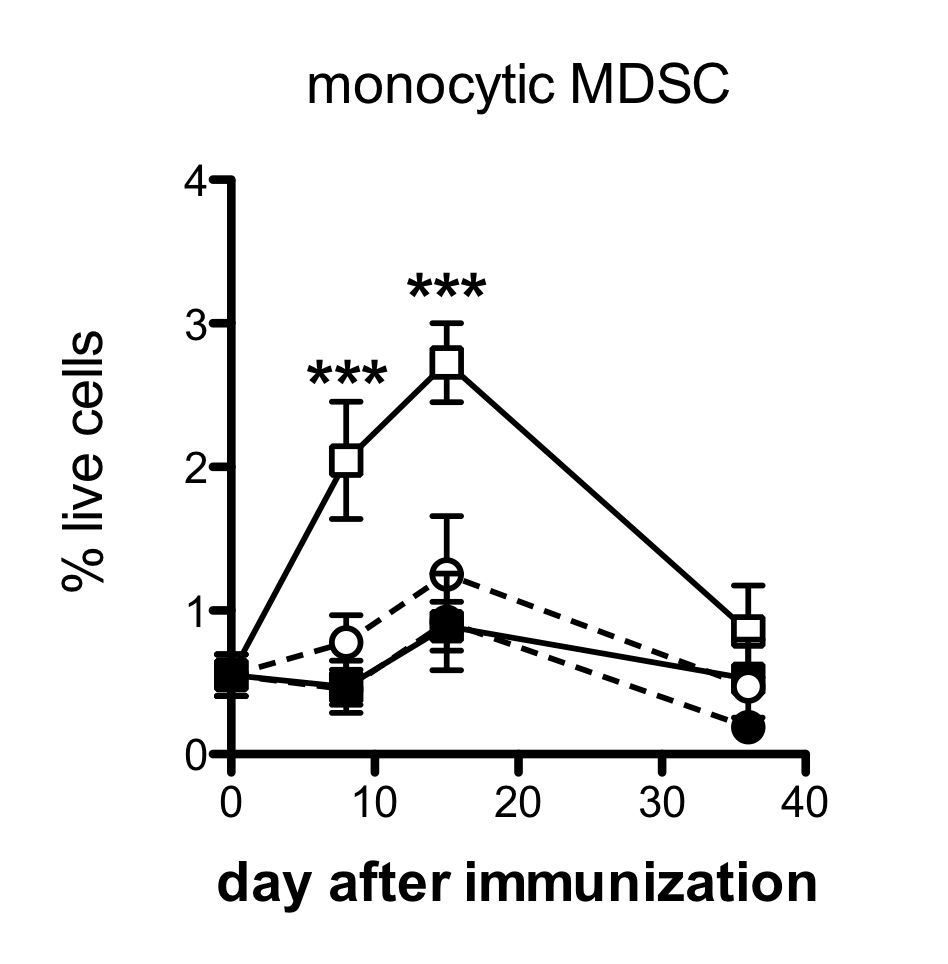

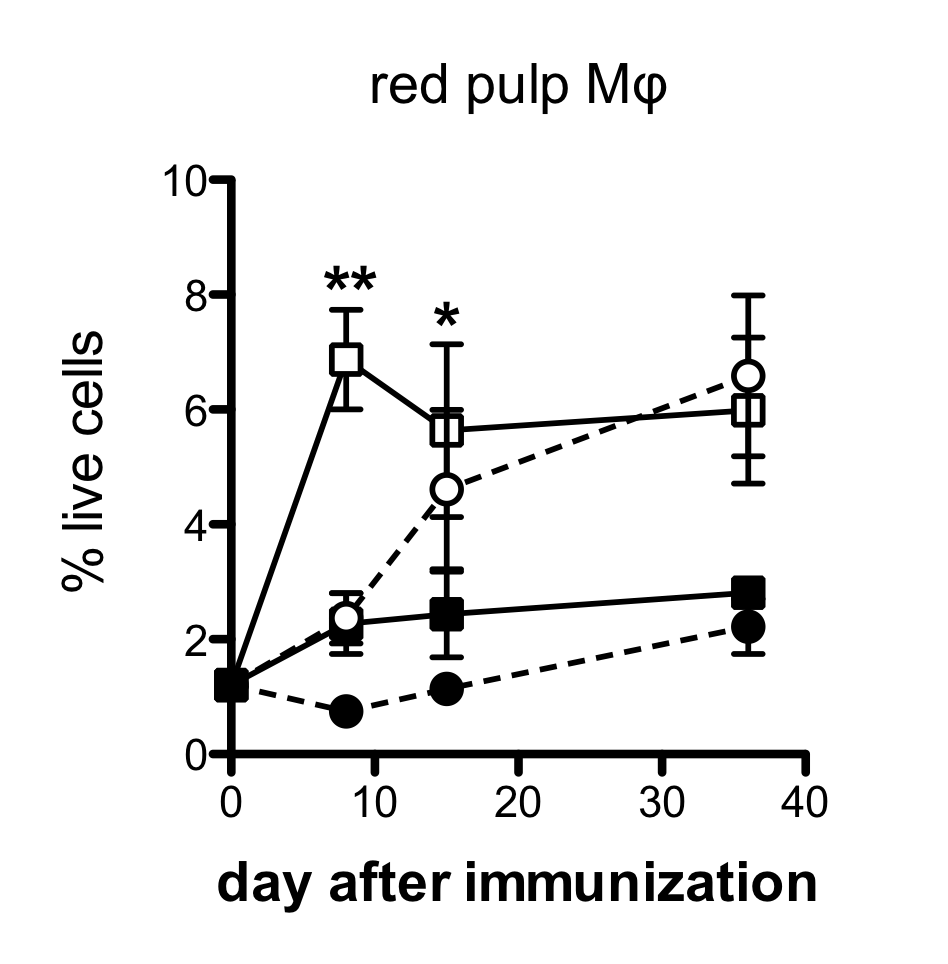

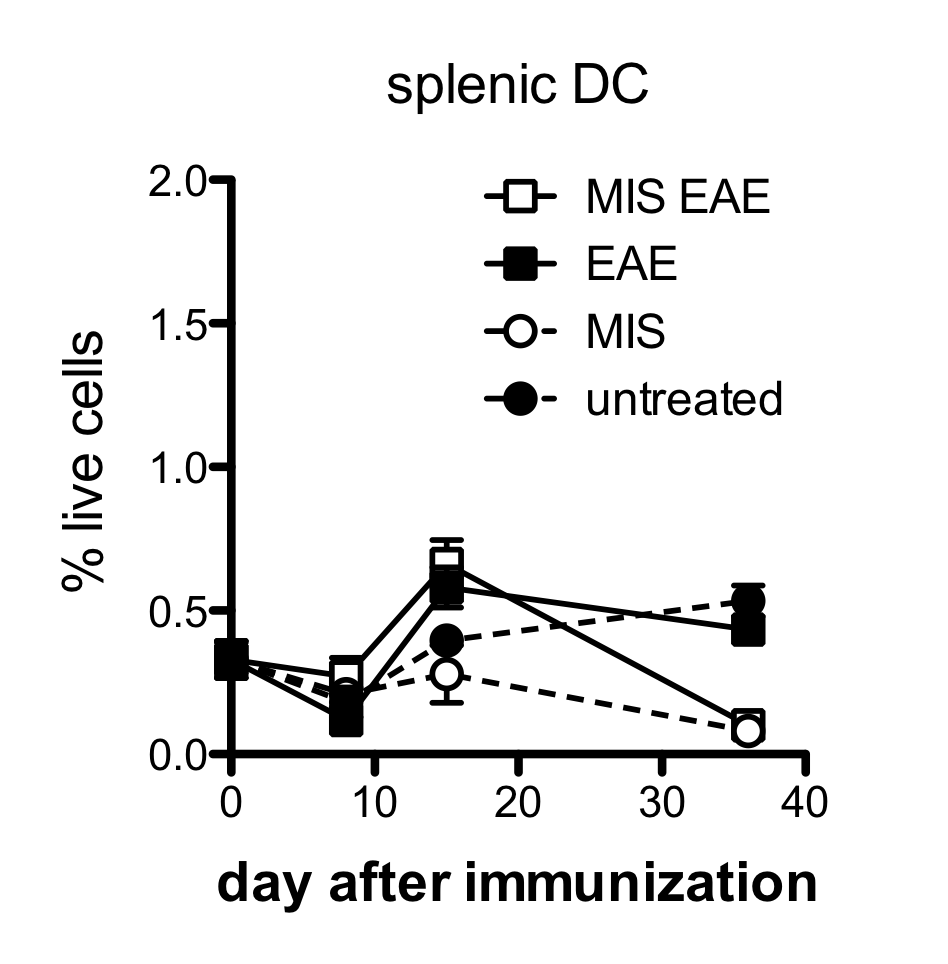

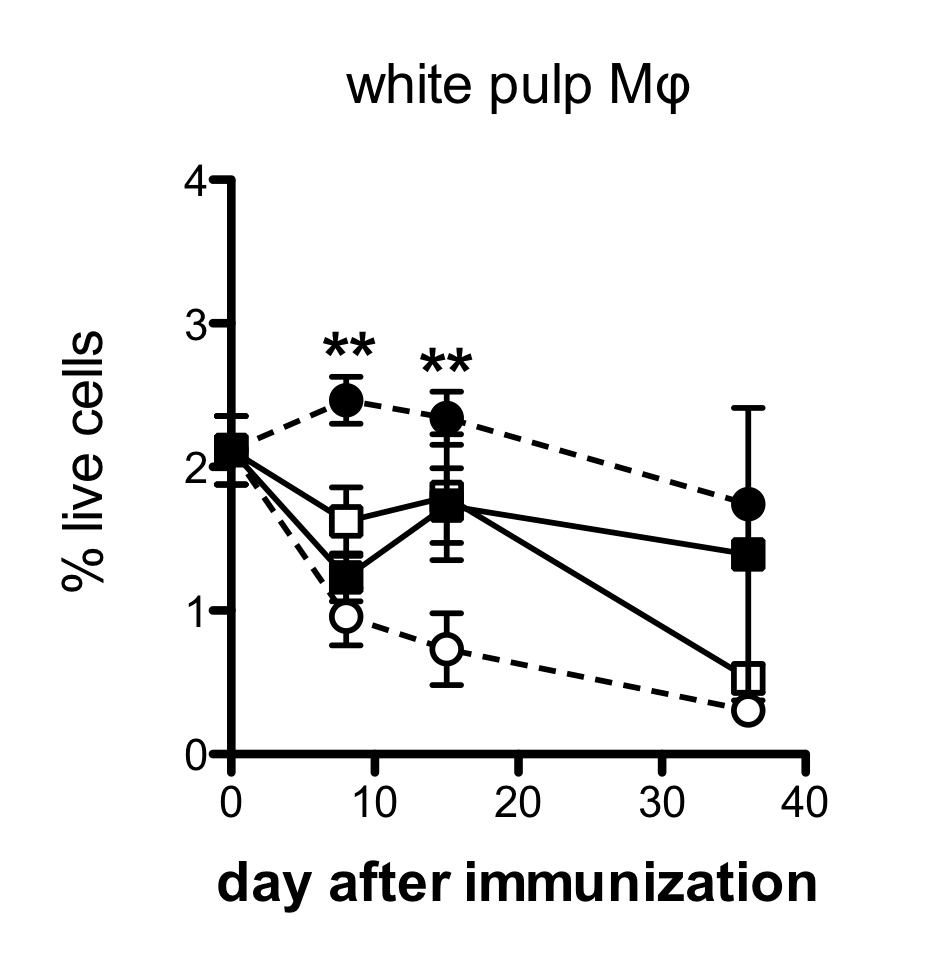

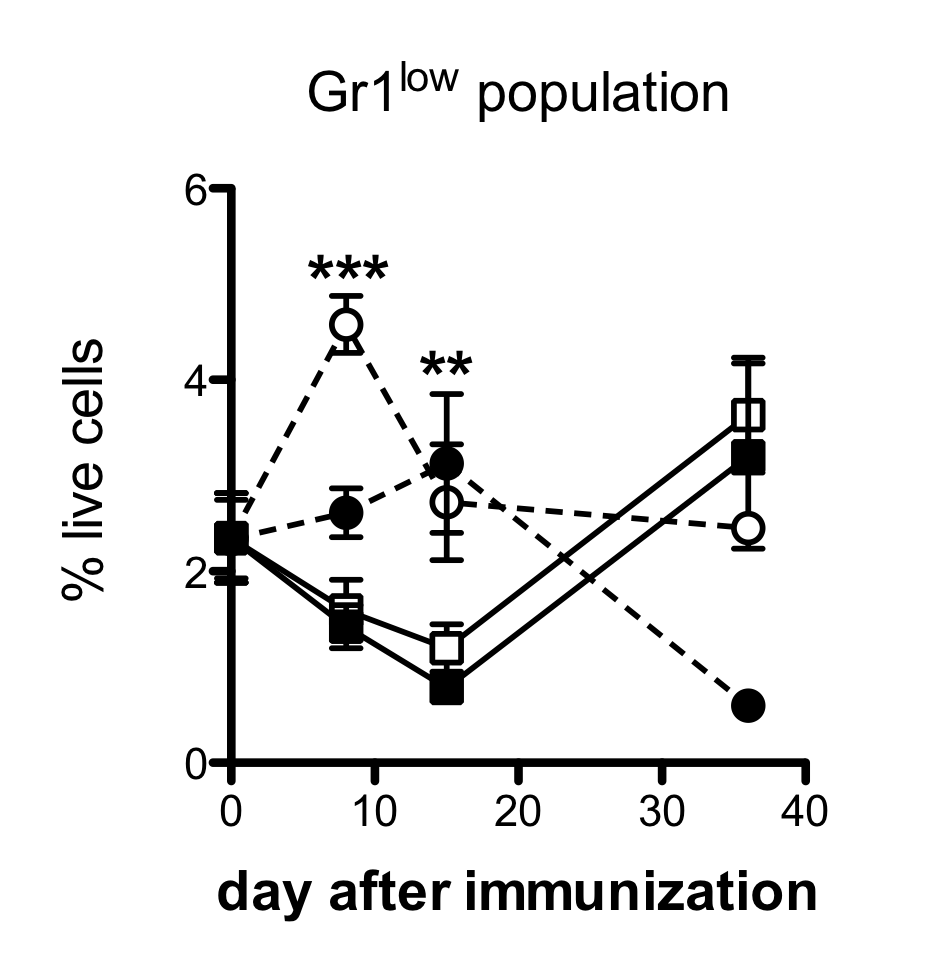


**a.**

**b.**

**c.**

**e.**

**f.**

Supplementary Supplementary Figure 2: The splenic myeloid compartment (% live cells) was significantly altered by MIS416 administration and EAE immunization. Mice were immunized to induce EAE (see Methods) and treated weekly by i.v. administration of 100 μg/mouse MIS416 starting on the day of immunization. Splenocytes were isolated at 8, 15, and 36 days post immunization and assessed by flow cytometric analysis. Day 0 values are from untreated, unimmunized mice (n = 8). Shown are the means and SEM of the % live cells for all 6 subpopulations from individual mice (n = 6-10 per group) from 2 experiments. *** p < 0.001, ** p < 0.01, and * p < 0.05 by 2-way ANOVA with Bonferroni’s multiple comparison test (a) MIS EAE compared to MIS and EAE compared to untreated; (b and c) MIS EAE compared to EAE; (e) untreated compared to MIS; and (f) MIS compared to MIS EAE on day 8 and untreated compared to EAE on day 15.

**d.**
