# Supplementary material for: Targeting Innate Receptors with MIS416 Reshapes Th Responses and Suppresses CNS Disease in a Mouse Model of Multiple Sclerosis
Source: PLoS One. 2014 Jan 31;9(1):e87712. doi: 10.1371/journal.pone.0087712 (PMC3909208; doi:10.1371/journal.pone.0087712)
Supplement: Figure S3 — Calculated half-life of MIS416-FITC in the blood, liver and spleen following i.v. delivery of 250 µg MIS416-FITC and Supporting Materials and Methods. (DOC) [file pone.0087712.s003.doc]

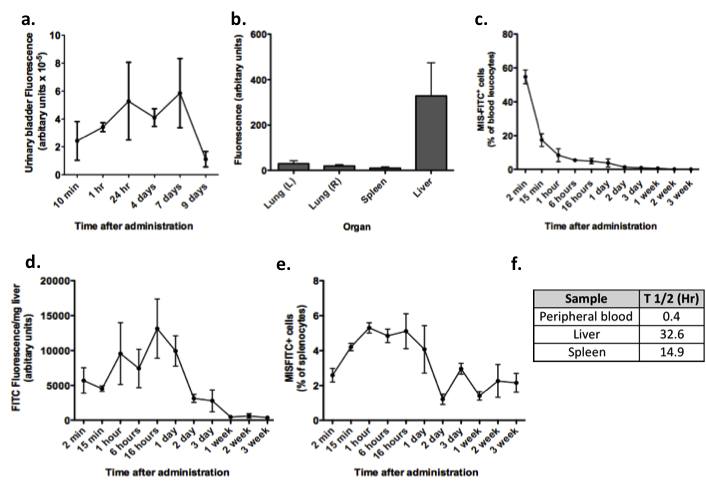


Supplementary Figure 3: Calculated half-life of MIS416-FITC in the blood, liver and spleen following i.v. delivery of 250 μg MIS416-FITC (a) Whole body imaging revealed detectable urinary bladder associated fluorescence over the 9-day period, indicative of ongoing MIS416-FITC excretion. *Ex vivo* analysis on day 11 showed substantial fluorescence remaining associated with the liver in particular, suggesting the liver is a major depot site for i.v. MIS416-FITC (b). Clearance of MIS416-FITC from the peripheral blood circulation (c) coincided with accumulation of MIS416-FITC in the liver (d) and spleen (e). The overall half-life for a single i.v. bolus of MIS416-FITC is shown (f).

Supplementary Materials and Methods for Figure 3:

*MIS416 pharmacokinetic and tissue distribution analysis*

Preliminary tissue accumulation of MIS416 was determined under contract by MRI (Anne Arbour, Michigan). In summary, 250 μg of FITC-labeled MIS416 was administered in the tail vein of female NCR (nu/nu) mice (n=3). Whole body *In vivo* fluorescence imaging was performed using an IVIS 50 optical imaging system

(Caliper Life Sciences, Hopkinton, MA). The animals were imaged at 10 minutes, 1 hour, 24 hours, then day 4, 7 and 9 with termination of the study and *ex vivo* imaging on Day 11. *Ex vivo* imaging was performed on excised tissues using the same optical imaging system.

For detailed pharmacokinetic analysis of MIS416 depots in the liver and spleen as well as removal from the blood circulation, 500 μg of FITC-MIS416 was administered via the tail vein to C57/Bl6 mice (n=5). Peripheral blood, liver and spleens were harvested at 2 min, 15 min, 60 min, 6 hr, 16 hr, 1 day, 2 day, 3 day, 1 week, 2 week and 3 weeks after a single dose. Spleens and peripheral blood were used for analysis of MIS416-FITC+ cells by flow cytometry: Spleens were processed to achieve a single celled suspension and nucleated cells were stained using 10 μg/mL Hoechst 33428 DNA specific dye for 1 hr at 37^o^C. Propidium iodide (PI; 1 μg/mL final) was added to identify non-viable cells and live cells were gated based on DNA content and PI exclusion. For peripheral blood analysis 100 μL of whole heparinized blood was labeled with hoechst and propidium iodide in the same way as for splenocytes. FITC-positive cells were expressed as a % of total nucleated cells.

Livers were snap frozen in liquid nitrogen and retained until completion of the study. FITC was extracted from liver following homogenisation and digestion of the tissue in 40 K U/mL of proteinase K (Sigma) in tissue digestion buffer (100 mM Tris 5 mM EDTA, 200 mM NaCl, 5 mM CaCl2) conducted at 56^o^C in the dark, overnight. Samples were cleared using the top speed of a microfuge and supernatants analyzed alongside known amount of digested MIS416-FITC for the amount of soluble FITC contained within the supernatant using a fluorescent plate reader and FITC excitation and emission filters. The amount of MIS416-FITC in the liver was calculated using the standard curve and expressed as μg/mg tissue. Half-lives were determined by regression analysis of the semi-logarithmic % MIS416-FITC^+^ cells or fluorescence units versus time data using Excel plug-in PK function (these functions were designed and developed by Joel Usansky and Atul Desai of the Department of Pharmacokinetics and Drug Metabolism at Allergan, Irvine CA).
