# Supplementary material for: Targeting Innate Receptors with MIS416 Reshapes Th Responses and Suppresses CNS Disease in a Mouse Model of Multiple Sclerosis
Source: PLoS One. 2014 Jan 31;9(1):e87712. doi: 10.1371/journal.pone.0087712 (PMC3909208; doi:10.1371/journal.pone.0087712)
Supplement: Information S1 — Trial Protocol (MIS416-201). (PDF) [file pone.0087712.s004.pdf]

innate therapeutics

MIS416 -201

A Phase 2, open-label, dose-escalation study evaluating the safety, tolerability, and pharmacodynamics of intravenously administered MIS416 in patients with chronic progressive multiple sclerosis

Version 5

29 July 2011

(Including amendments 1, 2, and 3)

Confidentiality Statement

The information contained in this document, especially unpublished data, is the property of Innate Therapeutics Ltd and is provided in confidence to the investigator, his/her staff and an applicable Health and Disability Ethics Committee for review. It is understood that this information will not be disclosed to others without written authorization from Innate Therapeutics Ltd, except to the extent necessary to obtain informed consent from those persons to whom the drug may be administered.

Author Signatures:

Dr Alison Luckey BM Dip Pharm Med MFPM
Medical Director, Primorus Clinical Trials Ltd
Co-Principal Investigator

Signature: Alison Luckey Date: 04 AUG 2011

Dr Michael H Silverman MD FACP
President, BioStrategics Consulting Ltd
Medical Monitor (by his authorised agent).

Signature: Date: 29 July 2011

Simon Wilkinson
CEO
Innate Therapeutics Ltd

Signature: Date: *29 July 2011*

INVESTIGATOR SIGNATURE SHEET

I have read the attached protocol and agree that it contains all the necessary details for performing the study.

I will provide copies of the protocol and of the preclinical information on the test article, which was furnished to me by the sponsor, to all members of the study team responsible to me who participate in the study. I will discuss this material with them to assure that they are fully informed regarding the test article and the conduct of the study.

Once the protocol has been approved by the ethics committee, I will not modify this protocol without obtaining the prior approval of the sponsor and of the ethics committee.

I will submit the protocol modifications and/or any informed consent modifications to the sponsor and the ethics committee, and approval will be obtained before any modifications are implemented.

I understand the protocol and will work according to it, the principles of Good Clinical Practice (current ICH guidelines), and the Declaration of Helsinki (1964) including all amendments up to and including the Scotland revision (2008).

Co-Principal Investigator's Signature	04 AUG 2011. Date
Co-Principal Investigator's Signature	08/AUG/2011 Date

GLOSSARY OF ABBREVIATIONS

AE	adverse event
ALT	alanine aminotransaminase
ANC	absolute neutrophil count
APPT	Activated Partial Thromboplastin Time
AST	aspartate aminotransaminase
BCG	Bacille Calmette-Guerin
BMI	body mass index
C3, C4	complement factors 3 and 4
CS	clinical status
CADASIL	cerebral autosomal dominant arteriopathy with subcortical infarcts and leukoencephalopathy
CAF	CD8 anti-viral factor
CD40	costimulatory protein
CpG	synthetic single-stranded immunostimulatory deoxyribonucleic acid sequence
CPMS	Chronic Progressive Multiple Sclerosis
CRA	clinical research associate
CRF	case report form
CRP	c-reactive protein
DC	Dose-confirmation
DE	Dose-escalation
DLT	dose limiting toxicity
DNA	deoxyribonucleic acid
EAE	experimental autoimmune encephalomyelitis
ECG	electrocardiogram
eCRF	electronic case report form
EDSS	Extended Disability Status Scale
FAS	Fas Ligand
FSH	follicle stimulating hormone
FSS	Fatigue Severity Scale
GLP	Good Laboratory Practice
GM-CSF	granulocyte macrophage colony stimulating factor
HIV	human immunodeficiency virus
ICH	International Conference on Harmonization
IFN-α	interferon – alpha
IFN-γ	interferon – gamma
IL	interleukin
ITT	Intention to treat
IV	intravenous
LPS	lipopolysaccharide

LT	leucotriene
MedDRA	medical dictionary for regulatory activities
MDP	muramyl dipeptide
MOG	myelin oligodendrocyte glycoprotein
MRI	magnetic resonance imaging
mRNA	messenger ribonucleic acid
MS	multiple sclerosis
MSFC	multiple sclerosis functional composite
MTD	maximum tolerated dose
NOAEL	no observed adverse effect level
NOD-2	nucleotide oligomerization domain-2
NK	natural killer
NYHA	New York Heart Association
PBMC	peripheral blood mononuclear cell
PD	pharmacodynamic
pDC	plasmocytoid dendritic cell
PPMS	primary progressive multiple sclerosis
PPR	pathogen molecular recognition receptor
RNAi	RNA interference
RORyt	orphan nuclear receptor transcription factor
RP2D	recommended Phase 2 dose
SE	safety evaluable
SF-36	Short Form Health Survey – 36 questions
siRNA	short interfering RNAs
SPMS	secondary progressive multiple sclerosis
Sponsor	Innate Therapeutics Limited
SRT	Safety Review Team
Tbet	T helper 1-specific T box transcription factor
TLR9	Toll-like receptor 9
TNF	tumour necrosis factor
WHODrug	World Health Organisation Drug dictionary

SYNOPSIS

**Name of Sponsor/Company:** Innate Therapeutics Limited
**Name of Product:** MIS416
**Title of Study:** MIS416-2101: A Phase 2, open-label, dose-escalation study evaluating the safety, tolerability, and pharmacodynamics of intravenously administered MIS416 in patients with chronic progressive multiple sclerosis.
**Objectives:**<br>The primary objectives of this study are:<br>1. To determine the safety and tolerability, dose-limiting toxicities (DLTs), maximum tolerated dose (MTD), and recommended Phase 2 dose (RP2D) of intravenously (IV) administered MIS416 weekly in patients with chronic progressive multiple sclerosis (CPMS); and<br>2. To assess the pharmacodynamic (PD) effects of MIS416, including effects on serum cytokine levels and peripheral blood mononuclear cell (PBMC) -associated cytokine/chemokine expression patterns.<br>The secondary objectives of this study are:<br>1. To document any changes in MS clinical status occurring during the 12-week MIS416 dosing period in the dose-confirmation phase, as determined by the Multiple Sclerosis Functional Composite (MSFC), Fatigue Severity Scale (FSS), Short Form Health Survey (SF-36), and Expanded Disability Status Scale (EDSS); the frequency of clinical relapses; and signs of clinical activity on serial cranial MRI scans; and<br>**2.** To evaluate, in exploratory fashion, any correlations between clinical, radiological and PD outcomes.
**Study Design:** This is a single center, open-label, non-randomized, dose-escalation study, to be conducted in two phases: a dose-escalation (DE) phase, to evaluate the safety, tolerability, MTD, and PD of MIS416 administered IV once weekly for 4 doses; and a dose-confirmation (DC) phase, which will be a cohort expansion at or below the MTD (i.e., the RP2D) of MIS416, dosed once weekly for up to 12 doses. Subjects will be treated with a weekly IV dose of MIS416 in 28-day cycles: 1 cycle in the DE phase, and up to 3 cycles in the DC phase. The initial dose of MIS416 will be 125 µg/week. Subjects will be evaluated and dosed weekly each cycle in each phase. Subjects will return for a follow-up visit 7 days after completion of the last dose of study drug.

Name of Sponsor/Company: Innate Therapeutics Limited

Name of Product: MIS416

Number of Subjects Planned: The total number of subjects to be enrolled in the study is dependent upon the observed safety profile, which will determine the number of subjects per dose level, as well as the number of dose escalations required to achieve the MTD. It is anticipated that approximately 27-33 subjects will be enrolled in this study, with approximately 12-18 subjects in the dose-escalation phase and up to a further 15 subjects in the dose-confirmation phase. The study will be conducted at a single site.

Diagnosis and Main Criteria for Inclusion:

Inclusion Criteria

1. At least 18 years of age.
2. Diagnosis of MS, by the McDonald criteria[17] (see Appendix 1).
3. Chronic progressive MS (CPMS), defined as either primary progressive MS (PPMS) or secondary progressive MS (SPMS), per the criteria of the National Multiple Sclerosis Society (USA) Advisory Committee on Clinical Trials of New Agents in Multiple Sclerosis[18]. [NOTE: In the dose-confirmation phase, only subjects with SPMS may be enrolled].
4. MS is clinically active with worsening clinical status within the past 2 years.
5. Expanded Disability Status Scale (EDSS) of 2.5 to 7.0 at Screening.
6. The following laboratory values must be documented within 3 days prior to initiation of study drug:
    - Absolute neutrophil count (ANC) ≥ 1 x 109/L
    - Platelet count ≥ 100 x 109/L
    - Serum creatinine ≤ 1.5 mg/dL
    - AST (SGOT) and ALT (SGPT) ≤ 2 × upper limit of normal.
7. Provide written informed consent to participate.
8. Willing to comply with scheduled visits, treatment plans, laboratory assessments, and other study-related procedures.

Exclusion Criteria

1. Relapsing-remitting MS or progressive-relapsing MS
2. Any immunomodulatory drug therapy or immunosuppressive therapy within the previous six months, or vaccine or systemic corticosteroids within the previous 60 days, prior to initiation of study drug.
3. Exposure to other experimental treatments currently under investigation in MS

Name of Sponsor/Company: Innate Therapeutics Limited

Name of Product: MIS416

clinical trials, including alemtuzamab, rituximab, fingolimod, and cladribine.

4. A diagnosis or history of collagen vascular disease (including Sjögren's syndrome and systemic lupus erythematosus), anticardiolipin antibody syndrome, cerebral autosomal dominant arteriopathy with subcortical infarcts and leukoencephalopathy (CADASIL), sarcoidosis, vasculitis, Bechet's syndrome and/or Lyme disease.
5. History of alcohol or drug abuse (with the exception of cannabinoids) within 2 years prior to initiation of study drug.
6. Inability to refrain from smoking for the duration of the inpatient stay.
7. Major surgery or radiation therapy within 28 days prior to initiation of study drug.
8. Active infection requiring antibiotics within 2 weeks prior to initiation of study drug.
9. Active malignancy within 2 years of entry, with the exception of basal cell carcinoma and squamous cell carcinoma of the skin
10. Uncontrolled congestive heart failure (New York Heart Association Classification 3 or 4 [Appendix 2]), angina, myocardial infarction, cerebrovascular accident, coronary/peripheral artery bypass graft surgery, or transient ischemic attack within 3 months prior to initiation of study drug.
11. Symptomatic cardiac dysrhythmias requiring treatment, or persistent prolongation of the QTcF (Frederica) interval to > 450 msec for males or > 470 msec for females.
12. In the Dose-Confirmation phase only, screening echocardiogram which shows abnormalities judged by the investigator to be clinically significant, including but not limited to a left ventricular ejection fraction below the testing facility's lower limit of normal.
13. Pregnant or lactating female.
14. Women of childbearing potential, unless surgically sterile for at least 3 months (i.e., tubal ligation), postmenopausal for at least 12 months (and follicle-stimulating hormone [FSH] > 20 IU/mL), or unless they agree to use effective, dual contraceptive methods (i.e., oral, injectable, or barrier method with male partner using a condom) while on study drug.
15. Men of childbearing potential who partner with a woman of childbearing potential, unless they agree to use effective, dual contraceptive methods (i.e., a condom, with female partner using oral, injectable, or barrier method) while on study drug and for three months following discontinuation of study drug.
16. Known human immunodeficiency virus or acquired immunodeficiency syndrome-related illness.

Name of Sponsor/Company: Innate Therapeutics Limited

Name of Product: MIS416

17. Serological evidence of Hepatitis B, C or HIV infection.
18. Any severe, acute or chronic medical or psychiatric condition or laboratory abnormality that may increase the risk associated with study participation or study drug administration, may interfere with the informed consent process and/or with compliance with the requirements of the study, or may interfere with the interpretation of study results and, in the investigator's opinion, would make the patient inappropriate for entry into this study.
19. Previous exposure to MIS416.

Test Product, Dose, and Mode of Administration: MIS416 is an investigational immunomodulatory agent comprised of muramyl dipeptide and single stranded bacterial DNA fragments which are cross linked together to form an insoluble microparticle. MIS416 is formulated to 0.2 mg/mL, packaged into 7 mL glass vials and heat treated at 100°C. MIS416 will be administered IV over 10 minutes beginning on Day 1 of the study, and thereafter weekly during the 28-day cycle; 4 doses will therefore constitute a cycle.

The Cohort 1 dose will be 125 µg/week. Subsequent cohorts in the DE phase will receive, respectively, doses of up to 250, 500, and 1000 µg/week. The decision to proceed to dosing the next cohort will be made by the Safety Review Team (see below).

Dose-Escalation Phase (1 cycle of treatment only): In the DE phase, the dose of MIS416 will be escalated in successive cohorts of 3 subjects per dose level. The first subject of each cohort will be dosed at least 48 hours prior to the rest of the cohort. If none of the 3 subjects at a dose level experience DLT (see below), then 3 new subjects may be entered at the next higher dose level. If 1 of 3 subjects experiences a DLT, 3 more subjects will be started at the same dose level (total of 6 subjects in cohort). If no additional subjects experience DLT at that dose, the next higher dose level cohort will be initiated. If 2 or more subjects experience DLT, no further subjects will be started at that dose, MTD will be declared, and dose-escalation will cease (subject to confirmation by the Safety Review Team).

In the event that a single subject in a cohort experiences a DLT, the dose escalation increments will be reduced by at least 50% in the subsequent cohort. Any subject experiencing a DLT during the DE phase will be withdrawn from the trial.

The MTD is defined as the highest dose level at which <2 of 6 subjects develop DLT. New dose levels may begin accrual only if all subjects at the current dose level have been observed for a minimum of 7 days from the 4th dose of the Cycle 1. The RP2D will be the MTD; if no MTD is reached, RP2D will be determined based upon evaluation of all clinical and PD data.

**Name of Sponsor/Company:** Innate Therapeutics Limited
**Name of Product:** MIS416

A Safety Review Team comprised of the Co-Principal Investigators, the Sponsor's medical monitor, and an independent clinical expert, will be empanelled. At the conclusion of each DE cohort, the Safety Review Team will meet by teleconference to determine:

- whether the next cohort should be enrolled;
- if so, the increment of dose escalation; or
- if not, that MTD has been achieved.

The SRT will further determine the progression to, dose, and size of the RP2D cohort; oversee subject safety during the DC phase; and recommend DC dose modifications as needed.

Dose-Confirmation Phase (up to 3 cycles of treatment): The DC phase will not begin until the Sponsor has submitted to the Ethics Committee and regulatory authority the requisite sub-chronic nonclinical safety data, and received approval to proceed to 3 cycles of dosing. In the DC phase, up to 15 subjects will be recruited at the RP2D. The Safety Team will have the responsibility of reviewing safety data from the DC phase of the trial and making recommendations regarding dose modifications. If a DC phase subject withdraws from dosing within the first 2 cycles for reasons unrelated to drug toxicity, the subject may be replaced at the discretion of the Safety Review Team.

Dose-Limiting Toxicity (DE phase only): A DLT is a clinically significant adverse event (AE) or laboratory abnormality assessed by a Co-Principal Investigator as at least possibly related to the study drug and unrelated to disease progression, concurrent illness, or concomitant medications occurring during the DE phase of the trial. Toxicity will be graded and recorded according to Toxicity Grading Scale for Healthy Adult and Adolescent Volunteers Enrolled in Preventive Vaccine Clinical Trials, modified to include a Systemic line item for Rigors/Chills (Appendix 3).

A DLT is defined as:

- Grade 3 or 4 adverse event, except for fatigue and fever and/or rigors/chills.
- Grade 4, or an increase above baseline by 2 or more grades, fatigue.
- Grade 4 fever and/or rigors/chills.

Intra-Subject Dose Reduction (DC phase only): An individual subject's dose may be reduced to the next lower cohort dose level (ie, as determined from the DE phase), at the Co-Principal Investigators' discretion and with the approval of the Sponsor, following a DLT or other clinically significant toxicity. The dose may not be reduced below 125 µg/week.

**Name of Sponsor/Company:** Innate Therapeutics Limited
**Name of Product:** MIS416
**Duration of Treatment:** Each treatment cycle will be 28 days of weekly dosing (ie, 4 doses). This trial will entail 1 cycle of treatment in the DE phase and up to 3 cycles of treatment in the DC phase.
**Criteria for Evaluation:**<br>**Safety:** The primary endpoint is to determine the safety profile and MTD of MIS416. Assessments will include characterization of DLTs; characterization of the type, incidence, severity, timing, seriousness, and relationship to treatment of AEs; effects on vital signs and laboratory parameters; changes in electrocardiograms (ECGs) and ophthalmologic examinations; and safety MRI assessments.<br>**Pharmacodynamics:** Pharmacodynamic testing will include some or all of the following serum and cellular assays during the dose-escalation phase: Serum IFNγ, IL-17, IL-6, TNFα and GM-CSF levels; PBMC expression of mRNA encoding IL-12p40, IL-12p35, IL-23p19, LTα, LTβ, IFNγ, IL-17, GM-CSF, RORγt, and Tbet; PBMC production of IFNγ, IL-17, and GM-CSF in response to stimulation with anti-CD3 and anti-CD28 ex vivo; PBMC production of IL-12p40, IL-12p70, IL-23, TNFα and IL-6 in response to stimulation with LPS/IFNγ ex vivo. Depending upon findings during the dose-escalation phase, a more selective or comprehensive set of these assays will be performed during the dose-confirmation phase.<br>**Pharmacogenomic Analysis:** Following specific written consent of the subject, at least one aliquot of PBMC will be sent to Dr David Booth, University of New South Wales, for genomic analysis.<br>**Pharmacokinetics:** Pharmacokinetic analysis is not applicable in this study due to the nature of MIS416 and its mode of action.<br>**Clinical Status:** Subjects will be assessed by EDSS and Multiple Sclerosis Functional Composite (MSFC)[19]; MRIs for clinical status will be performed at the end of the DC phase, according to a protocol to be specified in the Radiology Operations Manual.
**Statistical Analysis:**<br>**Safety Analyses:** Safety data analysis will be conducted on all subjects receiving at least one dose of MIS416. Analyses will consist of data summaries for clinical, biological, and PD parameters, and for AEs. The number and percentage of subjects experiencing one or more AEs will be summarized by dose level group, relationship to study drug, and severity. AEs will be coded using MedDRA terminology. Laboratory parameters will be summarized using descriptive statistics, by post-dosing shifts relative to baseline, and data listings of clinically significant abnormalities. Vital signs, ECG, and ophthalmologic examination data will be summarized by changes from baseline values at each dose

**Name of Sponsor/Company:** Innate Therapeutics Limited
**Name of Product:** MIS416
level using descriptive statistics.<br>**Pharmacodynamic Analyses:** Summary statistics will be computed and the absolute and percent change from baseline will be calculated for each subsequent measurement. Summary statistics will be computed for each collection time point, as well as by dose.<br>**Clinical Status Analyses (DC phase only):** Clinical status will be tabulated using EDSS, MSFC, FSS, and SF-36; MRI findings; and by the frequency of self-reported as well as clinically confirmed clinical exacerbations.

CONTENTS

1. BACKGROUND AND RATIONALE 17
1.1 Scientific Background 17
1.2 Study Drug 18
1.3 Mode of action of MIS416 18
1.4 Pre-Clinical Experience 24
1.4.1 Acute Dose Studies 26
1.4.2 Repeat Dose Studies 26
1.4.3 4 Week Repeat Dose Studies 27
1.4.4 26 Week Repeat Dose Study 28
1.4.5 Animal Toxicology Studies Conclusions 29
1.5 Clinical Experience 29

2. RATIONALE FOR STUDY DESIGN 32
2.1 Developmental Context 32
2.2 Rationale Dose-Escalation Phase 33
2.3 Rationale Dose-Confirmation Phase 33
2.4 Selection of Starting Dose (DE Phase) 33

3. OBJECTIVES OF THE STUDY 35

4. STUDY DESIGN 36
4.1 Overview of Study Design 36
4.2 Number of Subjects Planned 36
4.3 Dose Escalation Phase (1 cycle only) 36
4.4 Dose Limiting Toxicity (DE phase only) 37
4.5 Dose Confirmation Phase (up to total of 3 cycles) 37
4.6 Intra-Subject Dose Reduction (DC phase only) 37
4.7 Antipyretic Pre-Medication (DC phase only) 38
4.8 Safety Review Team 38

5. STUDY POPULATION 38
5.1 Target population 38
5.2 Inclusion Criteria 38
5.2 Exclusion Criteria 39
5.4 Concomitant Medication 40
5.5 Restrictions 41

6. SCHEDULE OF ASSESSMENTS AND PROCEDURES 41
6.1 Screening (Days -28 to -2) 41

6.2 Treatment Period (Cycle 1) – Day 1 41
6.2.1 DE Phase Subjects....41
6.2.2 DC Phase Subjects 42
6.3 Treatment Period (Cycle 1) – Days 8, 15 and 22 (+ 1) – All Subjects....42
6.4 Treatment Period (Cycles 2 – 3) – Days 1, 8, 15 and 22 (+ 1) – DC Phase Subjects Only ..43
6.5 Treatment Period -End of Dosing (Day 29) or Early Discharge- (7 +1 days following last dose of drug)....44
6.5.1 DE Subjects....44
6.5.2 DC Subjects....44
6.6 Follow-up (15 Days Post-End of Dosing) – All Subjects 45

7. SAFETY ASSESSMENTS....52
7.1 Adverse events....52
7.2 History and Physical and Neurological Examinations 52
7.3 Ophthalmologic Examinations 52
7.4 Safety Laboratory Tests 52
7.5 Vital Signs 53
7.6 ECG and Echocardiogram....53
7.7 Body Weight, Height and BMI....54
8. Pharmacodynamic (PD) & immune parameter sampling....54
9. Pharmacogenomic Analysis....54

10. CLINICAL STATUS ASSESSMENTS 55
10.1 Multiple Sclerosis Functional Composite (MSFC)....55
10.2 Fatigue Severity Scale (FSS)....55
10.3 Short Form Health Survey SF-36....55
10.4 Expanded Disability Status Scale (EDSS) 55
10.5 Cranial Magnetic Resonance Imaging (MRI)....55

11. INVESTIGATIONAL PRODUCT....56
11.1 Dosage and Schedule of Test Drug 56
11.2 Maximum Tolerated Dose....56
11.3 Formulation, Packaging and Labeling 56
11.4 Preparation and Administration of Test Drug 56
11.5 Accountability of Drug Supplies 57
11.6 Recall of Investigational Product....57

12. SAFETY ISSUES....57
12.1 Warnings and Precautions 57
12.2 Adverse Events and Laboratory Abnormalities 57
12.2.1 Clinical Adverse Events....57

12.2.2 Severity Assessment ... 58
12.2.3 Laboratory Test Abnormalities ... 58
12.2.4 Serious Adverse Events (Immediately Reportable to Sponsor) ... 58
12.2.5 Treatment of Adverse Events ... 59
12.2.6 Causality Assessment ... 59
12.2.7 Follow-up of Adverse Events ... 60
12.2.8 Pregnancy ... 60

13. CRITERIA FOR PREMATURE WITHDRAWAL ... 60

14. STATISTICAL CONSIDERATIONS ... 61
14.1 Sample Size and Determination of MTD ... 61
14.2 Analysis Populations ... 61
14.2.1 Intent-to-Treat (ITT) Population ... 61
14.2.2 Safety Evaluable (SE) Population ... 61
14.2.3 Clinical Status (CS) Population ... 62
14.3 Data Handling ... 62
14.3.1 Measurement Times ... 62
14.3.2 Missing Data Conventions ... 62
14.4 Statistical Methods ... 62
14.4.1 General Overview and Plan of Analysis ... 62
14.4.2 Hypothesis Testing ... 63
14.4.3 Interim Analysis ... 63
14.5 Statistical Analysis ... 63
14.5.1 Disposition of Subjects ... 63
14.5.2 Baseline Characteristics ... 63
14.5.3 Analysis of Safety and Tolerability ... 64
14.5.4 Analysis of Clinical Status ... 65
14.5.5 Analysis of Pharmacodynamics (PD) ... 66
14.5.6 Exploratory Analyses ... 66

15. ETHICAL ASPECTS ... 66
15.1 Local Regulations/Declaration of Helsinki ... 66
15.2 Informed Consent ... 66
15.3 Independent Ethics Committee ... 67

16. CONDITIONS FOR MODIFYING THE PROTOCOL ... 67

17. CONDITIONS FOR TERMINATING THE STUDY ... 67

18. STUDY DOCUMENTATION, CRFS AND DATA HANDLING ... 68
18.1 Investigator's Files/Retention of Documents ... 68
18.2 Confidentiality of Trial Documents and Subject Records ... 68
18.3 Case Report Forms (CRFs) ... 68
18.4 Data Handling and Record Keeping ... 69

18.5 Assignment of Preferred Terms and Original Terminology 69
18.6 Study Centre Monitoring Visits 70
18.7 Quality Assurance and Regulatory Agency Audits 70

19. PUBLICATION OF DATA AND PROTECTION OF TRADE SECRETS 70

20. REFERENCES..........71

APPENDIX 1
MCDONALD CRITERIA FOR DIAGNOSIS OF MULTIPLE SCLEROSIS..........73

APPENDIX 2
NEW YORK HEART ASSOCIATION FUNCTIONAL
CLASSIFICATION (1994) 74

APPENDIX 3
TOXICITY GRADING SCALE FOR HEALTHY ADULT
AND ADOLESCENT VOLUNTEERS ENROLLED IN PREVENTATIVE
VACCINE CLINICAL TRIALS..........75

1. Background and Rationale

1.1 Scientific Background

It has been established that several innate immune responses are able to contribute towards the control of certain auto-immune based disorders, viral and bacterial infections, as well as certain types of cancer. These responses are orchestrated by multi-faceted mechanisms which include the induction of pro-inflammatory and anti-infective cytokines e.g TNF-α, IFN-α and IFN-γ, the production of bacteriostatic/bacteriocidal molecules such as α-defensins and reactive oxygen/nitric oxide intermediates, as well as the activation of a range of innate cell killing mechanisms such as the NK granzyme, perforin and FAS pathways. Specific activators of these pathways are therefore of potential therapeutic benefit[1].

The activation of innate immunity is in part controlled by pathogen recognition receptors (PRR) such as the Toll-like receptor and NOD receptor families[2, 3]. Each PRR recognizes distinct components associated with an infectious agent or diseased cell. Increasing knowledge of the ligands which activate individual PRRs, coupled with an increasing knowledge of their signaling cascades, have led to selective PRR ligands being developed as inducers of the preferred immune response depending on the nature of the disease[4].

Innate Therapeutics Ltd (the Sponsor) has designed and manufactured a stable, non-toxic, biodegradable microparticle immunostimulant, MIS416. MIS416 comprises covalently linked PRR ligands which preferentially signal through NOD-2 (nucleotide oligomerization domain-2) and Toll-like receptor 9 (TLR9), both of which have well described immunostimulatory properties for several immunologic cell types [5,6]. These include the induction of a wide range of pro-inflammatory and regulatory cytokines, chemokines and interferons, known to underpin innate and adaptive immune responses via a multiplicity of mechanisms. The incorporation of these PRR ligands into a 0.2 x 2.0 micron rod shaped microparticle results in substantial uptake by myeloid dendritic cells, plasmocytoid dendritic cells, granulocytes and monocytes/macrophages, all which have the functional capacity to respond to intracellular NOD-2 and/or TLR9-ligand-mediated signaling. Following internalization of MIS416, potent, broad spectrum NOD-2 and TLR9- dependant innate immune responses are rapidly mobilized.

The simultaneous production of immune regulatory cytokines such as TNF-α and IL-10, means that MIS416 is able to induce a more regulated immune response thereby avoiding hyper-immune stimulation that can be associated with immune-based mono-therapies. The microparticulate formulation restricts MIS416 uptake to key innate immune cell subsets thus avoiding clinically unacceptable side effects mediated by irrelevant cell types.

Preclinical studies using established animal models have identified that MIS416 has the potential to control a wide range of disorders when used as a standalone prophylactic or therapeutic agent. These include automimmune disorders, bacterial and viral infections as well as a range of cancers and associated metastases.

1.2 Study Drug

MIS416 is derived from gram-positive (LPS negative) *Propionibacterium acnes*, which have been biochemically modified such that certain potentially immunogenic as well as immunostimulatory cytosolic and cell wall components are selectively removed, generating an intact, biodegradable 0.2 x 2.0 μm rod shaped micro-particle comprising cross-linked muramyl dipeptide (MDP; *N*-acetylmurmyl-L-alanyl-isoglutamine) with covalently linked nuclease resistant bacterial DNA.

The microparticle is a potent activator of broad innate immune responses as a consequence of MDP and DNA mediated immune activation following cell uptake and biodegradation. MDP is the minimal structural unit of the peptidoglycan in the bacterial cell wall skeleton, and is known to stimulate innate immune cells via NOD-2, one of several cytosolic innate immune receptors that have been identified. *P. acnes* has the preferred peptide linkage for MDP immunostimulating activity, being an amino-linked L-alanine-D-isoglutamine dipeptide as opposed to a D-alanine-D-isoglutamine dipeptide, an inactive MDP stereoisomer associated with other microbial species. Incorporated bacterial DNA serves as a naturally occurring cytosolic TLR9 ligand which is an important immune receptor for the induction of additional, distinct innate immune responses.

MIS416 is reliably targeted to a range of innate immune cell subsets which together are responsible for the induction of both innate and adaptive immunity. This is achieved by the incorporation of these multiple immunostimulatory ligands as a stable, particulate formulation which exploits microparticle uptake mechanisms, principally phagocytosis, that are associated with immune cells.

1.3 Mode of action of MIS416

Second generation immunomodulators such as MIS416 are an emerging class of therapeutic agents for a number of therapeutic and prophylactic disease indications.

MIS416 is a specific immunomodulator comprising two classes of naturally occurring, non-engineered ligands which activate well-understood immunomodulatory pathways, resulting in the mobilization of a broad range of innate anti-infectious and anti-tumour defences in a well defined manner. Immunmodulators such as MIS416 represent an emerging class of drugs for the treatment of autoimmune disease, infectious disorders and cancer.

There are several categories of immunostimulants that can be classified by their origin and mode of action. Bacterial products (whole, killed organism, their lysates or

components of the cell wall) comprise one such class. This class is well known for its ability to increase the efficiency of the immune system response via specific and non-specific effects on the cellular and humoral compartments. For example, BCG (Bacille Calmette-Guerin) is the most commonly prescribed approved bacterial product immunostimulant, indicated for adjunct use in bladder cancer treatment. Extending the clinical application of unfractionated bacterial products such as BCG has been limited by their relatively undefined individual mechanisms of action and compositions.

More recently, the mechanisms of action of individual immunostimulatory bacterial components on the human immune system have been established. This has led to the development of second generation, more specific immunostimulants which are selected rationally, based on their known mechanism of action and immunomodulatory activity. Prototypic drugs in this category are CpG oligonucleotides, which target Toll-like receptor (TLR)-9; and Imiquimod, which targets TLR-7[7]. Murabutide has also been developed as an analogue for the bacterial peptidoglycan muramyl dipeptide[8,9]. Murabutide signals via nucleotide-binding oligomerization domain (NOD)-2, a well known intracellular pathogen recognition molecule forming part of the NODosome complex, which is central in the control of bacterial infection and inflammation. A variety of newly-recognized TLR ligands have been identified, such as urinary Tamm-Horsfall glycoprotein (a TLR4 ligand); siRNA (a TLR3 or 7 ligand); *Plasmodium falciparum* Hemozoin (a TLR9 ligand); and Profilin-like protein in *Toxoplasma gondii* (a TLR11 ligand). Common to all of these is their ability to activate the inflammatory wing of the innate immune system via nuclear factor–κB (NFkB) activation[10,16].

Whilst the mechanism of action of MIS416 is not novel, the development of a biodegradable microparticulate formulation of immunostimulatory ligands represents a critical advance in the clinical utility of specific, targeted immunostimulants.

Microparticles are considered a preferred means of delivering a wide range of immunomodulatory agents, from cytotoxic compounds to nucleic acid therapeutics such as RNAi. A key advantage of microparticle formulations is that uptake is restricted to cells capable of phagocytosis. Phagocytosis of microparticles (0.1 micron to 20 micron) is particularly associated with innate antigen presenting cells (APC) [11,12]. In support of this approach, biodegradable microparticles of alternate formulations are being developed for TLR9 immunostimulants such as CpG, to achieve immune cell targeting and improved therapeutic and safety outcome [13,14].

NOD-2 and TLR9 are both key microbial sensor proteins predominantly expressed by innate immune cell subsets. Monocytes, macrophages and myeloid dendritic cells, which are targeted by MIS416, are known to express NOD-2, whilst plasmocytoid dendritic cells express TLR9[13]. For full activation of innate immune defences such as anti-cancer activity, triggering of both of these receptors is critical. The tissues in which these cell types reside are predominantly the immune-associated tissues such as the liver and spleen as well as gut associated lymphoid tissues and mucosal lymphoid

tissues. The MIS416 microparticulate construct is designed to specifically target uptake by these cells, avoiding activation of irrelevant cell types.

Characterization of the microparticle targeting characteristics using non-purified blood mononuclear immune cells shows MIS416 is preferentially taken up by monocytes, plasmocytoid and myeloid dendritic APC (Figure 1-1).

Figure 1-1

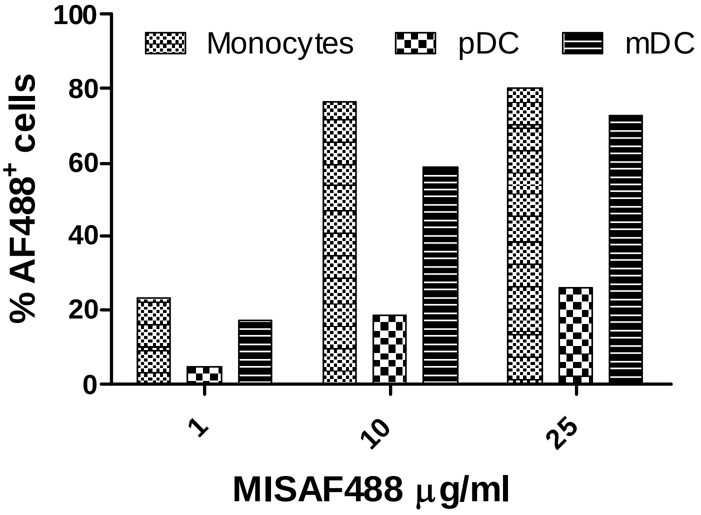

Internalization of fluorescently-labelled MIS416 by major human phagocytic innate immune cell subsets

Blood mononuclear cells were incubated with 50, 25, 10 or 1 µg/ml of AlexaFluor-488 labelled-MIS416 and incubated for 30 mins at 37°C. Monocytes, plasmocytoid (pDC) and myeloid dendritic cells (mDC) were identified using fluorescent monoclonal antibodies (CD45, BDCA-1, BDCA-2, lineage marker and CD14). Flow cytometry was used to determine the % of each subset that internalised AF488-MIS416.

In vivo administration of microparticles also leads to cell uptake preferentially by cells within the reticuloendothelial system. Flow cytometry based cell uptake studies using fluorescent labelled MIS416 in animal models have been used to demonstrate this cellular tropism is maintained *in vivo* following systemic delivery. Analysis of various immune compartments shows that the cells within the reticuloendothelial system preferentially sequester the microparticle (Figure 1-2).

Figure 1-2

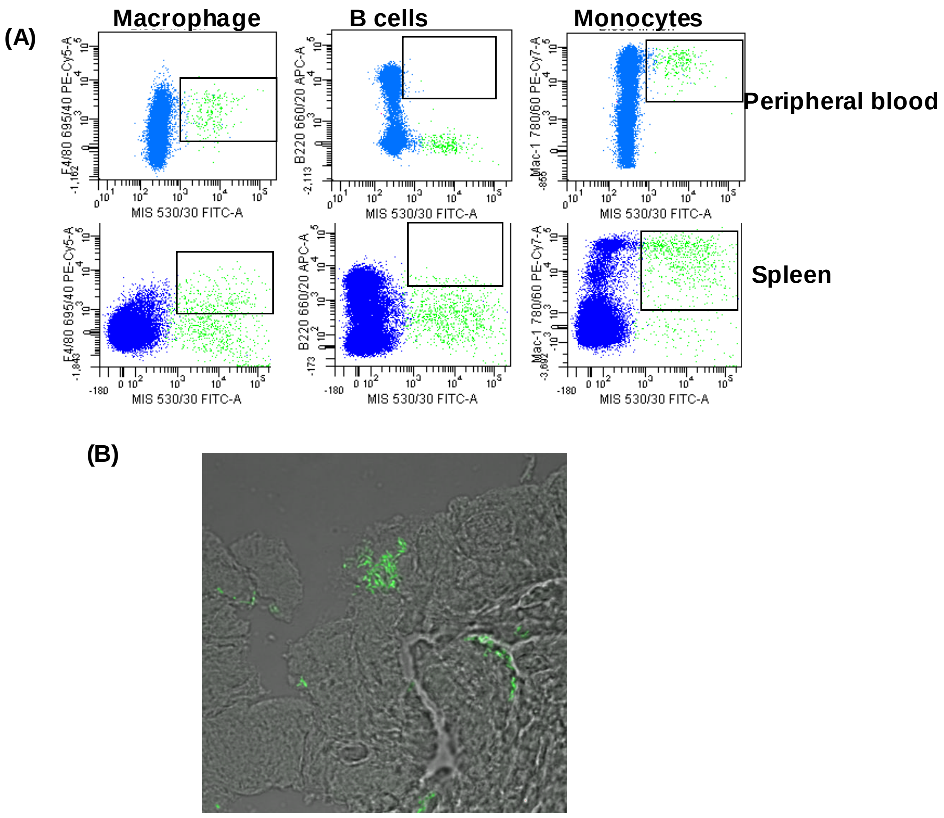

MIS416 is preferentially internalized by phagocytic mononuclear cells within the reticuloendothelial system following i.v delivery. Spleen, blood and liver samples were harvested at 6 hours following single bolus i.v delivery of 500 µg of fluorescent labeled MIS416 (AF488-MIS416). Cell suspensions were labeled with fluorescent antibodies and flow cytometry was used to identify the various cell subsets which had internalized the microparticle. Regions are drawn around cells which have internalized MIS416 (x-axis) and also express the cell subset marker of interest **(y-axis)** **(A)** Correlation of MIS416 fluorescence with cells expressing macrophage and 21onocytes fluorescent antibody lineage markers in blood and spleen by flow cytometry analysis of single cell suspensions..As a comparison, there is little or no association of MIS416 fluorescence with cells expressing B cell marker. **(B)** Liver sections (3 µm) were examined (63X) under fluorescence for AF488-MIS416 depot formation and under bright field for identification of Kupffer cells. Merging of the images shows AF488-MIS416 fluorescence correlates localizes in liver sinousoids. Uptake correlates with kupffer cell nuclear staining by H&E staining (data not shown).

The nature of the targets, together with the microparticle formulation of MIS416 mean that there is no precedence for there being any direct immunostimulatory activity of MIS416 upon B or T cells since these non-phagocytic cells are not capable of microparticle uptake. *In vivo* trafficking studies have verified this as previously

described (Figure 1-2A). That B and T cells are not directly or indirectly activated by MIS416 immunostimulation is supported by several independent bodies of evidence. *In vitro* human peripheral blood leucocyte assays demonstrate that MIS416 does not induce B cell proliferation or immunoglobulin secretion (Figure 1-3A, 1-3B).

Further, T cell proliferation, induced when donor mis-matched human lymphocytes are cultured with or without CD40 ligand, is repressed by MIS416 co-culture as opposed to enhanced (Figure 1-3C).

That MIS416 is not able to support T cell proliferation when administered systemically is further evidenced by the lack of induction of significantly increased amounts of T and B cell cytokines such as IL-2, IL-4 and IL-5 in patient blood serum during MIS416 immunotherapy (Figure 1-4). Thus, MIS416 microparticles neither home to, nor induce any functional effects on, B or T lymphocytes.

Figure 1-3

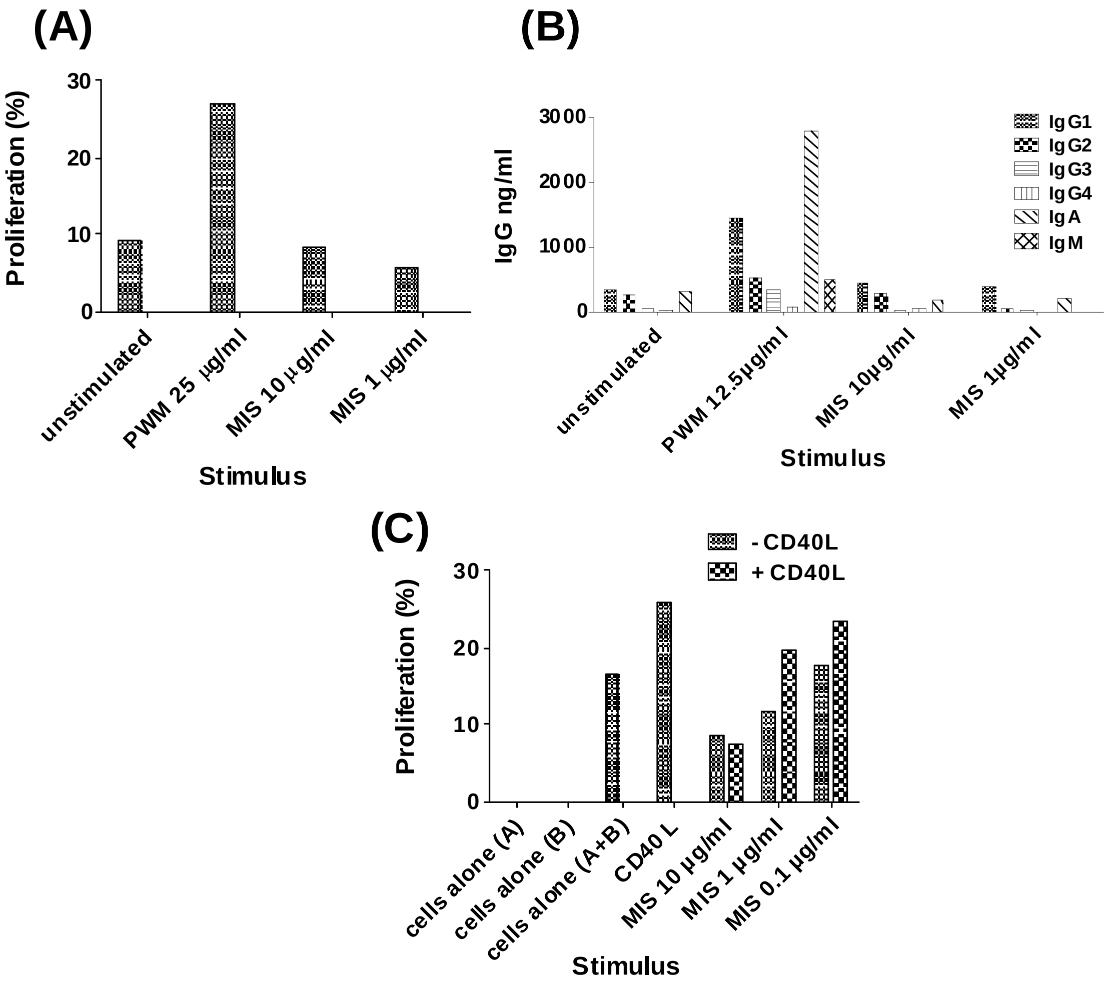

MIS416 does not indirectly stimulate B cell proliferation or immunoglobulin secretion and represses alloantigen induced T cell proliferation

(A) PBMC were fluorescently labeled with a fluorescent tracer of cell division (CFSE) and cultured in the presence of a known B cell activator (PWM) or MIS416 (10 and 1 µg/mL) for 7 days. PBMC were labeled with fluorescent antibodies reactive with B cells and viability dye. Live, proliferating B cells were quantified by flow cytometry analysis of CFSE fluorescence. When cells divide the intensity of CFSE fluorescence is reduced proportionally with each round of cell division. **(B)** Day 7 culture s/n were analyzed for immunoglobulin content using flow cytometry bead array methodology. **(C)** PBMC from two mis-matched donors were obtained. One cohort (responder) was labeled with CFSE, whilst the other cohort remained unlabelled (Stimulator). Equal cell numbers of stimulators and responders were co-cultured in the presence of MIS416 at 10, 1 and 0.1 µg/mL with or without soluble CD40L, a known T cell co-stimulation factor for 5 days. Cells were labeled with viability dye and fluorescent antibodies reactive with CD3 cells. Live responder T cells were identified based on CFSE/CD3 fluorescence and the % that had divided (diluted CFSE fluorescence) was determined.

Figure 1-4

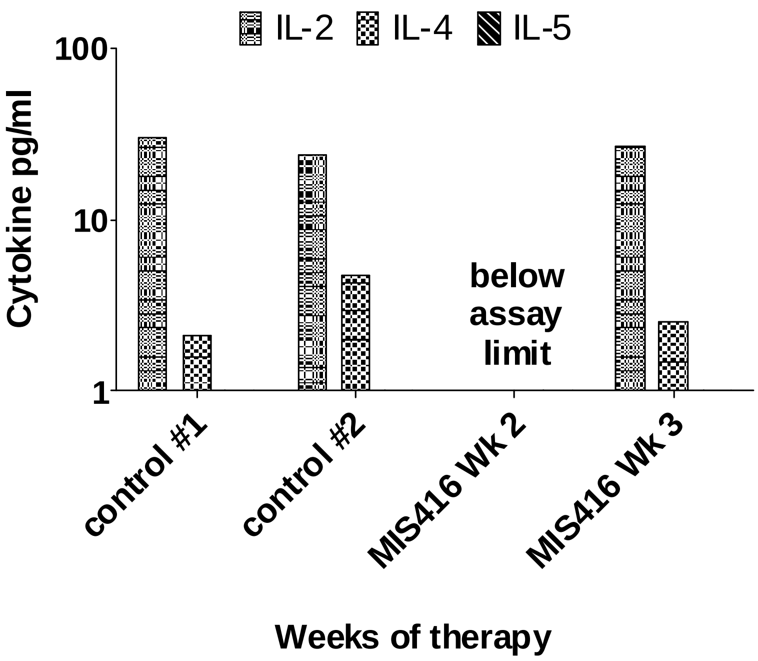

MIS416 therapy is not associated with the upregulation of cytokines associated with polyclonal T cell activation. Peripheral blood serum was harvested from two individual healthy donors and from a patient who had received 3 doses of MIS416 on a weekly basis. The blood was collected weekly just prior to administration of MIS416. Serum was assayed for IL-2, IL-4 and IL-5 using flow cytometry bead array methodology.

1.4 Pre-Clinical Experience

The innate immunomodulatory properties of MIS416 have been characterized extensively *in vitro* and *in vivo*. In vaccination models, MIS416 acts as a safe, potent cellular and humoral adjuvant without granuloma formulation or other adverse immune sequelae. When administered as a stand-alone prophylactic or therapeutic agent, MIS416 activates a broad range of innate host defense mechanisms which are able to prevent or ameliorate bacterial and viral infections in animal disease models. The ability of MIS416 to activate innate anti-neoplastic mechanisms has also been demonstrated in pre-clinical animal lung cancer studies.

A preliminary preclinical experimental autoimmune encephalomyelitis (EAE) animal model study identified that administration of MIS416 i.v at the time (d = 0) of immunization with MOG peptide in Freund's adjuvant s.c followed by pertussis toxin i.p on day 2 delays the onset and incidence of disease (Figure 1.5A) as well as reduces the disease score (Figure 1.5B). The maximum disease score is also significantly reduced in

this treatment group (Figure 1.5C). Treatment with MIS416 on day -3 relative to MOG immunization preferentially enhances the recovery phase from disease (Figure 1.5D).

Figure 1.5

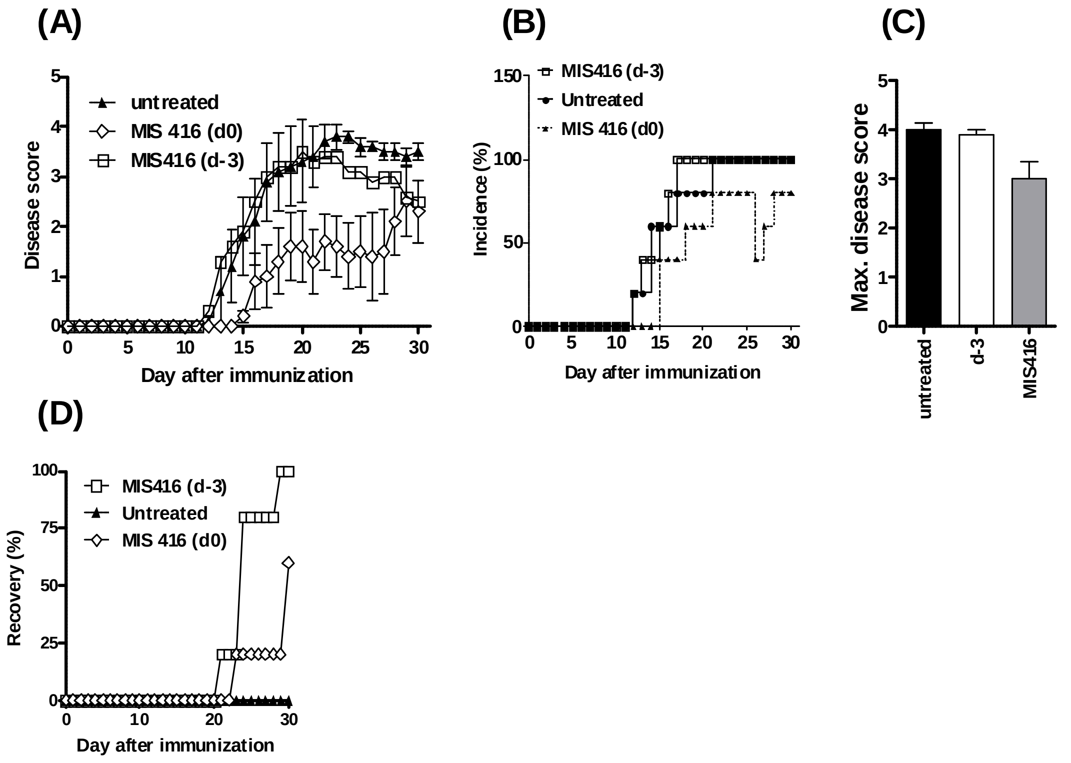

The ability of MIS416 therapy to modulate disease in mouse EAE model

When choosing animal models in which to evaluate test agents it is important to verify that the target receptor presumed to be present in the animal is present and that it recognizes the test agent ligand.

The ligands present on MIS416 are NOD-2 and TLR9. Both are evolutionarily conserved proteins present in all mammalian species[15]. These receptors recognize a unique pattern of molecules derived from pathogens or damaged cells, triggering robust but defined innate immune responses in all mammals.

The two species selected to evaluate acute and repeat dose toxicity arising from the administration of MIS416 were the out-bred mouse and rabbit. In common with all mammals, these two species have the NOD-2 and TLR9 receptor targets. In both species, these receptor targets also recognize the test agent ligands as evidenced by the extensive *in vitro* and *in vivo* mechanism of action studies in mice (Section 1.3) and the toxicity studies conducted in both these species. Specifically in both mice and rabbit

repeat dose toxicity studies, recognition of the test agent's ligands was clearly demonstrated by biological responses consistent with the items mechanism of action. These included a dose responsive induction of histiocytic proliferation in target organs, extra medullary hematopoiesis in target organs, and in rabbits, liver oval cell (stem cell) proliferation.

1.4.1 Acute Dose Studies

GLP single ascending dose acute toxicity animal studies have been conducted to establish a maximum tolerable dose (MTD) in Swiss albino mice and NZ white rabbits.

In summary, in mice, (n=5M & 5F per group) two deaths occurred in males in each of the mid and high dose groups (30 and 45 mg/kg respectively) whereas there were no mortalities in the low and intermediate dose groups (10 and 15 mg/kg respectively). There was no treatment related gross pathology in males or females in any groups. Histopathology evaluation showed treatment related, dose dependent histiocytic proliferation in liver, increased extra medullar hematopoiesis in spleen, granulocytic infiltration in mandibular lymph nodes & leukocytic infiltration at site of injection in both males & females in all treated dose groups.

In view of the results observed, the MTD for MIS416 when administered as a single dose to Swiss albino mice by i.v. route was determined to be 15 mg/kg bodyweight under the test conditions and doses employed.

In rabbits (n=5M & 5F per group) dosed at 50, 200, 800, or 3200 µg/kg, there were no toxic signs or mortalities. There was no treatment related gross pathology in males or females in any groups. There was no treatment related microscopic changes in males or females in the high dose group (3200 µg/kg) compared to the control group. The maximum dose of 3200 µg/kg was selected based on earlier gross pathology findings in a non-GLP acute study in NZ white rabbits (n=2M & 2F per group) wherein bolus doses of 6, 18, 55, 166, and 500 µg/animal were tested. These doses approximated to 2.5, 7.5, 23, 70, and 200 µg/kg. In this study gross pathology was observed in spleen and liver of the high dose (200 µg/kg) group. A high dose of 3200 µg/kg was established for the GLP single ascending dose acute toxicity animal study on the basis that a 16-fold increase over the non-GLP study high does should have been sufficient to establish a MTD.

It did not, however the subsequent GLP four week repeated dose toxicity study did find significant pathology and one mortality (n=4M & 4F per group) in the high dose (5000 µg/kg) group. This repeat dose study found a NOAEL of greater than 50 µg/kg and less than 500 µg/kg bodyweight under the test conditions and doses employed.

1.4.2 Repeat Dose Studies

GLP four week twice weekly and twenty six week once weekly repeated dose toxicity animal studies have been conducted to evaluate potential toxicity, look for corollaries with the protective effects of the test item previously identified *in vitro* and *in vivo*, and

provide an estimate of No Observed Adverse Effect Level (NOAEL). The four week studies were conducted in Swiss albino mice and NZ white rabbits and the twenty six week study was conducted in NZ white rabbits.

1.4.3 4 Week Repeat Dose Studies

In mice (n=10M & 10F per group), there were were no treatment related adverse effects in the low dose group (1 mg/kg). In the mid dose group (3 mg/kg) treatment related gross pathology, an enlarged spleen, was found in one female and considered an adverse effect. Histopathology findings comprised a grade 1 lymphocytic infiltration in the urinary bladder in one female, a grade 3 histiocytic proliferation in liver in one female and grade 3 extramedullary hematopoiesis in liver in one female. These were considered adverse effects.

In the high dose group (10 mg/kg) treatment related gross pathology, enlarged spleens, were found in males and females. Histopathology findings comprised grade 1 and grade 2 lymphocytic infiltration in the urinary bladders of males and females, greater than grade 2 histiocytic proliferation in livers in males and female and greater than grade 2 extra medullary hematopoiesis in livers and spleens of males and females. These were considered adverse effects.

In view of the results NOAEL for MIS416 when administered twice weekly for 4 weeks to Swiss albino mice by i.v. route is greater than 1 mg/kg and less than 3 mg/kg bodyweight under the test conditions and doses employed.

In rabbits (n=4M & 4F per group), one death occurred in males in the high dose group (5000 μg/kg) whereas there were no mortalities in the low and mid dose groups (50 and 500 μg/kg respectively).

In the low dose group, desirable effects, consistent with the test items mode of action, were grade 1 histiocytic proliferation (activation of macrophages) in liver, grade 1 oval cell proliferation (activation of liver stem cell), and grade 1 bone marrow hypercellularity. Findings of grade 1 or grade 2 lymphoid hyperplasia in spleen in all males and females and grade 1 increased hemosiderous in spleen in a single female were considered treatment related but not adverse effects.

In mid and high dose groups there were findings of grade 1 and grade 2 desirable effects, consistent with the test items mode of action, comprising histiocytic proliferation in liver, lung, and spleen, oval cell proliferation, and bone marrow hypercellularity. Adverse findings, in particular in the high dose group, included treatment related adverse hematology and chemistry, organs weights, gross pathology and histopathology including erythrophagoctosis in liver and spleen, increased hemosiderosis (greater than grade 1) in spleen, lymphoid depletion in spleen, endothelial hyperplasia in lungs and eyes, and glomerular hypercellularity in kidneys.

In view of the results NOAEL for MIS416 when administered twice weekly for 4 weeks to New Zealand white rabbits by i.v. route is greater than 50 µg/kg and less than 500 µg/kg bodyweight under the test conditions and doses employed.

1.4.4 26 Week Repeat Dose Study

In this GLP repeat dose toxicity study, respective groups of 6 animals per sex per group were administered MIS416 intravenously once weekly for 26 weeks with doses of 0 ug (control), 20 ug, 200 ug or 1000 ug /kg. Following the treatment period, 2 animals per group were held for a 4 week recovery period.

At the lowest dose of 20 µg/kg there were no deaths. Principal effects included minimal leukocyte infiltration into the choroid of the eye, minimal portal inflammation in the liver, with no effects on liver enzymes, minimal mixed leukocyte infiltration in the lung in 2/8 animals, and lymphoid hyperplasia of the spleen in one of eight animals. Absolute and relative spleen weights were increased as well.

At the middle dose (200 µg/kg) there was no mortality whereas at the high (1000 µg/kg) dose three animals died or were sacrificed when observed to be in a moribund condition during the fourth and fifth month. Cause of death was attributed to cardiac myofibre degeneration and necrosis. This finding, or any cardiac findings, was not found in any other surviving animal at any dose. Additional effects seen at these doses included small but statistically significant reductions in red cell parameters, slight decrease in platelet counts, increased globulin values with a corresponding decrease in Albumin/Globulin (A/G) ratio, and decreased calcium (females only). Although occasional random increases in liver enzyme levels were seen in some animals, there was no clear nor consistent effect. Histopathological evaluation of tissues showed effects in the spleen (hyperplasia of the germinal center and hyperplasia of the red pulp), liver (portal inflammation, with necrosis and fibrosis at the high dose only), lung (mixed leukocyte infiltration), eye (mixed leukocyte infiltration of the choroid), and kidney (tubular degeneration and regeneration, glomerulopathy and heterophil infiltration in high dose animals only).

In conclusion, the intravenous administration of MIS416 to rabbits on a weekly basis resulted in toxicological effects at the high dose that included mortality (3/8 animals), generally mild clinical pathology findings and histopathological effects in the eye, heart, kidney, liver, lung and spleen. These findings had either resolved or showed advancing resolution at the end of the four-week recovery period. Some of these findings, except mortality, were noted at the middle dose. Effects noted at the lowest dose of 20 ug/kg/dose were substantially reduced in incidence, and were limited to minimal leukocyte infiltration in the eye, minimal portal inflammation in the liver (no effects on liver enzymes) and lymphoid hyperplasia in the spleen in 1/8 animals. Thus, the dose of 20 ug/kg/dose is considered the NOAEL for the administration of MIS416 weekly for 26 weeks.

1.4.5 Animal Toxicology Studies Conclusions

Results from the above summarized studies to evaluate potential toxicity, look for corollaries with the protective effects of the test item previously identified in vitro and in vivo, and provide an estimate of MTD and NOAEL, support the selection and relevance of the animal species used, namely mice and rabbits. Certain histopathological and histology findings in both species were fully consistent with the test agent's mode of action indicating that the cellular targets and intracellular target ligands, were present in both species. Furthermore, the use of rabbits to evaluate agents with the potential to induce significant and unwanted inflammatory responses is well established.

In-human experience with MIS416 to date (see below) has established a desirable dose range from 1 µg/kg to 10 µg/kg based on inducing an initial pro-inflammatory response as evidenced by grade 1 to grade 2 flu like symptoms, in particular fever and chills. Comparing the in-human high dose (10 µg/kg) with the mouse MTD of 15000 µg/kg and the rabbit MTD of 3200 µg/kg suggests significant margins of 1500 times and 320 times respectively.

A comparison of the same in-human dose (10 µg/kg) with the mouse NOAEL of 1000 µg/kg and the rabbit NOAEL of 50 µg/kg further suggests margins of 100 times and 5 times respectively, when MIS416 is administered weekly for 4 weeks. For the administration of MIS416 weekly for 26 weeks, the comparison of the same in-human high dose (10 ug/kg) with the rabbit NOAEL of 20 ug/kg provides a safety margin of 2 times.

However, it should be noted that, in the 26-week rabbit study, the effects seen at the middle dose of 200 µg/kg were primarily a result of extended pharmacological effects, were not explicitly adverse, and that the NOAEL could be considered to be 200 µg/kg, or provide a margin of 20 time the human dose.

1.5 Clinical Experience

MIS416 has been used in New Zealand as an unapproved experimental medicine ("compassionate use"), under sections 25 and 29 of the Medicine's Act, 1981, in a total of twelve patients with a range of medical conditions, including polyarteritis nodosa, advanced colorectal and squamous cell carcinomas, progressive multiple sclerosis and Alzheimer's Disease. Individual weekly doses have ranged from 150 µg to 5000 µg and treatment has continued for up to 10 years in one case (Table 1).

Table 1 Summary of Patients Dosed Under Compassionate Use (correct as of 08 June 2011)

Patient	Condition	Number of Doses	Max. Dose (bolus)	Cum. Dose	Period
01	Polyarteritis Nodosa	> 150	5000 µg	> 45,000 µg	10 yrs
03	Secondary Progressive Multiple Sclerosis (SPMS)	66 (ongoing)	1500 µg	40.975 µg	131 wks
04	Metastatic colorectal cancer	29	600 µg	13,600 µg	32 wks
05	Primary Progressive MS (PPMS)	47 (ongoing)	625 µg	22.200 µg	118 wks
06	PPMS	19	500 µg	4,750 µg	38 wks
07	Alzheimer's disease	72 (ongoing)	700 µg	37.100 µg	117 wks
08	Non Hodgkins Lymphoma	18	500 µg	7,900 µg	20 wks
09	PPMS	40	625 µg	18.375 µg	70 wks
10	SPMS	19	750 µg	10,165 µg	24 wks
11	PPMS	16	500 µg	6,500 µg	35 wks
12	PPMS	54 (ongoing)	500 µg	23.100 µg	92 wks
13	SPMS	27(ongoing)	100 µg	1.730 µg	35 wks

Patients who have been treated with MIS416 under section 25 have been monitored for safety by their physician through taking baseline and regular blood samples for both standard clinical laboratory evaluations and serum cytokine evaluation by the Sponsor. The only significant drug related adverse effect to date has been transient grade 1 to grade 2 flu-like symptoms together with transient muscle weakness. These effects are expected given the mode of action of the drug and are also reported as the common adverse effect arising from treatment with approved interferons and cytokines. Two patients who escalated dosing to 750 to 800 µg for 3 or 4 weekly treatments reported feeling generally unwell. Blood samples from one of these patients indicated significantly elevated levels of TNF-α. Upon dose reduction to 500 µg, these signs and symptoms resolved or reduced to normal levels.

The nature of the target for MIS416 and the mechanism of action are such that following *in vivo* administration and accumulation of the drug by the reticulo-endothelial system, a transient inflammatory response can be expected. Monitoring of patient serum for increased levels of pro-inflammatory cytokines demonstrates the transient nature of MIS416 immunostimulation (Figure 1-6). These findings are very important since they demonstrate that administration of a single bolus of MIS416 does not induce an uncontrolled “cytokine storm”, with maximum levels of serum cytokines peaking within 24 hour post delivery, returning to baseline levels by 48 hours.

Analysis of MIS416 - treated patient blood for a sustained inflammatory response, or the occurrence of a “second wave” of inflammatory activity over a longer time period (14 days) demonstrates there is no prolonged inflammatory activity following MIS416

dosing. This further demonstrates that MIS416 innate immunostimulation is well controlled *in vivo* (Figure 1-7).

Figure 1-6

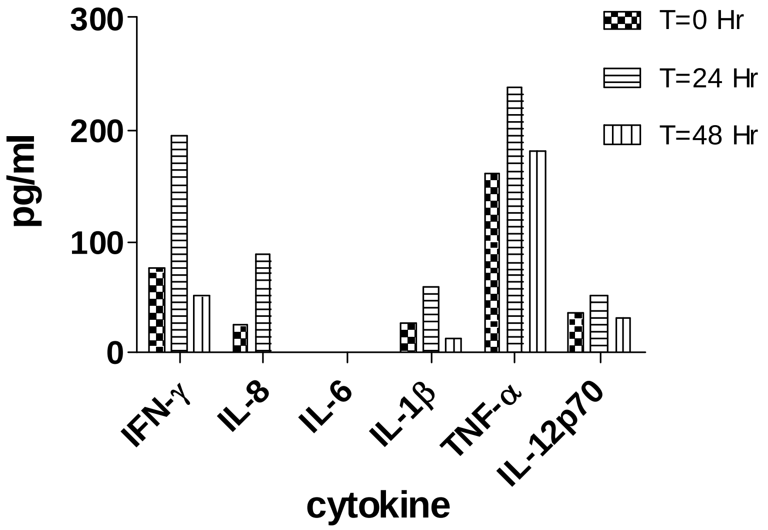

MIS416 systemic administration induces a transient serum inflammatory cytokine response which peaks within 24 hours following administration. Blood serum was harvested and directly measured for multiple cytokine content using flow cytometry cytokine bead array methodology (Bender MedSystem)

Figure 1-7

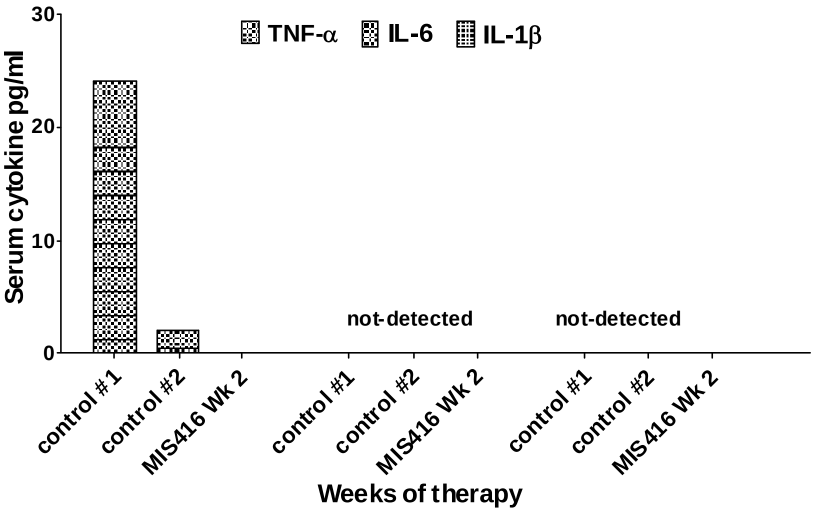

MIS416 therapy does not lead to the sustained induction or a "second wave "of inflammatory mediators. Peripheral blood serum was harvested from two individual healthy donors and from a patient who had received 1 mg of MIS416 7 days prior to blood sampling. Blood serum was assayed for TNF-α, IL-6 and IL-1β levels using flow cytometry bead array methodology.

2. Rationale for Study Design

2.1 Developmental Context

Immunomodulators (or immune response modifiers) are currently under investigation and/or approved to treat a range of indications including inflammatory and autoimmune disorders - examples include Interferon beta for treatment of relapsing-remitting Multiple Sclerosis (MS).

It is the intention to develop MIS416 further within the field of progressive MS, due to the promising results seen with compassionate use to date and the paucity of new agents appearing in this therapeutic area.

The study aims to determine the safety and tolerability of ascending multiple doses of MIS416, administered intravenously to patients with chronic progressive multiple sclerosis (MS). From this information, dose-limiting toxicity (DLT), maximum tolerated dose (MTD) and recommended Phase 2 dose (RP2D) will be defined.

Clinical experience with MIS416 to date has established a desirable dose range from between approximately 2 µg/kg to 14 µg/kg based on inducing an initial pro-inflammatory response as evidenced by grade 1 to grade 2 flu like symptoms, in particular fever and chills.

In the event that the study demonstrates that MIS416 is well tolerated and safe, the next step in the clinical development of the agent would be to conduct a randomized, blinded, controlled Phase 2B trial of the efficacy and safety of MIS416 in the treatment of patients with Secondary Progressive Multiple Sclerosis.

2.2 Rationale Dose-Escalation Phase

This dose-range finding study in a progressive MS patient group will enable the determination of a recommended Phase 2 dose for the controlled trial cited above and build on the early data available with regard to pharmacodynamics in this area. Due to the nature of the disease and the once-weekly administration of MIS416, a staged dose-escalation design has been incorporated into this first-in-man study with the goal of selecting a pharmacologically active but well-tolerated dose level (i.e., the MTD for 4 weeks of treatment) for further testing.

2.3 Rationale Dose-Confirmation Phase

Once such dose selection occurs, the intent of the dose-confirmation phase of this trial is to create a safety database sufficient to support longer-term exposure in the planned efficacy trial. This will be accomplished by (a) expanding the patient population exposed to this dose level and (b) prolonging exposure to 3 months. This will allow confirmation of the longer-term safety and tolerability of MIS416 before exposing a much larger population to a lengthier dosing regimen in the planned efficacy trial. Given the variable but generally progressive nature of multiple sclerosis, patients treated for 3 months will be observed closely not only for standard safety outcomes but also for changes in underlying disease status – both to rule out any adverse effect of MIS416 on disease progression and to document any anecdotal changes that may inform the selection of patients and/or endpoints for the initial efficacy trial.

2.4 Selection of Starting Dose (DE Phase)

A starting dose of 125 µg (or approximately 2.5 µg/kg based on a 'worst case' estimate of 50 kg patient weight) has been selected for the study. This starting dose is justified with reference to:

a). The simple margin between NOAEL's observed in the animal toxicology studies expressed as dose per weight. In the rabbit, the most sensitive species, this simple margin was 20-fold as recorded in the following table:

Table: Simple Margin NOAEL's to Human Starting Dose (2.5 µg/kg)

**Safety Study**	**Reported NOAEL**	**Margin** (based on bottom of the NOAEL range)
Mice: (Twice weekly, 4 weeks)	> 1000 µg/kg but < 3000 µg/kg	400 x
Rabbits (Twice weekly, 4 weeks)	> 50 µg/kg but < 500 µg/kg	20 x

N.B.: In these safety studies, both mice and rabbits were dosed twice weekly whereas dosing in the proposed study in patients is once weekly.

b). The safety factor between the proposed starting dose, being a weekly exposure, and the conversion of equivalent (weekly) animal exposure to human equivalent doses (HED) based on body surface area and a 60 kg human. In rabbit, this safety factor, based on weekly exposure, is 15-fold as recorded in the following table. This exceeds the 'default safety factor' of 10 contained in FDA Guidance "Estimating the Maximum Safe Starting Dose in Initial Clinical Trials for Therapeutics in Adult Healthy Volunteers" issued July 2005. N.B. The conversion factors contained in the Guidance reference a 60 kg human, thus the proposed human starting dose has been expressed as 2.1 µg/kg to maintain equivalence.

Table: Safety Factor of HED to Human Starting Dose (2.1 µg/kg or 0.0021 mg/kg) based on weekly exposure

**Safety Study**	**NOAEL** (based on twice weekly dosing)	**Conversion Factor** (animal dose in mg/kg to HED in mg/kg)	**HED** (mg/kg)	**Human Equivalent Weekly Exposure** (mg/kg)	**Safety Factor** (based on weekly exposure of 0.0021 mg/wk)
Mice:	1 mg/kg	0.08	0.08	0.16	76 x
Rabbits	0.05 mg/kg	0.32	0.016	0.032	15 x

c). The limited 'compassionate use' experience to date in humans with progressive MS. The following table records for each of these patients the starting dose, maximum dose, and total number of doses received to date.

Table: Human Compassionate Use Doses (bolus dose)

Patient ID#	Starting Dose	Maximum Dose	Number of Doses
03	150 µg*	1500 µg	46
05	100 µg	625 µg	29
06	50 µg	500 µg	19
09	100 µg	500 µg	30
10	100 µg	750 µg	19
11	100 µg	500 µg	16
12	100 µg	500 µg	28

# Patient ID's in accordance with Table 5-1 of the Clinical Investigator's Brochure Edition 3.

* Patient 03 inadvertently received greater than the intended initial dose due to a drug dilution error, however received the intended 150 µg dose as their second dose.

d). The proposed starting dose of 125 µg (or 2.5 µg/kg) is 20 times less than the NOAEL observed in rabbits, a species known to be highly sensitive to immunological test agents. When the animal NOAEL is adjusted for body surface area and the difference between twice weekly exposure in animals normalized to the proposed weekly exposure in the clinical trial, the safety factor is greater than 10-fold suggested in the FDA guidance document.

e). The proposed starting dose is also four times less than the lowest maximum dose (500 µg) administered to any of the seven MS patients treated to date, none of whom have experienced a "DLT-equivalent" at such a dose (500 µg).

3. Objectives of the Study

The primary objectives of this study are:

1. To determine the safety and tolerability, dose-limiting toxicities (DLTs), maximum tolerated dose (MTD), and recommended Phase 2 dose (RP2D) of intravenously (IV) administered MIS416 weekly in patients with chronic progressive multiple sclerosis (CPMS); and
2. To assess the pharmacodynamic (PD) effects of MIS416, including effects on serum cytokine levels and peripheral blood mononuclear cell (PBMC)- associated cytokine/chemokine expression patterns.

The secondary objectives of this study are:

1. To document any changes in MS clinical status occurring during the 12-week MIS416 dosing period in the dose-confirmation phase, as determined by the Multiple Sclerosis Functional Composite (MSFC), Fatigue Severity Scale (FSS), Short Form Health Survey (SF-36), and Expanded Disability Status Scale (EDSS); the frequency of clinical relapses; and signs of clinical activity on serial cranial MRI scans; and
2. To evaluate, in exploratory fashion, any correlations between clinical, radiological and PD outcomes.

4. Study Design

4.1 Overview of Study Design

This is a single center, open-label, non-randomized, dose-escalation study, to be conducted in two phases: a dose-escalation (DE) phase, to evaluate the safety, tolerability, MTD, and PD of MIS416 administered IV once weekly for 4 doses; and a dose-confirmation (DC) phase, which will be a cohort expansion at or below the MTD (i.e., the RP2D) of MIS416, dosed once weekly for up to 12 doses.

Subjects will be treated with a weekly IV dose of MIS416 in 28-day cycles: 1 cycle in the DE phase, and up to 3 cycles in the DC phase. The initial dose of MIS416 will be 125 µg/week. Subjects will be evaluated and dosed weekly each cycle in each phase. Subjects will return for a follow-up visit 15 days after completion of the last dose of study drug.

4.2 Number of Subjects Planned

The total number of subjects to be enrolled in the study is dependent upon the observed safety profile, which will determine the number of subjects per dose level, as well as the number of dose escalations required to achieve the MTD. It is anticipated that approximately 27-33 subjects will be enrolled in this study, with approximately 12-18 subjects in the dose-escalation phase and up to a further 15 subjects in the dose-confirmation phase. The study will be conducted at a single site.

4.3 Dose Escalation Phase (1 cycle only)

In the DE phase, the dose of MIS416 will be escalated in successive cohorts of 3 subjects per dose level. The first subject of each cohort will be dosed at least 48 hours prior to the rest of the cohort. If none of the 3 subjects at a dose level experience a DLT (see Section 4.4), then 3 new subjects may be entered at the next higher dose level. If 1 of 3 subjects experiences a DLT, 3 more subjects will be started at the same dose level (total of 6 subjects in cohort). If no additional subjects experience DLT at that dose, the next

higher dose level cohort will be initiated. If 2 or more subjects experience DLT, no further subjects will be started at that dose, MTD will be declared, and dose-escalation will cease (subject to confirmation by the Safety Review Team).

In the event that a single subject in a cohort experiences a DLT, the dose escalation increments will be reduced by at least 50% in the subsequent cohort. Any subject experiencing a DLT during the DE phase will be withdrawn from the trial.

The MTD is defined as the highest dose level at which < 2 of 6 subjects develop DLT. New dose levels may begin accrual only if all subjects at the current dose level have been observed for a minimum of 7 days from the 4th dose of the Cycle 1. The RP2D will be the MTD; if no MTD is reached, RP2D will be determined based upon evaluation of all clinical and PD data.

4.4 Dose Limiting Toxicity (DE phase only)

A DLT is a clinically significant adverse event (AE) or laboratory abnormality assessed by the Co-Principal Investigators as at least possibly related to the study drug and unrelated to disease progression, concurrent illness, or concomitant medications, occurring within Cycle 1. Toxicity will be graded and recorded according to the Toxicity Grading Scale for Healthy Adult and Adolescent Volunteers Enrolled in Preventative Vaccine Clinical Trials (presented in the companion Operations Manual), modified to include a systemic line item for rigors/chills and an amendment to the classification of pyrexia (Appendix 3). A DLT is defined as:

- Grade 3 or 4 adverse event, except for fatigue and fever and/or rigors/chills
- Grade 4, or an increase above baseline of 2 or more grades, fatigue
- Grade 4 fever and/or rigors/chills.

4.5 Dose Confirmation Phase (up to total of 3 cycles)

<u>The DC phase will not begin until the Sponsor has submitted to the Ethics Committee and regulatory authority the requisite sub-chronic nonclinical safety data, and received approval to proceed to 3 cycles of dosing.</u>

In the DC phase, up to 15 subjects will be recruited at the RP2D. The Safety Team will have the responsibility of reviewing safety data from the DC phase of the trial and making recommendations regarding dose modifications. If a DC phase subject withdraws from dosing within the first 2 cycles for reasons unrelated to drug toxicity, the subject may be replaced at the discretion of the Safety Review Team.

4.6 Intra-Subject Dose Reduction (DC phase only)

An individual subject's dose may be reduced to the next lower cohort dose level (ie, as determined from the DE phase), at the Co-Principal Investigator's discretion and with

the approval of the Sponsor, following a grade 4 or other clinically significant toxicity. The dose may not be reduced below 125 µg/week.

4.7 Antipyretic Pre-Medication (DC phase only)

Subjects who experience an adverse event of pyrexia or rigors/chills (grade 3 or less) may receive the same dose level in subsequent dosing visits with antipyretic pre-medication, at the discretion of the Co-Principal investigators.

4.8 Safety Review Team

A Safety Review Team (SRT), comprising the Co-Principal Investigators, the Sponsor's Medical Monitor and an independent clinical expert will be empanelled. At the conclusion of each cohort in the DE phase, the SRT will meet by teleconference to determine from all available clinical safety and PD data:

- Whether the next cohort should be initiated
- If so, the increment of dose escalation
- If not, that the MTD has been achieved.

The SRT will further determine the progression to, dose, and size of the RP2D cohort; oversee subject safety during the DC phase; and recommend DC dose modifications as needed.

5. Study Population

5.1 Target population

The study aims to recruit between 27-33 male and female subjects over the age of 18 years, with a diagnosis of primary progressive or secondary progressive MS. All subjects who have received any quantity of study drug, whether withdrawn prematurely or not, will be included in the final safety analysis.

Subjects who receive study drug and are subsequently withdrawn are not permitted to be enrolled for a second time.

5.2 Inclusion Criteria

1. At least 18 years of age.
2. Diagnosis of MS, by the McDonald criteria[17]. (See Appendix 1)
3. Chronic progressive MS (CPMS), defined as either primary progressive MS (PPMS) or secondary progressive MS (SPMS), per the criteria of the National Multiple Sclerosis Society (USA) Advisory Committee on Clinical Trials of New

Agents in Multiple Sclerosis[18]. [NOTE: In the dose-confirmation phase, only subjects with SPMS may be enrolled].

4. MS is clinically active with worsening clinical status within the past 2 years.
5. Expanded Disability Status Scale (EDSS) of 2.5 to 7.0 at Screening.
6. The following laboratory values must be documented within 3 days prior to initiation of study drug:
    - Absolute neutrophil count (ANC) $\geq 1 \times 10^9$/L
    - Platelet count $\geq 100 \times 10^9$/L
    - Serum creatinine $\leq$ 1.5 mg/dL
    - AST (SGOT) and ALT (SGPT) $\leq 2 \times$ upper limit of normal.
7. Provide written informed consent to participate.
8. Willing to comply with scheduled visits, treatment plans, laboratory assessments, and other study-related procedures.

5.2 Exclusion Criteria

1. Relapsing-remitting MS or progressive-relapsing MS
2. Any immunomodulatory drug therapy or immunosuppressive therapy within the previous six months, or vaccine or systemic corticosteroids within the previous 60 days, prior to initiation of study drug.
3. Exposure to other experimental treatments currently under investigation in MS clinical trials, including alemtuzamab, rituximab, fingolimod, and cladribine.
4. A diagnosis or history of collagen vascular disease (including Sjögren's syndrome and systemic lupus erythematosus), anticardiolipin antibody syndrome, cerebral autosomal dominant arteriopathy with subcortical infarcts and leukoencephalopathy (CADASIL), sarcoidosis, vasculitis, Bechet's syndrome and/or Lyme disease.
5. History of alcohol or drug abuse (with the exception of cannabinoids) within 2 years prior to initiation of study drug.
6. Inability to refrain from smoking for the duration of the inpatient stay.
7. Major surgery or radiation therapy within 28 days prior to initiation of study drug.
8. Active infection requiring antibiotics within 2 weeks prior to initiation of study drug.
9. Active malignancy within 2 years of entry, with the exception of basal cell carcinoma and squamous cell carcinoma of the skin
10. Uncontrolled congestive heart failure (New York Heart Association Classification 3 or 4 [Appendix 2]), angina, myocardial infarction, cerebrovascular accident, coronary/peripheral artery bypass graft surgery, or transient ischemic attack within 3 months prior to initiation of study drug.

11. Symptomatic cardiac dysrhythmias requiring treatment, or persistent prolongation of the QTcF (Frederica) interval to > 450 msec for males or > 470 msec for females.
12. In the Dose-Confirmation phase only, screening echocardiogram which shows abnormalities judged by the investigator to be clinically significant, including but not limited to a left ventricular ejection fraction below the testing facility's lower limit of normal.
13. Pregnant or lactating female.
14. Women of childbearing potential, unless surgically sterile for at least 3 months (i.e., tubal ligation), postmenopausal for at least 12 months (and follicle-stimulating hormone [FSH] > 20 U/mL), or unless they agree to use effective, dual contraceptive methods (i.e., oral, injectable, or barrier method with male partner using a condom) while on study drug.
15. Men of childbearing potential who partner with a woman of childbearing potential, unless they agree to use effective, dual contraceptive methods (i.e., a condom, with female partner using oral, injectable, or barrier method) while on study drug and for three months following discontinuation of study drug.
16. Known human immunodeficiency virus or acquired immunodeficiency syndrome-related illness.
17. Serological evidence of Hepatitis B, C or HIV infection.
18. Any severe, acute or chronic medical or psychiatric condition or laboratory abnormality that may increase the risk associated with study participation or study drug administration, may interfere with the informed consent process and/or with compliance with the requirements of the study, or may interfere with the interpretation of study results and, in the investigator's opinion, would make the patient inappropriate for entry into this study.
19. Previous exposure to MIS416.

5.4 Concomitant Medication

Subjects are not permitted any immunomodulatory drug therapy or immunosuppressive therapy within the previous six months, or vaccine or systemic corticosteroids within the previous 60 days, prior to initiation of study drug, or throughout the study.

Medications used to treat pre-existing conditions are permitted, provided the dose has been stable for one month prior to initiation of study drug and they must be recorded in the CRF. Medications required to treat adverse events are permitted throughout the study and must be recorded in the CRF.

5.5 Restrictions

Strenuous activity which may lead to dehydration, will not be permitted for 24 hours prior to dosing on all study days throughout the study.

Smoking and alcohol consumption will not be permitted during the inpatient stay in the unit.

Caffeine- containing food and drink will be restricted while in the unit.

6. Schedule of Assessments and Procedures

Refer to Schedule of Events:

- Table 2 DE Phase
- Table 3 DC Phase (Cycle 1)
- Table 4 DC Phase (Cycles 2 and 3)

6.1 Screening (Days -28 to -2)

Subjects who are willing to participate in the study and who have signed a Consent Form will be screened for eligibility within 28 days of dosing.

Screening tests will include a record of medical history, a physical examination (excluding breast, genital or rectal examination, unless clinically indicated), vital signs, weight, height, ECG, safety laboratory tests (haematology, biochemistry, pregnancy test and/or FSH where applicable, hepatitis B, C and HIV serology, urinalysis) and drug of abuse screen. An Expanded Disability Status Scale will be completed (see Section 9.4). A cranial MRI scan will be performed during the screening period.

Subjects who meet all of the inclusion criteria and none of the exclusion criteria will be accepted onto the study.

Documentation of the subject's fulfillment of the entry criteria, whether eligible or not, is to be completed by the investigator.

6.2 Treatment Period (Cycle 1) – Day 1

6.2.1 DE Phase Subjects

DE phase subjects will be admitted to the unit for pre-study safety blood, PD and immune parameter analysis and urine testing, pregnancy testing (if applicable), weight, vital signs, ECG and EDSS on Day 1

Subjects will undergo a physical examination (including neurological) and vital signs. Any medical history and concomitant medications taken since screening will be recorded and eligibility confirmed.

An indwelling intravenous cannula will be sited in a forearm.

A light breakfast will be consumed approximately 30 minutes prior to the scheduled dosing time.

Study drug will be drawn up in accordance with instructions in the Operations Manual and administered at the scheduled time by a slow intravenous infusion over 10 minutes. Subjects will be closely monitored for changes in their health or physiological status. At any time during dosing, the infusion rate can be reduced or the infusion turned off in response to an adverse event occurring. Once the infusion is completed, the cannula will be flushed with 0.9% sodium chloride for injection. The cannula will stay *in situ* until discharge on Day 2.

Subjects will be required to remain in bed for four hours post dose and then will be allowed to mobilise gently around the unit.

Vital signs will be repeated mid-way through the infusion (5 mins), 15 and 30 minutes, 2,4, 6, 8, 10, 12, 16, 20 and 24 hours post-dose. ECGs will be repeated 30 minutes, 1, 2, 4, 6, 8, 12 and 24 hours post-dose. Adverse event enquiry will be completed mid-way through the infusion (5 mins), 15 and 30 minutes, 2, 4, 6, 8, 10, 12, 16, 20 and 24 hours post dose and ongoing throughout the inpatient stay.

Meals, snacks and drinks will be provided throughout the day and subjects will remain overnight on the unit.

A 24-hour post-dose blood sample for PD and immune parameter sampling will be obtained the morning of Day 2 by venesection (**not via the indwelling cannula**). The cannula can be removed and the subject discharged from the unit if well.

6.2.2 DC Phase Subjects

DC phase subjects will undergo the identical procedures, and in addition will undergo a screening ophthalmologic examination to consist of visual acuity determination, biomicroscopy, and dilated fundoscopy; and testing for MSFC, FSS, and SF-36.

6.3 Treatment Period (Cycle 1) – Days 8, 15 and 22 (± 1) – All Subjects

All DE and DC phase subjects will be admitted to the unit the morning of dosing for a brief physical examination (including neurological examination and EDSS where clinically indicated), and vital signs, ECG, weight (Day 22 only) and pregnancy testing if applicable (Day 22 only). Any adverse events and concomitant medications taken since the last visit will be recorded and eligibility confirmed.

An indwelling intravenous cannula will be sited in a forearm. A pre-dose blood sample will be taken for safety samples, PD and immune parameter analysis.

Study drug will be administered at the scheduled time by a slow intravenous infusion over 10 minutes. Subjects will be closely monitored for changes in their health or physiological status. At any time during dosing, the infusion rate can be reduced or turned off in response to an adverse event occurring. Once the infusion is completed, the cannula will be flushed with 0.9% sodium chloride for injection. The cannula will stay *in situ* until discharge.

Subjects will be required to remain in bed for four hours post dose and then will be allowed to mobilise gently around the unit.

Vital signs will be repeated mid-way through the infusion (5mins), 15 and 30 minutes, 2 and 4 hours post-dose. ECGs will be repeated 30 minutes, 1, 2 and 4 hours post-dose. Adverse event enquiry will be completed mid-way through the infusion, 15 and 30 minutes post dose and ongoing throughout the inpatient stay.

Meals, snacks and drinks will be provided throughout the day.

Subjects will be discharged mid afternoon but may remain in the unit for up to 24 hours at the discretion of the Investigator.

Subjects will return the following morning for a 24-hour post-dose blood sample for PD and immune parameter blood sampling.

6.4 Treatment Period (Cycles 2 – 3) – Days 1, 8, 15 and 22 (± 1) – DC Phase Subjects Only

DC phase subjects will be admitted to the unit the morning of dosing for a brief physical examination (Day 1 only), weight (Day 1 and Day 22), ECG and vital signs. Any adverse events and concomitant medications taken since the last visit will be recorded and eligibility confirmed. An MSFC, FSS, and EDSS will be performed on Day 1 of each cycle, and the SF-36 will be performed at the end of Cycle 3 only. An ophthalmologic examination, to consist of visual acuity determination, biomicroscopy, and dilated fundoscopy, will be performed within a week of the end of Cycle 3. (EDSS will be repeated following neurological examination at any visit where indicated by a change in functional status).

A cranial MRI will be performed at the end of Cycle 3 (details to be provided in the companion Radiology Manual of Operations).

An indwelling intravenous cannula will be sited in a forearm. A pre-dose blood sample will be taken for safety samples (Days 8 and 22 in Cycles 2 and 3, and End of Dosing or Early Discontinuation where applicable). A pre-dose blood sample for PD and immune parameter analysis will be taken on Day 22 of each cycle.

Study drug will be drawn up and administered at the scheduled time by a slow intravenous infusion over 10 minutes. Subjects will be closely monitored for changes in their health or physiological status. At any time during dosing, the infusion rate can be reduced or turned off in response to an adverse event occurring. Once the infusion is completed, the cannula will be flushed with 0.9% sodium chloride for injection. The cannula will stay *in situ* until discharge.

Subjects will be required to remain in bed for four hours post dose and then will be allowed to mobilise gently around the unit.

Vital signs will be repeated mid-way through the infusion (5 mins), 15 and 30 minutes, 2 and 4 hours post-dose. ECG will be repeated at 4 hours post-dose. Adverse event enquiry will be completed mid-way through the infusion, 15 and 30 minutes post dose and ongoing throughout the inpatient stay.

Meals, snacks and drinks will be provided throughout the day.

Subjects will be discharged mid afternoon but may remain in the unit for up to 24 hours at the discretion of the Investigator.

6.5 Treatment Period -End of Dosing (Day 29) or Early Discharge- (7 ±1 days following last dose of drug)

6.5.1 DE Subjects

DE phase subjects will attend the unit as outpatients for the End of Dosing (Cycle 1 Day 29 ± 2) or the Early Discharge visit. A full physical examination will be performed and evaluation of EDSS, weight, vital signs, safety blood and urine samples, pregnancy testing if applicable, ECG, adverse events and concomitant medications will be completed.

A cranial MRI will be performed at the End of Dosing.

6.5.2 DC Subjects

DC phase subjects will attend the unit as outpatients for the End of Dosing (Cycle 3 Day 29 ± 2) or the Early Discharge visit. A full physical examination will be performed and evaluation of weight, vital signs, safety blood and urine samples, pregnancy testing if applicable, ECG, MSFC, FSS, FS-36, EDSS, adverse events and concomitant medications will be completed.

A cranial MRI will be performed at the End of Dosing.

6.6 Follow-up (15 Days Post-End of Dosing) – All Subjects

All DE and DC phase subjects will attend the unit as outpatients for the Follow-up visit. A full physical examination will be performed and evaluation of weight, vital signs, safety blood and urine samples, pregnancy testing if applicable, ECG, adverse events and concomitant medications will be made.

Table 2 Schedule of Events (DE Phase)

		Clinic Visit									
		Day								End of Dosing *or* Early Discharge: All Subjects	Follow-Up (15 ± 2 Days Post-End of Dosing): All Subjects
**Study Procedures**	**Pre-Study (-28 days)**	**1 Baseline**	**2**	**8 ± 1 day**	**9**	**15 ± 1 day**	**16**	**22 ± 1 day**	**23**	**Day 29 ± 2 days**	
Medical history	X										
Physical examination	X[1]	X[2,3]		X[2]		X[2]		X[2]		X[1]	X[2]
Neurological examination[4]	X	X[3]								X	
Inclusion/exclusion criteria	X										
Informed consent	X										
Body weight	X	X[3]						X		X	X
Vital signs[5]	X	X		X		X		X		X	X
Clinical laboratory testing[6]	X	X[3]		X		X		X		X	X

1 Thorough examination pre-study and at end of study dosing.

2 Symptom-directed examination weekly and at follow-up.

3 If pre-study assessment was performed within 3 days prior to baseline, assessments do not need to be repeated prior to initiation of dosing.

4 Neurological examination will be conducted at screening, baseline and end of dosing, and at other timepoints when indicated by history and adverse events.

5 Temperature, pulse, respiration, blood pressure pre-study; prior to dosing, midway through (5 minutes), and 15 and 30 minutes, 2 and 4 hours post-dosing (and 6, 8, 10, 12, 16, 20 and 24 hours post-dose on Day 1) at each clinic visit, and at follow-up.

6 Hematology (red blood cell [RBC] count, hemoglobin, hematocrit, mean corpuscular volume [MCV], mean cell haemoglobin concentration (MCHC), platelet count, white blood cell [WBC] count, differential cell count [absolute and percentage; neutrophils, lymphocytes, monocytes, eosinophils, basophils], and reticulocyte count [absolute and percentage], coagulation (prothrombin time [PT], partial thromboplastin time [PTT], INR), serum chemistry (conjugated bilirubin, alanine aminotransferase [ALT], aspartate aminotransferase [AST], alkaline phosphatase, lactate dehydrogenase [LDH], gamma-glutamyltransferase, amylase, lipase, total protein, albumin, globulin, sodium, potassium, chloride, bicarbonate, calcium, adjusted calcium, phosphate, magnesium, glucose, urea,

Study Procedures	Pre-Study (-28 days)	Clinic Visit									
		Day								End of Dosing *or* Early Discharge: All Subjects	Follow-Up (15 ± 2 Days Post-End of Dosing): All Subjects
		1 Baseline	2	8 ± 1 day	9	15 ± 1 day	16	22 ± 1 day	23	Day 29 ± 2 days	
ECG[7]	X	X		X		X		X		X	X
Pregnancy test[8]	X	X						X		X	X
EDSS[9]	X	X								X	
Cranial MRI	X									X	
Concomitant medications	X	X		X		X		X	X	X	X
Adverse events		X		X		X		X		X	X
Study Drug Administration		X		X		X		X			
PD & Immune parameter sampling **Pre-each dose**		X[10]		X[10]		X[10]		X[10]			
PD & Immune parameter sampling **24hrs Post-each dose**			X[11]		X[11]		X[11]		X[11]		

creatinine, follicle stimulating hormone [FSH; at pre-study for postmenopausal women only]), and urinalysis (pH, specific gravity, protein, glucose, ketones, white blood cells, red blood cells, leucocyte esterase, urobilinogen, bilirubin, culture where applicable) pre-study, prior to the first dose, weekly, at End of Dosing, and at Follow-Up. Serology for Hepatitis B, C and HIV and urine drug of abuse at screening only.

7 Performed pre-study, pre-dose and 30 minutes, 1, 2, 4 hours (and 6, 8, 12 and 24 hours on Day 1) post-dose, at end of study dosing, and at follow-up.

8 Screening (serum); otherwise, urine dipstix, confirmed with serum hCG if equivocal.

9 EDSS will be assessed at Screening, Baseline, End of Dosing and at other timepoints when clinically indicated by neurological examination

10 3 x 10 mL whole blood sample collected pre-each dose

11 3 x 10 mL whole blood sample collected 24 hours post each dose

Table 3 Schedule of Events (DC Phase – Cycle 1)

Study Procedures	Pre-Study (-28 days)	Clinic Visit (Cycle 1)								
		Day								
		1 Baseline	2	8 ± 1 day	9	15 ± 1 day	16	22 ± 1 day	23	Early Discharge: All Subjects
Medical history	X									
Physical examination	X[1]	X[2,3]		X[2]		X[2]		X[2]		X[1]
Neurological examination[4]	X	X[3]								X
Ophthalmologic examination[5]	X									X
Inclusion/ exclusion criteria	X									
Informed consent	X									
Body weight	X	X[3]						X		X
Vital signs[6]	X	X		X		X		X		X
Clinical laboratory testing[7]	X	X[3]		X		X		X		X

1 Thorough examination pre-study and at end of study dosing.

2 Symptom-directed examination weekly during the first cycle, once each subsequent cycle, and at follow-up.

3 If pre-study assessment was performed within 3 days prior to baseline, assessments do not need to be repeated prior to initiation of dosing.

4 Neurological examination will be conducted at screening, baseline and end of dosing, and at other timepoints when indicated by history and adverse events.

5 Visual acuity determination, biomicroscopy, and dilated fundoscopy during screening and within a week of the Early Discharge.

6 Temperature, pulse, respiration, blood pressure pre-study; prior to dosing, midway through (5 minutes), and 15 and 30 minutes, 2 and 4 hours (and 6, 8, 10, 12, 16, 20 and 24 hours post-dose on Day 1) post-dosing at each clinic visit, and at follow-up.

7 Hematology (red blood cell [RBC] count, hemoglobin, hematocrit, mean corpuscular volume [MCV], mean cell haemoglobin concentration [MCHC], platelet count, white blood cell [WBC] count, differential cell count [absolute and percentage; neutrophils, lymphocytes, monocytes, eosinophils, basophils], and reticulocyte count [absolute and percentage], coagulation (prothrombin time [PT], partial thromboplastin time [PTT], INR), serum chemistry (conjugated bilirubin, alanine aminotransferase [ALT], aspartate aminotransferase [AST], alkaline phosphatase, lactate dehydrogenase [LDH], gamma-glutamyltransferase (GGT), amylase, lipase, total protein, albumin, globulin, sodium, potassium, chloride, bicarbonate, calcium, adjusted calcium, magnesium, phosphate, glucose, urea, creatinine, , follicle stimulating hormone [FSH; at pre-study for postmenopausal women only]), and

Study Procedures	Pre-Study (-28 days)	Clinic Visit (Cycle 1)								
		Day								
		1 Baseline	2	8 ± 1 day	9	15 ± 1 day	16	22 ± 1 day	23	Early Discharge: All Subjects
ECG[8]	X	X		X		X		X		X
Echocardiogram	X									
Pregnancy test[9]	X							X		X
EDSS[10]	X	X								X
MSFC		X								X
FSS		X								X
SF-36		X								X
Cranial MRI	X									X
Concomitant medications	X	X		X		X		X	X	X
Adverse events		X		X		X		X		X
Study Drug Administration		X		X		X		X		
PD & Immune parameter sampling **Pre-each dose**		X[11]		X[9]		X[9]		X[9]		
PD & Immune parameter sampling **24hrs Post-each dose**			X[12]		X[10]		X[10]		X[10]	

---

urinalysis (pH, specific gravity, protein, glucose, ketones, white blood cells, red blood cells, leucocyte esterase, urobilinogen, bilirubin, culture where applicable) pre-study, prior to the first dose, weekly during Cycle 1, at End of Dosing, and at Follow-Up (all subjects). For DC phase subjects only, Days 8 and 22 during Cycles 2 and 3 and at the end of Cycles 4 and 6. Serology for Hepatitis B, C and HIV and urine drug of abuse at screening only.

8 Performed pre-study, pre-dose and 30 minutes, 1, 2, 4 hours (and 6, 8, 12 and 24 hours on Day 1) post-dose, at end of study dosing, and at follow-up.

9 Screening (serum); otherwise, urine dipstix, confirmed with serum hCG if equivocal.

10 EDSS will be assessed at Screening, Baseline, Early Discharge and at other timepoints when clinically indicated by neurological examination

11 3 x 10 mL whole blood sample collected pre-each dose

12 3 x 10 mL whole blood sample collected 24 hours post each dose

Table 4 Schedule of Events (DC Phase – Cycles 2-3)

	Clinic Visit (Cycles 2-3)					
	Days (each cycle)					
Study Procedures	1<br>± 1 day	8<br>+ 1 day	15<br>± 1 day	22<br>± 1 day	End of Study Dosing *or* Early Discharge: All Subjects	Follow-Up (15 +2 Days Post-End of Dosing): All Subjects
Physical examination	X[13]				X[1]	X
Neurological examination[14]	X				X	
Ophthalmologic examination[15]					X	
Body weight					X	X
Vital signs[16]	X	X	X	X	X	X
Clinical laboratory testing[17]		X		X	X	X
ECG[18]	X	X	X	X	X	X
Pregnancy test[19]				X	X	X

13 Symptom-directed examination once each cycle and at follow-up.

14 Neurological examination will be conducted on Day 1 of each cycle, at End of Study Dosing or Early Discharge and at other timepoints as indicated by history and adverse events.

15 Visual acuity determination, biomicroscopy, and dilated fundoscopy within a week of End of Dosing or Early Discharge.

16 Temperature, pulse, respiration, blood pressure pre-study; prior to, midway through, and 15 and 30 minutes, 2 and 4 hours post-dosing at each clinic visit, and at follow-up.

17 Hematology (red blood cell [RBC] count, hemoglobin, hematocrit, mean corpuscular volume [MCV], mean cell haemoglobin concentration [MCHC], platelet count, white blood cell [WBC] count, differential cell count [absolute and percentage; neutrophils, lymphocytes, monocytes, eosinophils, basophils,], and reticulocyte count [absolute and percentage], coagulation (prothrombin time [PT], partial thromboplastin time [PTT], INR), serum chemistry (conjugated bilirubin, alanine aminotransferase [ALT], aspartate aminotransferase [AST], alkaline phosphatase, lactate dehydrogenase [LDH], gamma-glutamyltransferase (GGT), amylase, lipase, total protein, albumin, globulin, sodium, potassium, chloride, bicarbonate, calcium, adjusted calciumphosphate calcium, magnesium, glucose, urea, creatinine, follicle stimulating hormone [FSH; at pre-study for postmenopausal women only]), and urinalysis (pH, specific gravity, protein, glucose, ketones, white blood cells, red blood cells, leucocyte esterase, urobilinogen, bilirubin).

18 Pre-dose and 4 hours post dose on each occasion

Study Procedures	Clinic Visit (Cycles 2-3)					
	Days (each cycle)					
	1<br>± 1 day	8<br>+ 1 day	15<br>± 1 day	22<br>± 1 day	End of Study Dosing *or* Early Discharge: All Subjects	Follow-Up (15 +2 Days Post-End of Dosing): All Subjects
EDSS[20]	X				X	
MSFC[8]	X				X	
FSS[8]	X				X	
SF-36					X	
Cranial MRI					X	
Concomitant medications	X	X	X	X	X	
Adverse events	X	X	X	X	X	
Study Drug Administration	X	X	X	X		
PD & Immune parameter sampling				X[21]		

[19] Screening (serum); otherwise, urine dipstix, confirmed with serum hCG if equivocal.

[20] Expanded Disability Status Scale (EDSS), MS Functional Composite (MSFC), and Fatigue Severity Scale` (FSS) performed on Day 1 of each cycle and at end of study dosing and at other timepoints where indicated by neurological examination.

[21] 3 x 10 mL whole blood sample collected pre-dose on Day 22 of each cycle.

7. Safety Assessments

The primary endpoint is to determine the safety profile and MTD of MIS416. Assessments will include characterization of DLTs; characterization of the type, incidence, severity, timing, seriousness and relationship to treatment of AEs; effects on vital signs and laboratory parameters; changes in ECGs and ophthalmologic examinations.

7.1 Adverse events

The definition of an adverse event is given in section 10.2.

Adverse events will be monitored throughout the study and recorded on the AE page of the CRF. Adverse events will be graded according to the Toxicity Grading Scale for Healthy Adult and Adolescent Volunteers Enrolled in Preventive Vaccine Clinical Trials (Guidance for Industry) issued by US DHHS September 2007 and modified to include a systemic line item for rigors/chills. The relationship of adverse event to study drug will be determined by the investigator and details of this classification is given in section 8.

7.2 History and Physical and Neurological Examinations

At screening, all subjects will undergo a medical history and physical examination. A focused physical examination will be repeated weekly during the first cycle, once each subsequent cycle, at end of dosing or early discharge and at follow up (15 days post end of dosing). A neurological examination will be performed at screening, at Day 1 of each cycle, and at end of dosing or early discontinuation.

Unscheduled physical and neurological assessments will be conducted at other timepoints when clinically indicated by history and adverse events.

7.3 Ophthalmologic Examinations

In the DC phase only, subjects will undergo visual acuity determination, biomicroscopy, and dilated fundoscopic examination at screening and within a week of the End of Study or Early Withdrawal visit.

7.4 Safety Laboratory Tests

Blood and urine samples will be taken for safety laboratory assessment (haematology, biochemistry, serology and urinalysis) as described in Tables 2, 3 and 4. The tests will be performed at a local laboratory according to their laboratory standard operating procedures. The normal ranges will be supplied to the investigator and sponsor prior to the start of the study. The following tests will be included in each panel:

Haematology: Red blood cells, haemoglobin, haematocrit, mean cell volume, mean cell haemoglobin, mean cell haemoglobin concentration, platelet count, white blood cell count, reticulocytes (absolute and percentage), differential (absolute and percentage): neutrophils, lymphocytes, monocytes, eosinophils, basophils.

Coagulation: Prothrombin Time (PT), Activated Partial Thromboplastin time (APTT), INR

Biochemistry: Sodium, potassium, calcium, glucose, albumin, globulin, adjusted calcium, phosphate, bicarbonate, chloride, magnesium, aspartate aminotransferase, alanine aminotransferase, alkaline phosphatase, gamma- glutamyl transferase, lactate dehydrogenase,conjugated bilirubin, total protein, plasma creatinine, urea, amylase, lipase, follicle stimulating hormone (screening only where applicable), quantitative hCG (where applicable), Urinalysis:Protein, glucose, pH, leucocyte esterase, white blood cells, red blood cells, ketones, urobilinogen, , specific gravity, bilirubin, culture (if applicable), qualitative hCG (where applicable)

Urine drug of abuse screen: Opiates, amphetamines, cannabinoids, benzodiazepines, methadone

Serology: Hepatitis B, hepatitis C, HIV

A total of up to approximately 343 mL of blood is required during the DE phase and approximately 506 mL of blood is required during the DC phase with both quantities including PD and immune parameter sampling, per Section 7.7.

7.5 Vital Signs

Vital signs (including systolic and diastolic blood pressure, heart rate, respiratory rate and temperature) will be determined at screening, on each visit day, as described in Tables 2, 3 and 4 and at follow up. Vital signs will be measured after the subject has been lying supine for at least 5 minutes.

7.6 ECG and Echocardiogram

A 12-lead ECG will be recorded for all subjects at screening, pre-dose and 30 minutes, 1, 2 and 4 hours (and 6, 8, 12 and 24 hours on Day 1) post-dose in Cycle 1, at the end of study dosing, and at follow up. For DC phase subjects only, ECG will be recorded pre-dose and 4 hours post dose on each subsequent dosing day.

An ECG will be recorded after the subject has been lying supine for at least 5 minutes. The date and time the ECG was recorded, as well as the protocol and subject identification details should be recorded on the printout.

The following parameters will be recorded in the CRF: heart rate, PR, QRS, QT, QT_{cF} as well as any abnormality in rhythm or wave morphology.

In the DC phase only, subjects will undergo echocardiography during screening.

7.7 Body Weight, Height and BMI

Body weight will be measured at screening, Day 1 and 22 of Cycle 1, end of dosing or early discharge and follow up. For DC phase subjects only, body weight will be recorded once at the end of Cycles 2-3. Height will be measured at screening only. BMI will be calculated by the following formula:

$$BMI = \text{Body weight (kg)}/\ (\text{height})^2\ (m^2)$$

8. Pharmacodynamic (PD) & immune parameter sampling

Blood samples will be taken for PD and immune parameter assessment as described in Tables 2, 3 and 4. The tests will be performed by the Sponsor according to their laboratory standard operating procedures and may include some or all of the following.

Peripheral blood monocytes (PBMC production of IL-12p40, IL-12p70, IL-23, TNFα and IL-6 in response to stimulation with LPS/IFNγ ex vivo). Immune parameters (Serum IFNγ, IL-17, IL-6, TNFα and GM-CSF levels; PBMC expression of mRNA encoding IL-12p40, IL-12p35, IL-23p19, LTα, LTβ, IFNγ, IL-17, GM-CSF, RORγt, and Tbet; PBMC production of IFNγ, IL-17, and GM-CSF in response to stimulation with anti-CD3 and anti-CD28 ex vivo).

9. Pharmacogenomic Analysis

Following specific written consent from the subjects, at least one aliquot of PBMC sample will be sent to Dr David Booth, University of New South Wales for pharmacogenomic analysis. Samples will be stored for up to 25 years to permit investigation into genes of interest related to the immune system, thought to be associated with susceptibility to MS and other autoimmune diseases. The value of the research lies in the responsiveness or not to immune modulation with MIS416 may further validate some of the hypotheses being developed relating to immune system-related gene polymorphisms and susceptibility to disease.

10. Clinical Status Assessments

DC phase subjects will be assessed at the end of study for MS clinical status.

The appearance or reappearance of one or more clinical neurologic deficits, persisting for at least 48 hours following a period of relatively stable or improving status lasting at least 30 days, and/or loss of function reflected by an increase in EDSS, and/or the presence of new, expanding, or gadolinium-enhancing lesions (based on total T2 lesion burden) will be regarded as worsening clinical status.

The order in which these assessments are to be done will be as follows: MSFC, FSS, SF-36 and EDSS.

10.1 Multiple Sclerosis Functional Composite (MSFC)

Instructions for the administration and scoring of the Multiple Sclerosis Functional Composite[19,20] are given in the MSFC Administration and Scoring Manual, National Multiple Sclerosis Society, 2001. It is presented in the companion Operations Manual.

The MSFC should be administered as close to the beginning of the study visit as possible and definitely before the patient does a distance walk. Every effort should be made to use the same testing room and designated area for the timed 8-meter walk at every visit. It is essential that the potential for distractions to be kept to a minimum and for all obstacles to be removed for the Timed 8-meter walk. All necessary materials should be assembled prior to the arrival of the subject.

10.2 Fatigue Severity Scale (FSS)

The Fatigue Severity Scale [(22)] is a self-administered questionnaire to be completed by the subject. It is presented in the companion Operations Manual.

10.3 Short Form Health Survey SF-36

The Short Form Health Survey (SF-36)[24] is a self-administered questionnaire to be completed by the subject. It is presented in the companion Operations Manual.

10.4 Expanded Disability Status Scale (EDSS)

The Expanded Disability Status Scale[21,24,25] will be completed by the physician following neurological examination. It is presented in the companion Operations Manual.

10.5 Cranial Magnetic Resonance Imaging (MRI)

Details of the protocol for cranial MRI scanning will be provided in the companion Operations Manual. MRI will be used to assess both safety and clinical status parameters.

11. Investigational Product

11.1 Dosage and Schedule of Test Drug

Four ascending dose levels of MIS416 (125, 250, 500 and 1000 µg) are planned at this stage. Treatment is open label. Dosing will proceed as described in section 4.1.

11.2 Maximum Tolerated Dose

The MTD will be declared by the Safety Review Team, when at least 2 out of six subjects in a cohort experience a DLT. The MTD will be determined as the next lower dose in which <2 subjects experienced a DLT.

11.3 Formulation, Packaging and Labeling

MIS416 is a semi synthetic non toxic, non immunogenic, rod shaped microparticle (0.2 x 2.0 microns) composed of muramyl dipeptide repeats covalently attached to a poly amino acid backbone principally composed of lysine and glycine amino acid repeats. N-acetyl glucosamine is incorporated as an oxidizable carbohydrate for use in linking amino containing ligands. Single-stranded bacterial DNA is incorporated through amino linkages covalently attached to the poly amino backbone. Microparticle components are isolated from *Propionibacterium acnes*.

MIS416 is formulated in solution in 0.9% sodium chloride for injection at 0.2 mg/mL, packaged into 7 mL sterile glass vials and stored at 2° to 8° C.

11.4 Preparation and Administration of Test Drug

MIS416 will be administered intravenously into a peripheral vein. Administration will be as a slow intravenous infusion, over 10 minutes, using an infusion pump.

The required volume of drug will be drawn up from the vial and made up to 10 mL total volume with 0.9% sodium chloride for injection. The infusion must be made up no longer than 8 hours prior to administration. If the infusion is to be made up several hours prior to administration, then it should be stored in the refrigerator at 2° to 8° C and removed and brought up to room temperature just prior to administration.

Just prior to setting up the infusion, the syringe should be vigorously shaken for 30 seconds.

The infusion pump will be set at a rate of 1 mL/min, so that the dose is administered over 10 mins. Once the infusion is completed, the line will be flushed with 20 mL 0.9% sodium chloride for injection.

11.5 Accountability of Drug Supplies

The Sponsor will supply study drug to the investigational site. Drug supplies provided for this study will be manufactured under Good Manufacturing Practices, by the Sponsor, in Auckland, New Zealand, pursuant to a licence to manufacture medicines issued by Medsafe.

The study medication will be dispensed by the Site Investigator/Pharmacist.

Receipt of study drug, dispensing and administration logs will be kept by the pharmacist/investigator in the Investigator/Pharmacy files. At the end of the study, study drug will be reconciled and a copy of the record given to the CRA/sponsor. Surplus supplies of study drug will be returned to the Sponsor.

11.6 Recall of Investigational Product

In case of recall of investigational product (decided by the regulatory authority or the sponsor), the investigators will immediately be informed by the sponsor. The investigator will urgently attend to the following:

- Stop administration of the product to the subjects.
- Inform the concerned subjects.
- Contact the General Practitioners of the affected subjects
- Terminate the study

The sponsor will arrange for the return of the recalled product, according to their standard operating procedures.

12. Safety Issues

12.1 Warnings and Precautions

No evidence available at the time of completion of this study protocol indicated any special warnings or precautions were appropriate, other than those listed in the Investigator’s Brochure.

12.2 Adverse Events and Laboratory Abnormalities

12.2.1 Clinical Adverse Events

An Adverse Event is any untoward medical occurrence in a patient or clinical investigation subject administered a pharmaceutical product and which does not have to have a causal relationship with this treatment. An Adverse Event can therefore be

any unfavourable and unintended sign, symptom, or disease temporally associated with the use of a medicinal product, whether or not considered related to the medicinal product. Pre-existing conditions that worsen during a study are to be reported as Adverse Events.

An adverse event is any adverse change from the subject's baseline condition, including any clinical or clinically significant laboratory test value abnormality that occurs during the course of the clinical study after the experimental drug has been utilized, whether the adverse event is considered related to the treatment or not.

12.2.2 Severity Assessment

All clinical Adverse Events (AEs) encountered during the study will be reported on the AE page of the CRF. Intensity of the AEs will be classified in accordance with Guidance for Industry Toxicity Grading Scale for Healthy Adult and Adolescent Volunteers Enrolled in Preventative Vaccine Clinical Trials (issued by US DHHS September 2007) ("preferred term") and modified to include a systemic line item for rigors/chills.

12.2.3 Laboratory Test Abnormalities

Laboratory test results will appear on electronically produced laboratory reports submitted directly from the central laboratory and be recorded in the laboratory results pages of the CRF. Laboratory test value abnormalities as such should not be reported on the AE page of the CRF as AEs unless they result in a clinically relevant condition.

12.2.4 Serious Adverse Events (Immediately Reportable to Sponsor)

The definitions and reporting requirements of ICH Guidelines for Clinical Safety Data Management, Definitions and Standards for Expedited Reporting, Topic E2, will be adhered to.

An Adverse Event is regarded as serious if it is one of the following:

- Fatal
- Life-threatening
- Results in persistent or significant disability/incapacity
- Requires inpatient hospitalisation or prolongs hospitalisation
- Is a congenital anomaly/birth defect
- Clinically significant, requiring medical or surgical intervention to avoid one of the above.

Any AE that is serious, occurring during the course of the study and within 28 days of the end of the study, irrespective of the treatment received by the subject, and whether drug-related or not, must be reported to the Sponsor's Medical Monitor or designee,

within 24 hours of the site becoming aware of it (expedited reporting). Reporting to the Sponsor must be made irrespective of the extent of the available AE information. Additional follow up information relating to the serious AE must be reported in a similarly expedited manner once it is available.

In addition, the investigator may be requested by the Sponsor or designee to obtain specific additional follow-up information in an expedited manner. This information may be more detailed than that captured on the AE CRF. In general, this will include a description of the AE in sufficient detail to allow for a complete medical assessment of the case and independent determination of possible causality. In the case of a subject death, a summary of post-mortem examination findings must be submitted as soon as possible to the Sponsor.

If a serious AE occurs, the following personnel are to be notified by fax within 24 hours of awareness of the event by the investigator:

Dr Michael Silverman, Medical Monitor:

Fax	+1 781 989 3227
Tel	+1 781 631 8596 or +1 781 639 1349
Email	msilverman@biostrategics.com

12.2.5 Treatment of Adverse Events

AEs that may be related or possibly related to a drug with immune modulation mechanisms of action may include anaphylactic reaction or acute pro-inflammatory cytokine release.

- Management of anaphylaxis should follow appropriate guidelines and recommendation such as those made by the Resuscitation Council of New Zealand, 2006
- Acute pro-inflammation should be treated with appropriate fluid replacement, anti-inflammatory drugs (steroidal and non-steroidal) and antihistamines.

12.2.6 Causality Assessment

The Investigator's assessment of causality must be provided for all AEs (serious and non-serious). An investigator's causality assessment is the determination of whether there exists a reasonable possibility that the investigational product caused or contributed to an AE. For this assessment, investigators must categorize the causality as unrelated, possibly related, probably related or related according to the following definitions:

Unrelated: There is evidence that the AE definitely has an etiology other than the assigned study drug.

Possibly Related: The adverse event has a temporal relationship to study drug administration. However, an alternative etiology may be responsible for the AE. Information on drug/product withdrawal may be lacking or unclear.

Probably Related: The AE has a temporal relationship to study drug administration. The event is unlikely to be related to an alternative etiology. There is a reasonable response on withdrawal (dechallenge).

Related: The adverse event has a temporal relationship to study drug administration and resolves when the drug is discontinued. An alternative etiology is not apparent.

12.2.7 Follow-up of Adverse Events

AEs, especially those for which the relationship to study drug is not “unrelated”, should be followed-up until they return to baseline status or stabilised. If a clear explanation is established it should be recorded on the CRF.

In the event of unexplained clinically relevant abnormal laboratory test values, the tests should be repeated immediately and followed-up until they have returned to the normal range and/or an adequate explanation of the abnormality is found. If a clear explanation is established, it should be recorded on the CRF.

12.2.8 Pregnancy

Pregnancy occurring in the partner of a subject participating in the study, if the investigator becomes aware of it, should also be reported to the sponsor within 24 hours. A Pregnant Partner Consent Form is provided to document discussions with the partner of the study participant and to obtain her consent to collect follow up information regarding the pregnancy. The partner should be counseled regarding the possible effects to the fetus and monitoring of the subject should continue until conclusion of the pregnancy, where consent to do so is given.

13. Criteria for Premature Withdrawal

Subjects have the right to withdraw from the study at any time for any reason.

The investigator also has the right to withdraw subjects from the study in the event of intercurrent illness, AEs, protocol violations, administrative reasons or other reasons.

Should a subject decide to withdraw, all efforts should be made to complete and report the follow-up procedures, particularly the physical examination, as thoroughly as possible.

Subjects who have prematurely withdrawn may be replaced, at the discretion of the investigators and with the approval of the sponsor, if withdrawal occurs for reasons other than DLT or an SAE related to study drug.

14. Statistical Considerations

The statistical analysis plan and biostatistical analysis is provided by Westat (Houston, TX).

14.1 Sample Size and Determination of MTD

There are no formal statistical calculations to determine the sample size. The total number of subjects to be enrolled in the study is dependent upon the observed safety profile, which will determine the number of subjects per dose level, as well as the number of dose escalations required to achieve the MTD. It is anticipated that approximately 27-33 subjects will be enrolled, with approximately 12-18 subjects in the dose-escalation phase and up to additional 15 subjects in the dose-confirmation phase.

The table below gives the probabilities of dose escalation at any stage during the MTD determination, as a function of the true, but unknown underlying DLT rate at the current dose level.

Underlying DLT rate	10%	20%	30%	40%	50%	60%	70%	80%	90%
Probability of dose escalation	91%	71%	49%	31%	17%	8%	3%	1%	0.1%

14.2 Analysis Populations

14.2.1 Intent-to-Treat (ITT) Population

All subjects who were screened and met the eligibility requirements, regardless of their compliance with the treatment per protocol will be in the ITT analysis. A subject may have baseline measurements, but may not receive treatment or have any follow-up data. However, this subject will be part of the analysis.

14.2.2 Safety Evaluable (SE) Population

The SE population consists of subjects receiving any amount of MIS416. The estimation of toxicity rates will be based on the SE population.

14.2.3 Clinical Status (CS) Population

The CS population includes subjects who have been on the trial for at least 2 months and have had end-of-Cycle 2 clinical status assessments recorded.

14.3 Data Handling

14.3.1 Measurement Times

14.3.1.1 Visit Windows

Visit windows will not be used. The nominal visit time point entered on the case report forms will be used. Unscheduled assessments, if any, will be listed, but will not be included in tabulations by visit.

14.3.1.2 Baseline Values

All assessments on Day 1, before the first dose of the study drug, will be considered as Baseline. If the value at Baseline is missing, the last value from the Screening visit will be used as Baseline. If there are multiple Baseline assessments, the most recent one will be used for analysis.

14.3.2 Missing Data Conventions

Unless otherwise specified, missing data will be considered missing at random and will not be imputed. However, imputation of partial dates may be performed during the data analysis and will be documented.

14.4 Statistical Methods

14.4.1 General Overview and Plan of Analysis

Summary statistics will be presented to describe the analysis populations, namely, the ITT, SE, and CS populations. Tabulations will include the number of subjects, mean, standard deviation, minimum, median, and maximum for continuous variables; and/or frequency and percentage of subjects for categorical variables. Summary statistics for certain safety parameters will also be presented for the MTD population.

For all subjects, the data will be tabulated by dose cohort and for the total patient population.

All statistical analyses will be performed using SAS version 9.1 (or higher), unless specified otherwise. The use of other statistical software will not be considered a violation of the statistical analysis plan, nor require an amendment of this plan.

14.4.2 Hypothesis Testing

There is no formal hypothesis testing in this study.

14.4.3 Interim Analysis

There is no planned interim analysis in this study, unless periodic reports are requested by the Sponsor before the trial begins.

14.5 Statistical Analysis

14.5.1 Disposition of Subjects

The analyses of subject disposition will be performed on the ITT population. The number and percentage of subjects will be presented by number of cycles of study drug completed. The number of subjects who completed follow-up and those who terminated early from treatment or the study, along with their reasons for early termination, will be presented. Reasons for termination from the study are described in the protocol.

14.5.2 Baseline Characteristics

14.5.2.1 Demographic Characteristics

Summary statistics for demographic characteristics will be presented for the ITT population (or SE population, if all subjects received the study drug). Tabulations for age, sex, ethnicity, and race will be presented. Age (years) will be calculated as the integer part of ((date of screening – date of birth + 1)/365.25).

14.5.2.2 Physical Characteristics

Summary statistics will be presented for height (cm), weight (kg), and BMI at Screening for the ITT population.

14.5.2.3 Clinical Status

Summary statistics for the following measures of clinical status will be presented for the ITT population.

- Multiple Sclerosis Functional Composite (MSFC) score
- Fatigue Severity Scale (FSS) score
- Short Form Health Survey (SF-36) score
- Expanded Disability Status Scale (EDSS) score
- MRI findings

- Self-reported and clinically confirmed clinical exacerbations

14.5.3 Analysis of Safety and Tolerability

The analyses of safety endpoints will be conducted on the SE population. Statistical tests will not be performed. The safety profile and MTD of MIS416 will be determined. Adverse events, changes in laboratory measurements, and results of other safety evaluations will be presented. Toxicity rates will be determined.

14.5.3.1 Extent of Exposure

Summary statistics will be presented for the number of infusions and the cumulative drug dose received by subjects in each dose cohort. A corresponding listing will also be generated to show the number of infusions at each dose level, and the total infusions and cumulative drug dose received by each subject.

14.5.3.2 Adverse Events (AE)

“Adverse event” and “adverse experience” may be used interchangeably. All summary tables will be based on coded preferred terms, instead of verbatim terms. All adverse events will be coded, using MedDRA dictionary version 11.0. The use of another version will not be considered a violation of the statistical analysis plan, nor require an amendment to the plan. The categories and definitions of severity and causal relationship for all AEs, including the criteria for which an AE is to be classified as “serious”, are as described in the protocol.

The overall incidence of adverse events will be tabulated by dose cohort and total population, including the number and percentage of subjects with at least one: adverse event, serious adverse event, or treatment-related adverse event. The number of deaths and discontinuations due to adverse event(s) will be presented. The incidence of adverse events by intensity/grade (Grades 1, 2, 3, and 4) and by relationship to study drug (not related, possibly related, and probably related), and the incidence of serious adverse events (SAEs) by relationship to study drug, will also be presented. DLTs will be identified in the AE/SAE listings. If there are multiple occurrences of the same AE for the same subject, only the first occurrence will be counted.

14.5.3.3 Clinical Laboratory Assessments

Summary statistics will be presented for Baseline laboratory measurements, including hematology, chemistry, coagulation, and urinalysis. Normal ranges for the laboratory parameters will be provided by the laboratory which performed the assessments. All normal ranges will be standardized and results will be reported in standard units. The tables will include summary statistics for the Baseline assessments and the changes from Baseline to each subsequent timepoint of measurement. Shift tables will be generated for each laboratory parameter (except urinalysis), showing the transitions

from Baseline assessment to normal (within normal range), low, or high value on each subsequent timepoint. A listing of subjects with abnormal laboratory assessments will be presented.

14.5.3.4 Vital Signs

Vital signs, including changes from baseline to each subsequent timepoint, will be summarized for each dose level using descriptive statistics.

14.5.3.5 Electrocardiogram (ECG) Findings

ECG findings, including changes from baseline to each subsequent timepoint, will be summarized for each dose level using descriptive statistics. The number and percentage of subjects with clinically significant abnormal ECG findings at any visit will be tabulated.

14.5.3.6 Radiological Findings

Radiological (MRI) findings, including changes from baseline to each subsequent timepoint, will be summarized at each dose level for the following parameters:

- Gadolinium-enhancing lesions (number and volume)
- Total T2 lesion volume
- Brain parenchymal fraction

14.5.4 Analysis of Clinical Status

Analyses of clinical status endpoints will be conducted on the CS population. Summary statistics will be presented for baseline measurements and changes from baseline at each dose level for the following:

- MSFC score
- FSS score
- SF-36 score
- EDSS score
- Self-reported and clinically confirmed clinical exacerbations

Time to EDSS (TTE) deterioration will be determined as follows:

Date of first of two confirmed deteriorations from baseline minus Date of 1st Study Drug Administration + 1 day. TTE deterioration will be calculated in days, but will be reported in days or months.

14.5.5 Analysis of Pharmacodynamics (PD)

Summary statistics for PD profiles of the subjects in each dose cohort and the total population will be presented in a separate Pharmacodynamics Report. Absolute and percent changes from baseline for each subsequent timepoint will be tabulated.

The pharmacodynamic parameters will include some or all of the following: serum IFN., IL-17, IL-6, TNFa and GM-CSF levels; PBMC expression of mRNA encoding IL-12p40, IL-12p35, IL-23p19, LTa, LTß, IFN, IL-17, GM-CSF, ROR.t, and Tbet; PBMC production of IFN, IL-17, and GM-CSF in response to stimulation with anti-CD3 and anti-CD28 ex vivo; PBMC production of IL-12p40, IL-12p70, IL-23, TNFa and IL-6 in response to stimulation with LPS/IFN ex vivo. These serum and cellular findings during the dose-escalation phase will be presented. Depending on the dose-escalation findings, a selective or comprehensive set of PD parameters will similarly be summarized for the dose-confirmation phase.

14.5.6 Exploratory Analyses

Any correlations between clinical, radiological, and PD outcomes will be evaluated.

15. Ethical Aspects

15.1 Local Regulations/Declaration of Helsinki

The investigator will ensure that this study fully adheres to the principles outlined in "Guideline for Good Clinical Practice" ICH Tripartite Guideline (January 1997) which has as its basis in the principles of the "Declaration of Helsinki" (2008) or with the laws and regulations of the country in which the research is conducted, whichever affords the greater protection to the public.

15.2 Informed Consent

It is the responsibility of the investigator, or a person designated by the investigator, to obtain written informed consent from each individual participating in this study after adequate explanation of the aims, methods, objectives and potential hazards of the study. It must also be explained to the subjects that they are completely free to refuse to enter the study or to withdraw from the study at any time for any reason. Appropriate forms for obtaining informed consent will be provided by the investigator.

If new safety information results in significant changes in the risk/benefit assessment, the consent form should be reviewed and updated if necessary. All subjects (including all those already treated) should be informed of the new information, given a copy of the revised form and asked to give their consent to continue in the study.

15.3 Independent Ethics Committee

This protocol and any accompanying material provided to the subject (such as Information Sheets) as well as any advertising or compensation given to the subject, will be submitted by the investigator to an Independent Ethics Committee. Approval from the committee must be obtained before starting the study and should be documented in a letter to the investigator specifying the date on which the committee met and granted approval.

Any modifications made to the protocol after receipt of Ethics Committee approval must also be submitted to the Ethics Committee in accordance with local procedures and regulatory requirements.

16. Conditions for Modifying the Protocol

Protocol modifications to ongoing studies must be made only after consultation between an appropriate representative of the sponsor and the investigator. Protocol modifications must be agreed in writing and signed by the investigator and sponsor.

All protocol modifications must be submitted to the appropriate Ethics Committee for information and approval in accordance with local requirements and Medsafe. Approval must be obtained before any changes can be implemented, except for changes necessary to eliminate an immediate hazard to study subjects, or when the change(s) involves only logisitical or administrative aspects of the study (eg, change in monitor(s) or telephone numbers.

17. Conditions for Terminating the Study

Both the sponsor and the investigator reserve the right to terminate the study at any time. Should this be necessary, both parties will arrange the procedures on an individual study basis after review and consultation. In terminating the study, the Sponsor and the investigator will assure that adequate consideration is given to the protection of the subject's interests.

The study can be terminated for (including, but not limited to) the following reasons:

1. A significant safety concern arising from either ongoing pre-clinical or clinical studies
2. Inability to recruit the required number of subjects to the study
3. Strategic termination of the development programme
4. Any other reason required by the sponsor or investigator

18. Study Documentation, CRFs and Data Handling

18.1 Investigator's Files/Retention of Documents

The investigator shall supply the sponsor on request with any required background data from the study documentation or clinic records. This is important when CRFs are illegible or when errors in data transcription are suspected. In case of special problems and/or governmental queries or requests for audit inspections, it is also necessary to have access to the complete study records, provided that subject confidentiality is protected.

The Investigator's File will contain the protocol/amendments, Case Report and Query Forms, Ethics Committee and Medsafe approval with correspondence, sample informed consent, drug records, staff curriculum vitae and delegation logs and other appropriate documents/correspondence, etc.

Clinical source documents will be defined in advance to record key efficacy/safety parameters independent of the CRF. They include subject clinical records, physician's and nurses' notes, original laboratory reports, ECGs, signed Consent Forms and subject screening and enrolment logs. The investigator must keep these two categories of documents on file for at least 15 years after completion or discontinuation of the study. After that time, the documents may be destroyed, subject to local regulations. Should the investigator wish to assign the study records to another party or move them to another location, the sponsor will be notified in advance.

The investigator shall supply the sponsor on request with any required background data from the study documentation or clinic records. This is important when CRFs are illegible or when errors in data transcription are suspected. In case of special problems and/or governmental queries or requests for audit inspections, it is also necessary to have access to the complete study records, provided that subject confidentiality is protected.

18.2 Confidentiality of Trial Documents and Subject Records

The investigator must be aware that subjects' anonymity will be maintained and that their identities are protected from unauthorised parties. On CRFs or other documents submitted to the sponsor, subjects should not be identified by name, but by an identification code. The investigator should keep a subject enrolment log relating codes to the names of subjects. The investigator should maintain documents not for submission to the sponsor, eg, subjects' written consent forms, in strict confidence.

18.3 Case Report Forms (CRFs)

It is the intent of this study to acquire study data via electronic format; however, should the electronic capture system be unavailable, paper CRF forms will be utilised.

As part of the responsibilities assumed by participating in the study, the investigator agrees to maintain source documentation (eg, ECGs), to enter subject data into the electronic CRF (eCRF) as accurately as possible, and to respond to any reported discrepancies rapidly. eCRFs are accessed through the Trial Forms (Primorus Clinical Trials Ltd) remote data capture application, which allows for on-site data entry and data management. Site users can read from and write to the sponsor's database where the clinical data are collected. This provides immediate, direct data transfer to the database, as well as immediate detection of discrepancies, enabling site coordinators to resolve and manage discrepancies in a timely manner. Each person involved with the study at each site will have an individual identification code and password that allows for record traceability. Thus, the system, and subsequently any investigative reviews, can identify coordinators, investigators, and individuals who have entered or modified records.

For each subject enrolled, a CRF must be completed and signed a Principal Investigator or authorised delegate from the study staff. This also applies to records for those subjects who fail to complete the study (even during a pre-randomisation screening period if a CRF was initiated). If a subject withdraws from the study, the reason must be noted on the CRF. If the subject is withdrawn from the study because of a treatment-limiting AE, thorough efforts should be made to clearly document the outcome.

All paper forms should be filled out using indelible ink and must be legible. Errors should be crossed out but not obliterated, the correction inserted and the change initialed and dated by the investigator and/or his/her authorised delegate. The investigator should ensure the accuracy, completeness, legibility and timeliness of the data reported to the sponsor in the CRFs and in all required reports.

18.4 Data Handling and Record Keeping

The full details of procedures for data handling will be documented in the Data Management Plan. Adverse events, medical history, and concurrent conditions will be coded using Medical Dictionary for Regulatory Activities (MedDRA). Drugs will be coded using the World Health Organization (WHO) Drug dictionary (WHODrug).

18.5 Assignment of Preferred Terms and Original Terminology

For classification purposes, preferred terms will be assigned by the sponsor to the original terms entered on the CRF, using the latest version of MedDRA (medical dictionary for regulatory activities terminology) for AEs and diseases and the WHODrug (World Health Organisation Drug dictionary) drug terms and procedures dictionary for treatments and surgical and medical procedures.

18.6 Study Centre Monitoring Visits

Monitoring visits to the study site will be made periodically during the study to ensure that all aspects of the protocol are followed. It is understood that the responsible monitor will contact and visit the investigator regularly and will be allowed, on request, to inspect the various records of the study (CRFs and other pertinent data) provided that subject confidentiality is maintained in accord with local requirements.

Source documents will be reviewed for verification of data recorded on the eCRFs. Source documents are defined as original documents, data, and records.

The investigator and his/her delegates agree to cooperate with the monitor to ensure that any problems detected in the course of these monitoring visits are resolved.

18.7 Quality Assurance and Regulatory Agency Audits

The study site also may be subject to quality assurance audits by the sponsor or designees. In this circumstance, the sponsor-designated auditor will contact the site in advance to arrange an auditing visit. The auditor may request to visit the facilities where laboratory samples are collected, where the medication is stored and prepared, and any other facility used during the study.

In addition, there is the possibility this study may be inspected by regulatory agencies (Medsafe), including those of foreign governments (eg, the US Food and Drug Administration [FDA], the United Kingdom Medicines and Healthcare products Regulatory Agency [MHRA]). If the study site is contacted for an inspection by a regulatory body, the sponsor should be notified immediately. The investigator and institution guarantee access to source documents by the sponsor or its designee (CRO) and by the IRB or EC or regulatory body.

19. Publication of Data and Protection of Trade Secrets

The results of this study may be published or presented at scientific meetings. If this is foreseen, the investigator agrees to submit all manuscripts or abstracts to the Sponsor at least 30 days prior to submission. This allows the sponsor to protect proprietary information and to provide comments based on information from other studies that may not yet be available to the investigator.

Any formal publication of the study in which input of the Sponsor’s personnel exceeded that of conventional monitoring will be considered as a joint publication by the investigator and the appropriate sponsor personnel. Authorship will be determined by mutual agreement.

20. References

1. Fallon P. G., Alcami A., (2006) Pathogen-Derived Immunomodulatory Molecules. Future Immunotherapeutics? *Review TRENDS in Immunol 27* (10)
2. Gay N. J., Gangloff M. (2007) Structure and Function of Toll Receptors and Their Ligands, *Annu. Rev. Biochem. (76*).141-65
3. Akira S., Uematsu S., Takeuchi O. (2006) Pathogen Recognition and Innate Immunity. *Cell 124* (Feb 24) 783-801
4. Tomai M. A., Miller R. L., Lipson K. E., Vasilakos J. P., Woulfe S. L. (2006) Immune Response Modifiers. Imiquimod and Future Drugs for Modulating the Immune Response. Drug Discovery Today. *Therapeutic Strategies 3* (3)
5. Sirard J-C, Vignal C., Dessein R., Chamaillard M. (2007) Nod-Like Receptors: Cytosolic Watchdogs for Immunity Against Pathogens. *PLoS Pathogens Vol 3* (12)
6. Kerkmann M., Rothenfusser S., Hornung V., Towarowski A., Wagner M., Sarris A., Giese T., Endres S., Hartmann G. (2003) Activation with CpG-A and CpG-B Oligonucleotides Reveals Two Distinct Regulatory Pathways of Type I IFN Synthesis in Human Plasmacytoid Dendritic Cells. *J. Immunol. 2003 (170)* 4465-447
7. Immune system's Toll-like receptors have a good opportunity for cancer treatment. J. National Cancer Institute, 2006:98(9);574-575.
8. E.C.A. Darcissac, G.M. Bahr, P.R. Pouillart, G.J. Riveau and M.A. Parant Selective potentiation of cytokine expression in human whole blood by Murabutide, a muramyl dipeptide analogue.. Cytokine 1996:8(8);658-666.
9. G.M.Bahr, E.C.A. Darcissac, P.R. Pouillart, L.A. Chedid. Synergistic effects between recombinant Interleukin-2 and the synthetic immunomodulatory Murabutide: Selective enhancement of cytokine release and potentiation of antitumor Activity. J. Cytokine and Interferon Research 1996:16(2);169-178.
10. L.A.J. O'Neill and A.G. Bowie.The family of five: TLR-domain-containing adaptors in Toll-like receptor signaling. Nature Reviews Immunology 2007:7;353-364.
11. Tabata, Y. and Ikada, Y. (1988). Effect of the size and surface charge of polymer microspheres on their phagocytosis by macrophage. Biomaterials 9(4): 356-362.
12. Solomon, L. Z., Birch, B. R., et al. (2000). "Nonhomologous bioinjectable materials in urology: 'size matters'?" BJU International 85(6): 641-645.
13. Y. Krishnamachari, A.K. Salem. Innovative strategies for co-delivering antigens and CpG oligonucleotides. Advanced Drug Delivery Reviews 2009:61(3);205-217.
14. P. Malyala, D.T. O'Hagan, M. Singh. Enhancing the therapeutic efficacy of CpG oligonucleotides using biodegradable microparticles. Advanced Drug Delivery Reviews 2009:61(3);218-225.
15. A.D. Kimbrel and B. Beutler. The evolution and genetics of innate immunity. Nav. Rev. Genetics 2001:2;1-13.
16. O. Gutierrez, C. Pipaon, A. Fontalba, Y. Ogura, F. Prosper, G. Nunez, J.L. Fernandez-Luna. Induction of Nod2 in Myelomonocytic and intestinal epithelial cells via nuclear factor-kB activation. J. Biol. Chem. 2002: 277(44);41701-41705.

17. McDonald, W.I., et al.. Recommended diagnostic criteria for multiple sclerosis: guidelines from the International Panel on the diagnosis of multiple sclerosis. *Ann Neurol 2001 Jul;50(1):*121-7.
18. Lublin, F.D., Reingold, S.C., and National Multiple Sclerosis Society (USA) Advisory Committee on Clinical Trials of New Agents in Multiple Sclerosis. Defining the clinical course of multiple sclerosis: Results of an international survey. *Neurology 1996 46*: 907-911.
19. Cutter, G.R., Baier, M.S., Rudick, R.A., Cookfair, D.L., Fischer, J.S., Petkau, J., Syndulko, K., Weinshenker, B.G., Antel, J.P., Confavreux, C., Ellison, G.W., Lublin, F., Miller, A.E., Rao, S.M., Reingold, S., Thompson, A., & Willoughby, E. (1999). Development of a Multiple Sclerosis Functional Composite as a clinical trial outcome measure. *Brain* 122, 101–112.
20. http://www.nationalmssociety.org/for-professionals/researchers/clinical-study-measures/msfc/index.aspx (accessed 26 May 2010)
21. Kurtzke J.F. Rating neurologic impairment in multiple sclerosis: an expanded disability status scale (EDSS). *Neurology. 1983 Nov;33(11)*:1444-52.
22. Kurtzke J F et al. Studies on the natural history of multiple sclerosis 6. Clinical and laboratory findings at first diagnosis. *Acta Neurologica Scandinavica* (1972), 48(1), 19-46Krupp L B et al. The Fatigue Severity Scale. Application to patients with Multiple Sclerosis and Systemic Lupus Erythematosus. *Arch Neurol* 1989 (46):1121-3.
23. Fischer J.S., LaRocca N.G,, Miller D.M., Ritvo P.G., Andrews H, Paty D. Recent developments in the assessment of quality of life in multiple sclerosis (MS). *Mult Scler. 1999 Aug;5(4)*:251-9.
24. http://www.sf-36.org/tools/SF36.shtml#LITLITHaber A, LaRocca N.G. (eds). *Minimal Record of Disability for multiple sclerosis*. New York: National Multiple Sclerosis Society; 1985.
25. http://www.nationalmssociety.org/for-professionals/researchers/clinical-study-measures/fss-and-edss/index.aspx (accessed 26 may 2010)

Appendix 1 McDonald Criteria for Diagnosis of Multiple Sclerosis

Clinical Presentation	Additional Data Needed
* 2 or more attacks (relapses)<br>* 2 or more objective clinical lesions	None; clinical evidence will suffice (additional evidence desirable but must be consistent with MS)
* 2 or more attacks<br>* 1 objective clinical lesion	Dissemination in space, demonstrated by:<br>* MRI<br>* or a positive CSF and 2 or more MRI lesions consistent with MS<br>* or further clinical attack involving different site
* 1 attack<br>* 2 or more objective clinical lesions	Dissemination in time, demonstrated by:<br>* MRI<br>* or second clinical attack
* 1 attack<br>* 1 objective clinical lesion (monosymptomatic presentation)	Dissemination in space demonstrated by:<br>* MRI<br>* or positive CSF and 2 or more MRI lesions consistent with MS<br>**and**<br>Dissemination in time demonstrated by:<br>* MRI<br>* or second clinical attack
Insidious neurological progression suggestive of MS (primary progressive MS)	One year of disease progression (retrospectively or prospectively determined) and<br><br>Two of the following: a. Positive brain MRI (nine T2 lesions or four or more T2 lesions with positive VEP) b. Positive spinal cord MRI (two focal T2 lesions) c. Positive CSF

Appendix 2 New York Heart Association Functional Classification (1994)

NYHA Class	Symptoms
I	No symptoms and no limitation in ordinary physical activity, e.g. shortness of breath when walking, climbing stairs etc.
II	Mild symptoms (mild shortness of breath and/or angina) and slight limitation during ordinary activity.
III	Marked limitation in activity due to symptoms, even during less-than-ordinary activity, e.g. walking short distances (20-100 m).<br>Comfortable only at rest.
IV	Severe limitations. Experiences symptoms even while at rest. Mostly bedbound patients.

Appendix 3 Toxicity Grading Scale for Healthy Adult and Adolescent Volunteers Enrolled in Preventative Vaccine Clinical Trials

(Source: Guidance for Industry issued by U.S. DHHS September 2007)

The tables within the guidance document do not include a systemic line item for rigors/chills. Rigors/chills should be graded in accordance with the following scale:

Systemic (General)	Mild (Grade 1)	Moderate (Grade 2)	Severe (Grade 3)	Potentially Life Threatening (Grade 4)
Rigors/Chills	Mild	Mild to Moderate, no antipyretic medication (paracetamol/NSAID) indicated	Moderate, antipyretic or corticosteroid indicated	Severe, symptomatic >4 hours despite treatment with antipyretic /corticosteroid

The systemic line item for pyrexia has been amended in line with existing guidelines for adverse events associated with oncology studies (ECOG (http://ecog.dfci.harvard.edu/general/ctc.pdf) and NHI CTEP (http://ctep.cancer.gov/protocolDevelopment/electronic_applications/ctc.htm)).

Systemic (General)	Mild (Grade 1)	Moderate (Grade 2)	Severe (Grade 3)	Potentially Life Threatening (Grade 4)
Fever (^{o}C)	37.1 – 38.0	38.1 – 40	>40 for less than 24 h	>40 for more than 24h despite prophylaxis or treatment with antipyretics (paracetamol or NSAID)

[The Guidance for Industry document "Toxicity Grading Scale for Healthy Adult and Adolescent Volunteers Enrolled in Preventative Vaccine Clinical Trials" is presented in the companion Operations Manual]
